# Avalanche-like intercalation and intraparticle correlations in graphite

**DOI:** 10.1038/s41586-026-10862-4

**Published:** 2026-07-29

**Authors:** Jiho Han, George S. Phillips, Alice J. Merryweather, Juhwan Lim, Christoph Schnedermann, Robert L. Jack, Clare P. Grey, Akshay Rao

**Affiliations:** 1 Cavendish Laboratory, University of Cambridge, Cambridge, UK; 2 Yusuf Hamied Department of Chemistry, University of Cambridge, Cambridge, UK; 3 The Faraday Institution, Harwell Science and Innovation Campus, Didcot, UK; 4 Illumion, Cambridge, UK; 5 DAMTP, Centre for Mathematical Sciences, University of Cambridge, Cambridge, UK

**Keywords:** Batteries, Materials chemistry, Phase transitions and critical phenomena, Batteries

## Abstract

Although graphite is the most widely used negative electrode material in lithium-ion batteries^1^, its lithium insertion processes and associated dynamics, particularly those of the dilute stages, remain poorly understood. A fundamental understanding of how symmetry-breaking phase transitions occur continuously under operating conditions is lacking. Here, using operando optical microscopy, we provide a unified picture of ion intercalation dynamics during the dilute stages of graphite intercalation, showing that the graphitic particles undergo rapid, localized deintercalation–intercalation step events, leading to deintercalation–intercalation of micrometre-sized regions within seconds. These are reminiscent of a phase-transition phenomenon, ‘avalanches’, which occurs in disordered materials, involving step changes in the order parameter due to jumps between multiple metastable states^2,3^. Using a modified random field Ising model, the avalanches are related to static disorder, which disrupts intercalation dynamics. The model can also account for the apparently continuous transitions between stages and the experimental avalanche statistics. Finally, we develop a methodology to spatio-temporally analyse the sequences of avalanche events, revealing considerable heterogeneous connectivity. Our work highlights the role of local and static disorder in explaining unexpected phase-transition behaviour and provides new tools and concepts for studying layered battery materials.

## Abstract (web summary)

Operando optical microscopy shows avalanche-like lithium deintercalation (intercalation) processes as the dilute stages of graphite are emptied (filled), showing how local disorder governs phase-transition dynamics and ion transport in lithium-ion battery electrodes.

## Main

Owing to its layered structure, graphite undergoes lithium intercalation by a series of distinct transitions between stages, identified by their stage number ‘*N’* denoting that every *N*th inter-graphene gallery contains lithium. Lithiation begins with a dilute stage 1′L and then proceeds by stages 4L → 3L → 2L → 2 → 1 (refs. ^4–6^). The suffix ‘L’ indicates a dilute phase with no lithium ordering in the filled gallery, and stage 1 corresponds to the well-known, closely packed configuration of LiC_6_.

Previous studies on graphite have resulted in contradictory reports on phase composition during operando cycling, particularly in the low lithium-concentration ‘dilute stages’^6–9^. Delithiation–lithiation processes in battery materials are often categorized as continuous^10–13^ or phase separating^14–17^, in which lithiation in graphite has been reported to have both first-order biphasic and continuous transitions^4–6^, although many structural studies have shown that these categories are not so clearcut, particularly under operating conditions^4,6^. These processes are usually studied electrochemically and with advanced operando techniques such as X-ray, neutron or electron diffraction. However, the dilute stages in graphite are particularly difficult to study because of low atomic weights, rapid lithium diffusion^18,19^, metastable phase formation, particle-level heterogeneity^20,21^ and problems with beam damage during measurement. This has led to the dilute stages being poorly characterized in comparison with the dense stages, despite their important role in fast charging^18^.

Here, we investigate the sub-second lithiation dynamics in an individual active particle of graphite during the dilute stages (up to *x* ≈ 0.25 in Li_*x*_C_6_) as a function of temperature using the recently developed method of charge photometry, an operando optical scattering microscopy. We show that delithiation–lithiation of the dilute stages proceeds by a series of intermittent, step events, a process that is fundamentally distinct from our previous optical microscopy observations of first-order biphasic behaviour in Li_*x*_CoO_2_ (ref. ^22^) and solid-solution diffusion in LiNi_*x*_Mn_*y*_Co_*z*_O_2_ (refs. ^23,24^) single particles. These step events resemble crackling noise or ‘avalanches’^2,3,25,26^ seen across a wide range of material systems, including Martensitic transformations^27^, magnetization^28^, ferroelectric switching^29,30^ and fluid invasion^31,32^. We use this framework to develop an anisotropic random field Ising model (RFIM), and simulate lithium filling in a disordered graphite lattice. This qualitatively captures the experimental avalanche statistics and the apparently continuous behaviour of the 4L–3L transition^4,6,7^. Finally, using the cross-correlation function, we demonstrate that the seemingly random sequence of avalanches possesses definite spatio-temporal correlation patterns, which are related to the disorder identified in the electron backscatter diffraction (EBSD) map.

## Optical signatures of graphite lithiation

**Fig. 1:**
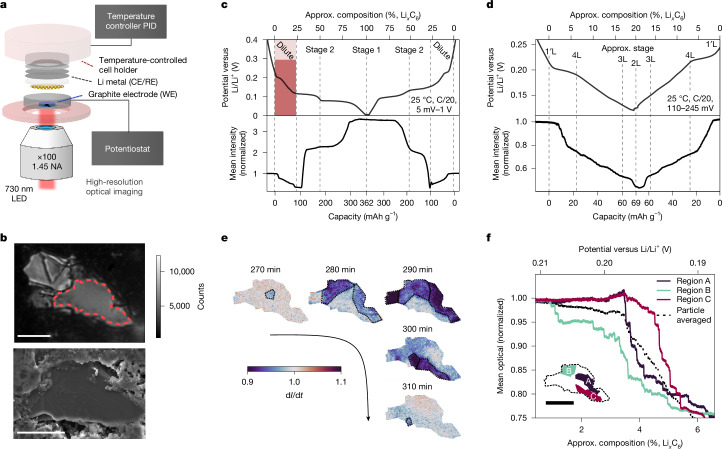
Experimental set-up and observation of spatially local discrete intensity jumps. **a**, Experimental set-up consisting of an optical microscope and an optically accessible coin half-cell. Graphite electrode (50 wt% active mass) was used as the working electrode (WE), and the lithium metal as counter electrode (CE) and reference electrode (RE). The cell is connected to a temperature-controlled cell holder and potentiostat (full schematic in Supplementary Fig. 1). **b**, Optical image of the active graphite particle (top). The dotted red region indicates the area of the particle analysed in this work. Ex situ SEM image of the same graphite particle (bottom). **c**, Potential profile during a full cycle (top). The red-highlighted region indicates the dilute-stages regime. Particle-averaged (red-dotted region in **b**) optical scattering intensity during cycling (bottom). Vertical dashed lines (in **c**,**d**) indicate the stage expected at a given time or potential. The values are normalized to initial intensity (in **c**,**d**,**f**). **d**, Potential profile during cycling between 110 mV and 245 mV at C/20 rate at 25 °C (top). Particle-averaged optical scattering intensity during cycling (bottom). **e**, Sequential differential contrast images of the graphite particle during the dilute stage transition (stage 1′L to 4L). Images are obtained by dividing respective frames every 30 s. Blue indicates an instantaneous decrease, and red indicates an increase in optical intensity. **f**, Mean optical intensity during stage 1′L to 4L transition for distinct graphite regions. Particle regions are shown in the bottom left for reference. Top *x*-axis indicates the interpolated cell potential. LED, light-emitting diode; NA, numerical aperture. Scale bars, 5 μm (**b**,**f**).

To study the ion dynamics of graphite at a single particle level, an optically accessible half-cell (coin cell format) with a self-standing graphite electrode was prepared and imaged using an optical scattering microscope^22^ equipped with a temperature-controlled cell holder (Fig. 1a). An optical image of a representative particle is shown in Fig. 1b (top) alongside its scanning electron microscopy (SEM) image taken after the experiment (Fig. 1b, bottom), highlighting a single graphite particle embedded in the electrode. The studied region of the particle (Fig. 1b top, red-dotted line) was confirmed to correspond to the basal plane of a single graphite particle, from high-resolution SEM and EBSD mapping (Supplementary Information section 1.1.1).

During the formation cycle, the electrode was initially cycled against a lithium metal counter electrode to 5 mV compared with Li/Li^+^, to achieve full lithiation (*x* = 1, Li_*x*_C_6_) at a specific current of 19 mA g^−1^ or C/20 (here *n*C denotes the current required to fully discharge or charge the cell to its theoretical capacity in 1/*n* hours). The cell achieved a discharge capacity of 362 mAh g^−1^, close to the theoretical capacity of 372 mAh g^−1^. The potential profile, shown in Fig. 1c (top), exhibits the familiar staging behaviour of graphite, with dilute stages occurring from 205 mV to 115 mV (up to *x* ≈ 0.24), followed by the stage 2L → 2 transition at a plateau around 112 mV (up to *x* ≈ 0.5) and the stage 2 → 1 plateau around 77 mV (*x* = 0.5–1).

Figure 1c (bottom) shows the corresponding optical scattering intensity obtained during this cycle for the highlighted flake (Fig. 1b, top). The mean intensity initially drops with lithiation in the dilute stages, then increases substantially in intensity at the stage 2L → 2 and stage 2 → 1 transitions, both of which are biphasic, first-order phase transitions according to diffraction studies. The optical response of graphite during lithiation^33^ can be explained in terms of increasing electron carrier density with lithiation, resulting in a plasma frequency (*ω*_p_) blue-shift and increased metallic reflection. Importantly, the optical intensity is a direct probe for the local lithiation state as discussed previously^23,34^. During the dilute stages, the intensity decreases monotonically during lithiation (Supplementary Information section 1.3.1), with a penetration depth of at most about 100 layers and within the particle depth (Supplementary Information section 1.3.2).

**Extended Data Fig. 1:**
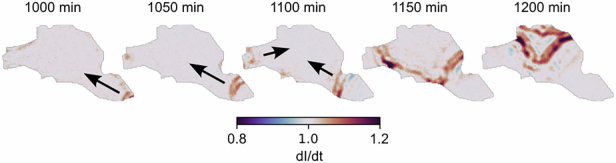
Phase transition during stage 2 to 1. Sequential differential contrast images of the graphite particle during the transition from dense stage 2 to 1. Images are obtained by dividing respective frames by another taken 30 s earlier during lithiation at 25 °C at C/20, from 1000 min to 1200 min (Fig. 1c). Blue (red) indicate an instantaneous decrease (increase) in optical scattering intensity, which we assign to the movement of the stage 1 phase front. This behaviour is consistent with an “intercalation wave” where nucleation occurs at one (or more) points, with the front moving rapidly across the whole particle as indicated by the arrows.

To gain further insight, we cycled the same cell over a smaller potential window at the same C-rate from 110 mV to 245 mV (between *x* = 0 and about 0.22), as shown in Fig. 1d (top), in which transitions between the dilute stages are expected. In this potential window, the optical scattering intensity of the graphite particle (Fig. 1d, bottom) exhibits features consistent with the expected transitions. A sharp drop in intensity occurs during the plateau around 200 mV versus Li/Li^+^, in which the first-order 1′L → 4L transition is expected. Subsequently, a seemingly continuous drop in optical intensity during the transition 4L → 3L was observed, the cell potential gradually dropping to about 120 mV. At this point, a final drop in optical intensity occurred, in line with the expected 3L → 2L transition. When imaging at sufficiently high frame rates (2 Hz or 0.0007% Li_*x*_ per frame), these seemingly continuous intensity changes were revealed to consist of a sequence of many small, sudden, discrete jumps (Fig. 1f). These step-like features in the optical intensity were observed during both delithiation and lithiation and also across cells with different graphite types (Supplementary Information section 1.2.3 and Supplementary Video 2) when cycled at the same rate (C/20). Furthermore, each step could often be attributed to a reoccurring step in a distinct, localized region of the graphite particle, as shown in Fig. 1e using differential image analysis, which highlights intensity changes over 30 s (Supplementary Video 1). This is discussed in more detail later. Figure 1f demonstrates the intensity profile of some of these local particle regions (Fig. 1f inset), further demonstrating the step-like lithiation behaviour and independence between particle regions. It is important to stress that neither the step features nor the region separation occur during the dense stage 2–1 transition. Instead, and as shown in Extended Data Fig. 1, we observe an intercalation wave mechanism^22,35^ with nucleation and growth of a new phase across the entire particle consistent with a first-order biphasic transition.

## Step events correlated with electrochemistry

**Fig. 2:**
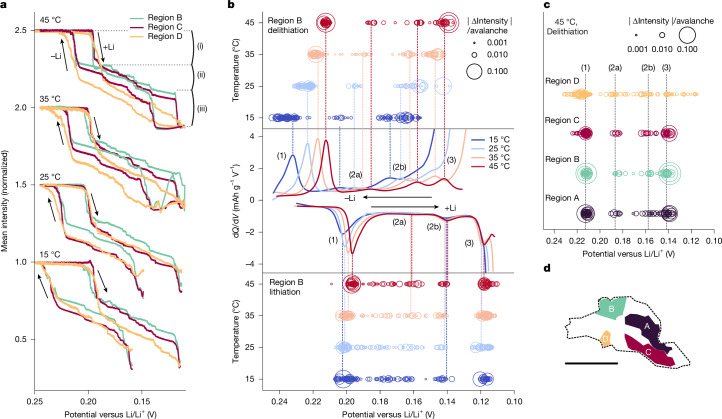
Optical step changes correlated with cell potential. **a**, Mean optical intensity curves from different particle regions compared with cell potential. Lithiation and delithiation curves are both shown, with direction denoted by black arrows, normalized to the start and end of delithiation and lithiation, respectively. Only regions B, C and D are shown for simplicity. Where appropriate, the curves have been cut before the onset of the stage 2L to 2 transition for clarity. Three regimes in the intensity trends are marked (i), (ii) and (iii). **b**, Differential capacity (d*Q*/d*V*) curves from the charge–discharge data at different temperatures (middle). Four processes labelled (1), (2a), (2b) and (3) can be distinguished. The onset of a peak corresponding to the dense transition (2L–2) is seen in these plots at temperatures 35 °C and below. The black arrows denote the direction of lithiation compared with delithiation. Scatter plot of all step events that occur during delithiation (top) or lithiation (bottom), detected from the mean optical intensity as a function of temperature (region B, cycles 1 and 2). Each circle represents one step event, with the *x*-position denoting the potential and the size denoting the intensity. **c**, Scatter plot of all detected step events in different regions of the particle (45 °C delithiation, cycles 1 and 2). The *x*-position denotes the potential, and the size denotes the intensity change. Cell potentials corresponding to processes (1), (2a), (2b) and (3) as found in **b** are marked with dotted lines. **d**, A map of particle regions, A–D, used for mean intensity calculations. Scale bar, 5 μm.

The optical response was then correlated with the cell potential in Fig. 2a for different regions of the particle (map shown in Fig. 2d) and as a function of cell temperature. The sharp–gradual–sharp intensity trend (with respective regimes labelled (i), (ii), (iii) in Fig. 2a top right) occurred both during lithiation and delithiation across all temperatures. The voltage hysteresis between the delithiation and lithiation cycles increases at lower temperatures, but the small step-like intensity changes appear throughout all datasets in all three regimes (i)–(iii).

To further quantify the intensity steps, we used a step-detection algorithm to analyse the intensity traces from individual graphite regions (Supplementary Information section 3.3). This approach allowed us to build up statistics for these events as a function of temperature, cycle, voltage and region dependence. Each detected step event is plotted as a circle against the voltage at which it occurred (Fig. 2b, top, delithiation; bottom, lithiation). The size of the plotted circle encodes the event size (that is, magnitude of the step fall or rise). For simplicity, we have limited ourselves to events in region B of the particle in this plot. The distribution of the events is then correlated with a differential capacity analysis (d*Q*/d*V*) of the cell electrochemistry, which highlights underlying phase transitions or processes. Figure 2b (middle) shows the d*Q*/d*V* plots at different temperatures, with prominent peaks marked with numbers. The first peak during lithiation at 197–202 mV (1) is attributed to the 1′L → 4L transition. There are then two small peaks around 162 mV (2a) and 140 mV (2b) (more pronounced at higher temperatures, see Supplementary Fig. 11), and an additional peak (3) is visible as a shoulder of the large truncated peak (assigned to the onset of the 2L → 2 transition). During delithiation, similar trends are observed, with the smaller peaks (2a) and (2b) becoming more prominent. Based on this analysis, the two sharp drops in optical intensity shown in Fig. 2a, (i),(iii) are attributed to the differential capacity peaks (1) and (3), respectively, whereas peaks (2a,b) occur during the more gradual intensity changes (Fig. 2a, (ii)). Furthermore, the step-like events detected in Fig. 2a (top and bottom) cluster around the d*Q*/d*V* peak positions, with the events becoming more tightly clustered at higher temperature. This is closely related to the d*Q*/d*V* peak shape, which sharpens at higher temperature.

**Extended Data Fig. 2:**
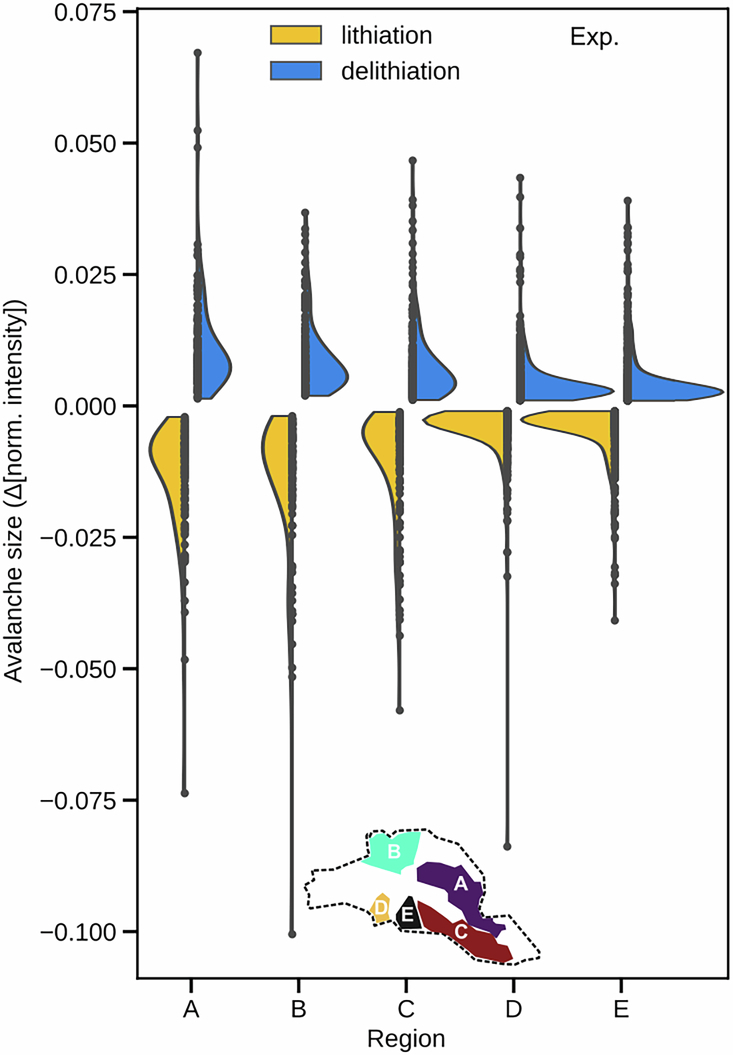
Effect of disorder on experimental event (avalanche) size distributions from different regions. Violin plots showing the detected avalanche size distribution statistics from different experimental regions during de/lithiation cycles at all temperatures, where size is defined as change in the mean optical intensity. A map of the particle regions is located at the bottom for reference.

The same analysis can be extended to other regions of the particle, and a selection is shown in Fig. 2c for the 45 °C delithiation cycle. Regions A, B and C show consistent trends as discussed, but region D shows events that are more widely distributed, even at 45 °C. This was true generally, with some regions consistently showing a broad temporal spread of events at all temperatures (Supplementary Fig. 32). As shown in Extended Data Fig. 2, these regions (D and E) also experience more frequent smaller-sized events, whereas in other regions step events are more broadly distributed in size.

**Fig. 3:**
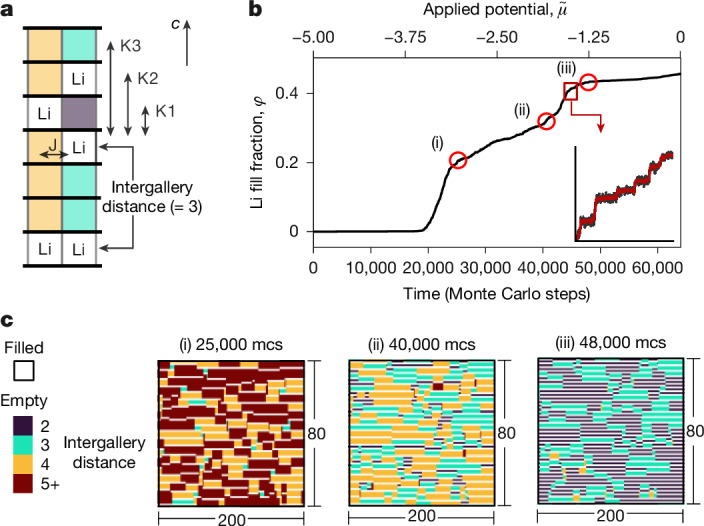
RFIM set-up and filling behaviour. **a**, Schematic of the intralayer (J) and up to third-neighbour interlayer (K1, K2 and K3) coupling terms in the 2D model, the colours indicate different distances between two Li in the *c*-direction. **b**, Mean lattice fill fraction (*ϕ*) compared with Monte Carlo steps (mcs) during a single ramped potential simulation (top), with disorder ($\mathop{h}\limits^{ \sim }$ = 0.4), and temperature ($\mathop{T}\limits^{ \sim }$ = 0.125), with total mcs = 80,000. The red circles denote time points (i)–(iii) from which the lattice pictures in **c** are taken. The red square inset highlights a portion of the filling curve, which demonstrates stepped (avalanche) behaviour (mcs = 45,700–47,200). **c**, The lattice structure shown at different time points (i)–(iii). Occupied sites are drawn in white, and unoccupied sites are coloured according to the respective *c*-axis separation between the occupied sites.

The step-like intensity features of the individual regions during the dilute stages are highly reminiscent of avalanches^2,3^: abrupt processes with a broad distribution of sizes, which occur generically in disordered systems under a slowly applied driving force. Complex experimental trends such as avalanche statistics and transition dynamics can be understood qualitatively using general theories for these phenomena. This motivated us to develop an RFIM to simulate the filling of a graphite lattice with discrete lithium occupation with attractive intralayer coupling, repulsive screened interlayer coupling^36–38^, and an on-site random potential representing idealized disorder (Fig. 3a). We considered both two-dimensional (2D) and three-dimensional (3D) models (for full description, see Supplementary Information section 2.1). Further justification and experimental comparisons can be found in Supplementary Information section 1.2. As shown in Fig. 3b, the simulated filling behaviour of this model under a slow potential ramp exhibits both avalanches (sudden jumps in fill fraction) and the sharp–gradual–sharp filling trend observed in the experiment. These arise because static disorder results in energy barriers to filling that are large enough to disrupt the dynamics of system-wide transitions between equilibrium phases at experimentally relevant timescales and temperatures. In our model, an avalanche usually corresponds to a local domain filling in a single or a few correlated layers (see Supplementary Fig. 27 and Supplementary Video 5). Our model also implies that pure staging does not exist during the reaction (Fig. 3c) because of the intrinsically nonequilibrium dynamics of avalanching systems.

**Fig. 4:**
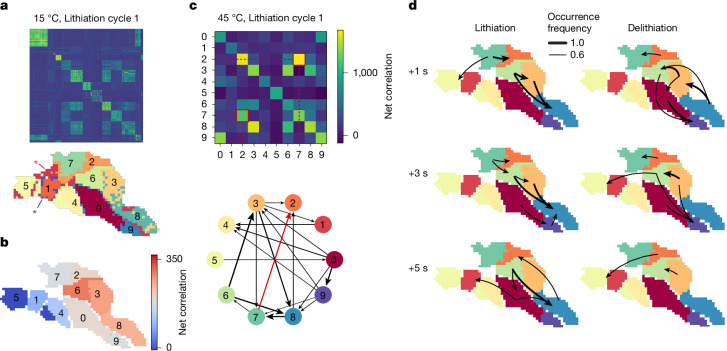
Spatio-temporal correlations during 1′L–4L transition. **a**, Demonstration of concurrent activity mapping. Cross-correlation calculated from every pair of pixels (5 × 5 binned) on the particle, using the time-differential signal (cycle 1 at 15 °C). The resultant cross-correlation function is summed for time lags (−5 s ≤ *τ* ≤ 5 s), and the resultant symmetric cross-correlation matrix is clustered into 12 blocks using spectral co-clustering (top). These 12 clusters are then mapped back into space, which produces regions on the particle that show concurrent activity (bottom). We exclude two clusters (edge pixels) in further analyses, marked with an asterisk. **b**, Identification of the most and least connected regions. The correlation function from the coarse-grained dataset (cycles 1 and 2 at 15–45 °C) is summed over time lags (−5 s ≤ *τ *≤ 5 s). All inter-region correlations are then summed, excluding self-correlation, and mapped to the particle. **c**, Demonstration of inter-region, positive time-lag correlations. The correlation function from the coarse-grained dataset (cycle 1 at 45 °C) is summed over time lag (0.5 s ≤ *τ* ≤ 3 s) and the off-diagonal elements are interpreted as successor–predecessor relationship frequency between two regions (top). The relationship is mapped as a directed, weighted graph (bottom), in which each region (node) is connected by a weighted, directed edge defined as the difference of reciprocal values (*i*,* j*) − (*j*, *i*). The arrow points from the predecessor to the successor. Twenty edges with the highest weights are shown. **d**, Time-lag-dependent intraparticle correlations during delithiation and lithiation. All predecessor–successor relationships are identified from the dataset (cycles 1 and 2 at 15–45 °C) as per **c**. The reoccurring edges are mapped onto the particle at different lag times. The potential window corresponding to the 1′L → 4L transition was used for all analyses.

**Extended Data Fig. 3:**
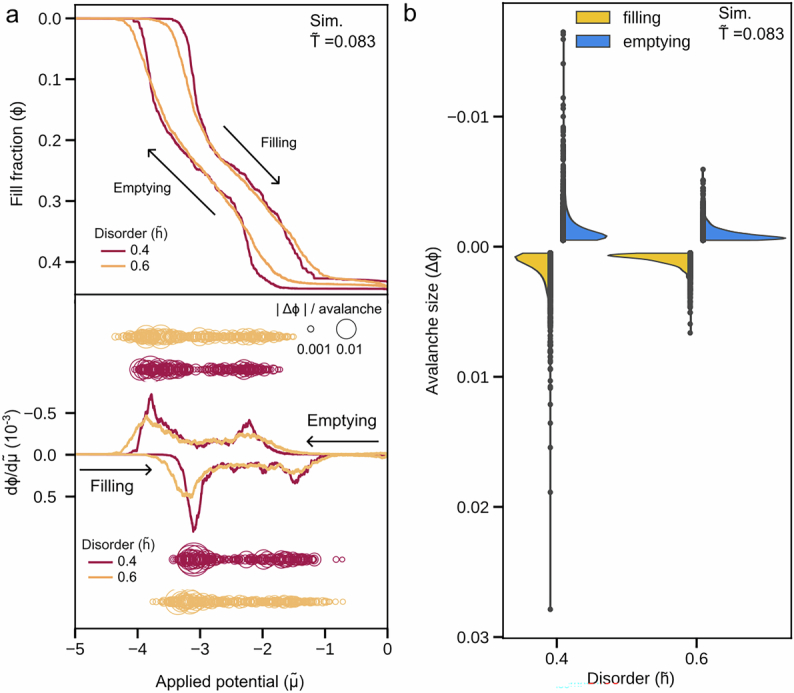
Effect of disorder on model trends. **a**, Mean lattice fill fraction (ϕ) vs applied potential ($\mathop{{\rm{\mu }}}\limits^{ \sim }$) during filling and emptying simulations for three different disorder strengths for the 2D model ($\mathop{{\rm{T}}}\limits^{ \sim }$ = 0.083, mcs = 80 k) for a single run (upper panel). The differential fill fraction per potential (dϕ/${\rm{d}}\mathop{{\rm{\mu }}}\limits^{ \sim }$) during the filling/emptying simulations for corresponding filling/emptying simulations with different disorder (lower panel). Above and below the differential plots are the corresponding detected avalanche events, plotted as a circle with its x position denoting the potential at which it occurred. Compare with experimental results in Fig. 2b,c. **b**, Violin plots showing the detected avalanche size distribution with different disorder strengths. Statistics from model, gathered from a single filling/emptying run with varying disorder strengths from 2D model ($\mathop{{\rm{T}}}\limits^{ \sim }$ = 0.083, mcs = 80 k), where size is defined as the change in mean fill fraction. Compare with experimental results in Extended Data Fig. 2. The y-axis has been reversed in order to aid comparison of simulation to experiment.

The model can be used to understand the avalanche size distribution and temporal spread in different experimental regions. For example, in Extended Data Fig. 3, we demonstrate that a higher disorder strength results in more frequent smaller avalanches and a broadened temporal spread, similar to those in the experimental particle regions D and E. The region-dependent trends (regions A–E in Fig. 2 or 0–9 in Fig. 4) seen in the optical measurements and the graphite disorder were explored using ex situ EBSD and atomic force microscopy (AFM) measurements (Supplementary Information section 1.1). The grain reference orientation deviation and the particle topology maps obtained from these two techniques, respectively, show similar regions or domains to those seen optically, with regions D and E showing a higher density of folds and bends and thus more static disorder. Further model–experiment comparisons can be found in Supplementary Information section 2.3.

We note that this model is meant as a general qualitative framework and uses an idealized representation of quenched, uncorrelated disorder as a proxy for material properties such as turbostratic disorder^39^ and elastic deformation^40^. A discussion of model limitations and potential extensions can be found in Supplementary Information section 2.5.

## Spatio-temporal correlation

As our dataset contains a series of spikes in the differential intensity time series, this opens up analysis routines frequently exploited in time-domain correlation spectroscopy. Here, we calculate the pixel–pixel pair cross-correlation function for the differential time series images. The full methodology for this section can be found in Supplementary Information section 3.2. This general method enables us to investigate our dataset for spatio-temporal correlation without relying on manual region selection or avalanche detection algorithms, and we can extract a cross-correlation matrix showing the relationship between any two pixels at a given time lag.

First, we explored the correlation matrix in which off-diagonal elements signify pixels that undergo a concerted intensity change within the defined time period of ±5 s. to which spectral co-clustering was applied to identify the correlated blocks of pixels (Fig. 4a, top). When these blocks are mapped back to the particle, spatial regions emerged (Fig. 4a, bottom), which reproduce the manually selected particle regions discussed previously. These are also well correlated to the EBSD map (Supplementary Fig. 1e–g). We repeated this analysis for different temperatures, delithiation and lithiation cycles and lag times, and the clusters remained similar (Supplementary Fig. 29). The clusters identified in Fig. 4a have a strong preference to undergo delithiation and lithiation together. However, the presence of off-block diagonal elements highlights the existence of strong inter-region correlations.

To explore these correlations, the dataset was coarse-grained into distinct regions, which were used to re-calculate the correlation function. This allowed us to identify the regions with most inter-region connectivity. This is shown in Fig. 4b, in which these coarse-grained regions are coloured according to the most and least inter-region correlations (−5 s ≤ *τ* ≤ 5 s). The results are highly consistent with our previous analysis, in which the least connected regions (Fig. 4b, regions 4 and 5) are the same as regions D and E, with more frequent smaller step events (Extended Data Fig. 2) broadly spread around d*Q*/d*V* peaks (Supplementary Fig. 32), which were correlated with deformation and kinks in ex situ studies (Supplementary Information section 1.1).

To explore specific inter-region relationships, the correlation matrix is summed over a positive time lag (3 s ≥ *τ* > 0), which signifies that an event in one region is followed by an event in another region within the time window. The resultant correlation matrix (Fig. 4c, top) is asymmetric due to one region preferring to precede the other. The results of this analysis in a directed graph network, with the directionality and weight of the edge corresponding to the time-ordered difference of the corresponding off-axis elements, as shown in Fig. 4c (bottom). The arrow then indicates a likely predecessor–successor relationship between the regions. We extend this analysis to all cycles and temperatures, in which inter-region correlations are collected at different lag times and kept only if they occur in the majority of datasets. In Fig. 4d, these relationships are shown on the particle, demonstrating that at a small time lag (*τ* ≈ +1 s), well-connected, adjacent regions become correlated, whereas these correlations become longer range at larger time lags (τ ≈ +5 s). The correlations substantially differ between lithiation and delithiation. Notably, the short-range, short-time-lag correlations are often reversed between delithiation and lithiation. This implies that the correlations are not simply due to the directionality of phase fronts as seen in the dense stages (Extended Data Fig. 1) or differences in local overpotential. Rather, each avalanche is a transition to a reoccurring, spatially localized metastable configuration, which may account for the asymmetry during delithiation and lithiation.

In summary, we have experimentally demonstrated the complexity present during lithiation in a single particle of graphite. Disorder facilitates the formation of metastable states, and what is expected to be a singular transition (nucleation and progression of a phase front) is broken spatially into smaller domains and temporally into smaller avalanche steps. The effectiveness of our simple model motivates the incorporation of disorder in more realistic, continuum models. We anticipate that the experimental, analytical and conceptual approaches developed here will advance battery materials research by enabling a rigorous understanding of phase-transition physics in realistic disordered systems.

## Methods

### Sample preparation

A self-standing graphite electrode was prepared using a modified doctor blade technique, with all casting and mixing processes conducted at room temperature under ambient conditions. Polyvinylidene difluoride powder (200 mg; Kynar HSV900, Arkema) was dissolved in 2.4 ml *N*-methyl-2-pyrrolidone (99.5% anhydrous NMP, Sigma-Aldrich) in a planetary mixer (ARM-310, Thinky) at 2,000 rpm for 10 min. Conductive carbon powder (200 mg; Super P Li, Timcal) and graphite powder (400 mg; KS44 primary synthetic graphite, Timcal) were added to the solution and mixed into a slurry at 2,000 rpm for 10 min. The slurry was cast onto a clean glass sheet manually using a doctor blade at 200 μm height, dried at 80 °C for 1 h and gently peeled with a razor. The self-standing electrode was manually punched into 6 mm discs, dried overnight in a vacuum oven at 120 °C and stored in an Ar glovebox until use. The final dry thickness of the electrode was about 40 μm, with total mass 0.8 mg and dry mass ratios of 2:1:1, respectively, of graphite, carbon and PVDF binder.

### Optical cell preparation

A modified 2032 coin cell with an optically accessible glass window (170 μm thick, D263M glass) was assembled in an Ar-filled glovebox (H_2_O and O_2_ < 0.5 ppm). The optical coin cell was assembled using lithium metal counter electrode (15.6 × 0.25 mm thick, 99.9%, PI-KEM), glass fibre separator (16 mm diameter, GF/B, Whatman) wet with 120 μl of LP57/VC2 electrolyte (1 M LiPF_6_ in 3:7 wt ratio of ethylene carbonate and ethyl methyl carbonate with 2% wt of vinylene carbonate, PuriEL) and copper mesh (16 mm diameter, 99.99%, MTI) covering the self-standing electrode (Supplementary Fig. 31a).

The coin cell was mounted in a custom-made temperature-controlled holder (Supplementary Fig. 31b). The temperature of the holder was maintained using a PID controller (TED200C, Thorlabs) with a Peltier (TECF2S, Thorlabs) and temperature sensor (LM135Z, STMicroelectronics). The temperature of the coin cell was calibrated using a dummy PT100-sensor integrated optical coin cell under oil-immersion microscopy.

### Optical set-up

The optical microscopy was adapted from our previous set-up^22^. Briefly, the sample was epi-illuminated using an oil-immersion objective (×100, 1.45 numerical aperture, UPLXAPO, Olympus) by coupling a 4f (Kohler) illumination path from a fibre coupled LED (730 nm LED M730L5, M93L01, Thorlabs). The reflected light and scattered light were imaged using an sCMOS camera (ORCA-Flash4.0 v.3, Hamamatsu) with an overall magnification of ×167 and field of view of approximately 80 μm. The *z*-position of the sample was maintained within ±20 nm of the focal plane using the reflection profile of a 980 nm reference laser, as previously reported^22^. The final cell was mounted on a 3D piezo-controlled positioner (ECSx5050, AMC100, Attocube) with a custom-built stage and holder.

### Cycling and data acquisition

Galvanostatic sequences were applied using a potentiostat (Gamry Interface 1010E), at a current of 7.5 μA or about C/20 rate unless otherwise indicated. The cell was imaged at 25 °C during the full cycle (1 V–5 mV) and at 15 °C, 25 °C, 35 °C and 45 °C for later partial cycling (nominally 245–110 mV). For the experiments presented in the main text, images were acquired at a frame rate of 2 Hz with 8 ms integration time over a smaller acquisition window (about 500 × 450 pixels) using ImageJ^41^.

### Ex situ characterization

The half-cell was fully discharged to 1.5 V followed by a voltage hold for 2 h. The coin cell was then disassembled in an Ar-filled glove box, and the self-standing electrode was extracted and washed twice in 2 ml ethyl methyl carbonate (99.9%, Solvionic) and dried under vacuum. The sample was stored under ambient conditions before the ex situ studies. SEM imaging was conducted using a TESCAN MIRA3 scanning electron microscope at 5.0 kV, with a 7.78 mm working distance using the in-beam secondary electron detector. EBSD experiments were performed using a Thermo Fisher Scientific Helios 600i scanning electron microscope fitted with an Oxford Instruments Nordlys EBSD detector. The sample was mounted on a pre-tilted holder with additional stage tilt to achieve a total of 70°. Kikuchi patterns were recorded with an accelerating voltage of 20 kV and post-processed using Oxford Instruments AztecCrystal software. AFM images were obtained using an MFP-3D System (Asylum/Oxford Instruments), at 512 × 512 resolution, at 22.7 nm per pixel using a silicon probe (BudgetSensor, Tap300Al-G) in air. The AFM images were analysed using Gwyddion^42^.

### Image analysis

The initial image correction and data segmentation methods are found in Supplementary Information section 3.1. Videos created from differential images can be found in Supplementary Videos 1 and 2. Avalanche detection data processing and algorithm are described in Supplementary Information section 3.3. The detailed methodology of spatio-temporal correlation, including that of Fig. 4, is described in Supplementary Information section 3.2.

### Modelling

The RFIM formulation, Monte Carlo dynamics, parameters and simulation behaviour for 2D and 3D models can be found in Supplementary Information section 2, with example simulations shown in Supplementary Videos 3–5. Optical reflectivity modelling is described in Supplementary Information section 1.3.

## Online content

Any methods, additional references, Nature Portfolio reporting summaries, source data, extended data, supplementary information, acknowledgements, peer review information; details of author contributions and competing interests; and statements of data and code availability are available at 10.1038/s41586-026-10862-4.

## Supplementary information

Supplementary Information This file contains Supplementary Discussions, Supplementary Modelling, Supplementary Methods, Supplementary Figures and Supplementary References.

Peer Review File

Supplementary Video 1 Differential video of particle during cycling (from the main text). A differential video of the particle is shown in the main text. The dataset (at 2 fps) is from the 15 °C lithiation cycle 1 dataset, during the 1L–4L transition. A median filter of 5 frames (2.5 s) is applied to the time axis, and a moving window mean of 3 × 3 pixels is applied to the image. The differential video is calculated by dividing every (*n* + 3)th frame by the *n*th frame (1.5 s). Blue indicates an instantaneous drop and red indicates an instantaneous increase in intensity.

Supplementary Video 2 Differential video of particle during cycling (from Supplementary Fig. 7b). A differential video of a graphite particle (Hitachi MAGE3) during lithiation, as shown in Supplementary Fig. 7b,e. The dataset (at 2 fps) is taken from lithiation cycle 1, during the 1L–4L transition, at 23 °C. A median filter of five frames (2.5 s) is applied to the time axis, and a moving window mean of 3 × 3 pixels is applied to the image. The differential video is calculated by dividing every (*n* + 3)th frame by the *n*th frame (1.5 s). Blue indicates an instantaneous drop, and red indicates an instantaneous increase in intensity.

Supplementary Video 3 Simulation of lattice evolution in a 2D model without disorder (from Supplementary Fig. 17). A video of a 2D RFIM simulation during a single filling run, with zero disorder and thermal activation ($\mathop{h}\limits^{ \sim }$ = 0, $\mathop{T}\limits^{ \sim }$ = 0.5, equivalent to results shown in Supplementary Fig. 17). Yellow indicates filled, and purple indicates empty lattice sites (bottom). The 2D FFT of the lattice is shown (top).

Supplementary Video 4 Simulation of lattice evolution in a 2D model with disorder (from Supplementary Fig. 16). A video of a 2D RFIM simulation during a single filling run, with disorder ($\mathop{h}\limits^{ \sim }$ = 0.4, $\mathop{T}\limits^{ \sim }$ = 0.125, equivalent to results shown in Fig. 3 and Supplementary Fig. 16). Yellow indicates filled, and purple indicates empty lattice sites (bottom). The 2D FFT of the lattice is shown (top).

Supplementary Video 5 Simulation of lattice evolution in a 3D model with disorder, combined (from Supplementary Fig. 20). A video of a 3D RFIM simulation with disorder ($\mathop{T}\limits^{ \sim }$ = 0.1, $\mathop{h}\limits^{ \sim }$ = 0.9), of a single run during filling. This is equivalent to the results shown in Supplementary Fig. 20. The plot on the left shows the average filling curve, with a dashed vertical bar indicating the current position. On the right, four quadrants are labelled (a)–(d) clockwise: (a) the *XY* slice at *Z* = 12, where white indicates filled and black indicates empty lattice sites; (b) the *XZ *slice at *Y* = 4, where black indicates filled and the empty lattice sizes are coloured according to the *Z*-separation between filled sites; (c) *XY* view averaged across *Z* (32 layers) from empty (black) to approximately 0.5 (white) average fill density; and (d) the frame-by-frame differential calculated from (c), where blue indicates instantaneous filling and red indicates instantaneous emptying of one layer (±1/32 in fill density). See Supplementary Fig. 20d for a schematic.

## Data Availability

The data underlying the figures and supporting information are publicly available from the University of Cambridge Apollo repository at 10.17863/CAM.131676.

## Code availability

All codes used in this work are available from the corresponding authors upon request. The core code is available from the University of Cambridge Apollo repository at 10.17863/CAM.131676.
