# Supplementary material for: Avalanche-like intercalation and intraparticle correlations in graphite
Source: Nature. 2026 Jul 29;656(8127):364–9. doi: 10.1038/s41586-026-10862-4 (PMC13468132; doi:10.1038/s41586-026-10862-4)
Supplement: Supplementary file 1 [file 41586_2026_10862_MOESM1_ESM.pdf]

nature portfolio

https://doi.org/10.1038/s41586-026-10862-4

Supplementary information

Avalanche-like intercalation and intraparticle correlations in graphite

In the format provided by the authors and unedited

Supplementary Information

Avalanche-like intercalation and intraparticle correlations in graphite

Jiho Han[1,2,4], George S. Phillips[2,4], Alice J. Merryweather[1,2,4,5], Juhwan Lim[1,4], Christoph Schnedermann[1,4,5], Robert L. Jack[2,3], Clare P. Grey*[2,4], Akshay Rao*[1,4]

1. Cavendish Laboratory, University of Cambridge, J.J. Thomson Avenue, CB3 0US, Cambridge, UK. E-mail: ar525@cam.ac.uk

2. Yusuf Hamied Department of Chemistry, University of Cambridge, Lensfield Road, CB2 1EW, Cambridge, UK. E-mail: cpg27@cam.ac.uk

3. DAMTP, Centre for Mathematical Sciences, University of Cambridge, Wilberforce Road, Cambridge CB3 0WA, United Kingdom

4. The Faraday Institution, Quad One, Harwell Science and Innovation Campus, OX11 0RA, Didcot, UK

5. Illumion Ltd, Maxwell Centre, J.J. Thomson Avenue, CB3 0HE, Cambridge, UK

Corresponding author

Section 1: Supplementary Discussion

1.1. Disorder and additional characterisation

1.1.1. Particle characterisation

The cell was disassembled post-cycling and the particle was characterised ex-situ. The scanning electron microscopy (SEM) image shows the single particle of graphite partially imbedded in the electrode (Fig. S1a). From electron backscatter diffraction (EBSD) measurements, the inverse pole figure map (Fig. S1c) together with pole figures of the (0001) and (10-10) lattice vectors (Fig S1d, (i) and (ii) respectively) demonstrate that the particle is a continuous single crystal of graphite presenting the basal plane (0001) as opposed to polycrystalline, nanodomain graphite (e.g. MCMB, see Fig S8a). There are some subtle bends and kinks on the particle, better shown in the side-on image (Fig. S1b), which can be quantified and mapped by analysing the grain reference orientation deviation (GROD) in the EBSD data (Fig. S1f). This misorientation is likely due to elastic deformation, rather than due to grain boundaries between polycrystalline grains. In addition, the overall misorientation is relatively small (< 4°), and in most parts, evolves smoothly. We extract the GROD magnitude along a line (Fig. S1f black line) and plot its change in Fig. S1g. The equivalent line is drawn on the image of the clustered regions (Fig. S1e), and the boundaries between expected cluster regions is overlayed on Fig. S1g (dotted lines, and numbers above the plot). The areas of constant GROD slope shows excellent agreement with the expected cluster regions. A constant GROD slope indicates areas of constant elastic deformation, likely caused by bends and kinks in the particle.

To understand this further, we pursued atomical force microscopy (AFM) mapping of the particle. The AFM topology map (Fig. S1h) show the mostly smooth surface of the particle. Note that the scan line artifacts occur in the slow scan direction (x-direction), making the topology less reliable in the vertical direction. Similarly, an equivalent line is drawn on the topology map (Fig. S1h, black line) and the profile change along this is shown in Fig. S1i which clearly demonstrates the mostly flat particle surface separated bends and kinks. Again, the boundaries between expected cluster regions are indicated with vertical black lines. Overall, the bends and kinks are well correlated with the regions and the areas of constant GROD slope. For example the sharp kink at ~0.6 μm corresponds well to the sharp gradient between two areas of constant GROD slopes (Fig. S1g) near 0.6 – 0.8 μm, and the boundary between region 5 and 1 in Fig. S1e. Flake-type battery graphites are known to have such kinks, and examples of this in KS44 (studied in main text) and Hitachi MAGE3 graphite are shown in Fig. S8.

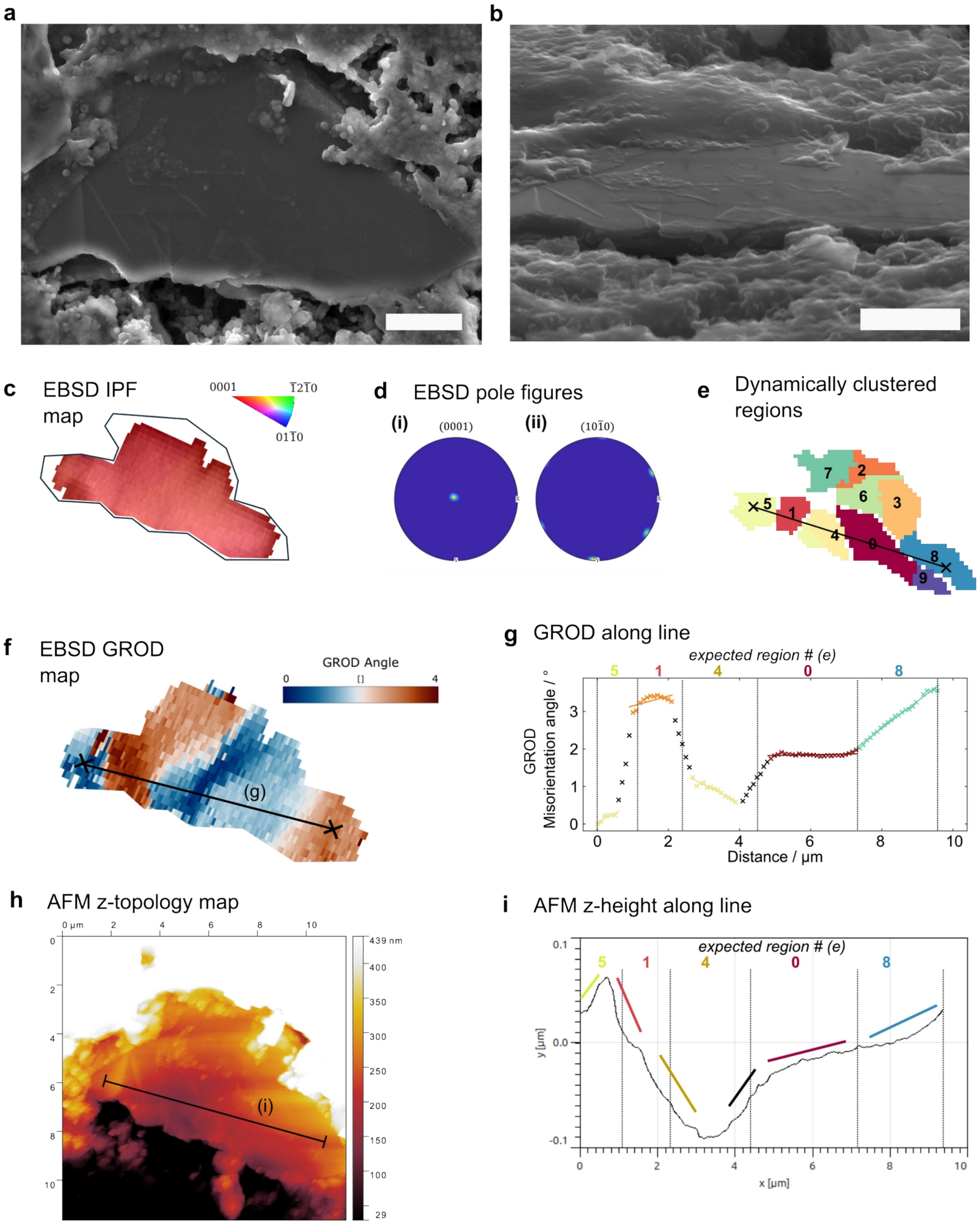

Figure S1 | EBSD, AFM and high-resolution SEM characterisation of graphite particle. a,**b,** Close up SEM images of the particle without tilt **(a)** and at 70° tilt **(b)**. Scale bar 2 µm. **c,** Electron backscatter diffraction (EBSD) map with inverse pole figure (IPF) color coding, measured from the ex-situ graphite particle in the electrode after cycling. The inverse pole figure (IPF) mapping colourcode is shown by the legend (top right). **d,** EBSD pole figures, integrated over the entire particle, for (0001) lattice vector **(i)**, and (10-10) lattice vector **(ii)**. **e,** Particle map showing clusters found through Spectral Coclustering method from the spatio-temporal correlation described in the main text Fig. 4. A white dotted line indicates the line of interest. **f,** The grain reference orientation deviation (GROD) map of the particle, showing the grain misorientation colourcoded as in legend (top right). A line of interest is drawn on top, in black. **g,** GROD misorientation value shown along the line of interest, with coloured numbers and vertical black dotted line indicating the location of expected cluster regions. **h,** Atomic force microscopy topology map of the particle imbedded in the electrode. The line of interest is drawn in black. **i,** The z-height profile along line of interest, with coloured numbers and vertical black dotted line indicating the location of expected cluster regions.

1.1.2. Disorder correlation with clustered regions

The ex-situ characterisation strongly suggests that disorder in the particle may originate from elastic deformation due to bends and kinks. This suggest that the clustered regions during intercalation (at least partially) originate from the kink boundaries and ripplocations disorder(Badr & Barsoum, 2023).
In fact, this suggests that the poor connectivity of clustered regions in the left side of the particle, identified via correlation methods (main text Fig. 4b), may be related to higher density of kinks and elastic disorder on the left side of the particle. In Fig, S2 we show the local gradient norm on the particle calculated from the AFM z-topology map. This better highlights areas of constant curvature and location of kinks. Ignoring the horizontal scan line artifacts, the left side of the particle has more kinks and bends whereas the lower right side is flatter and continuous. This is in agreement to the connectivity correlation intensity sums (main text Fig. 4b) as well as the temporal distribution of avalanches (Supplementary Fig. S32), indicating that poor intraparticle connectivity and broadened temporal spread of avalanches occur in regions with higher elastic deformation.

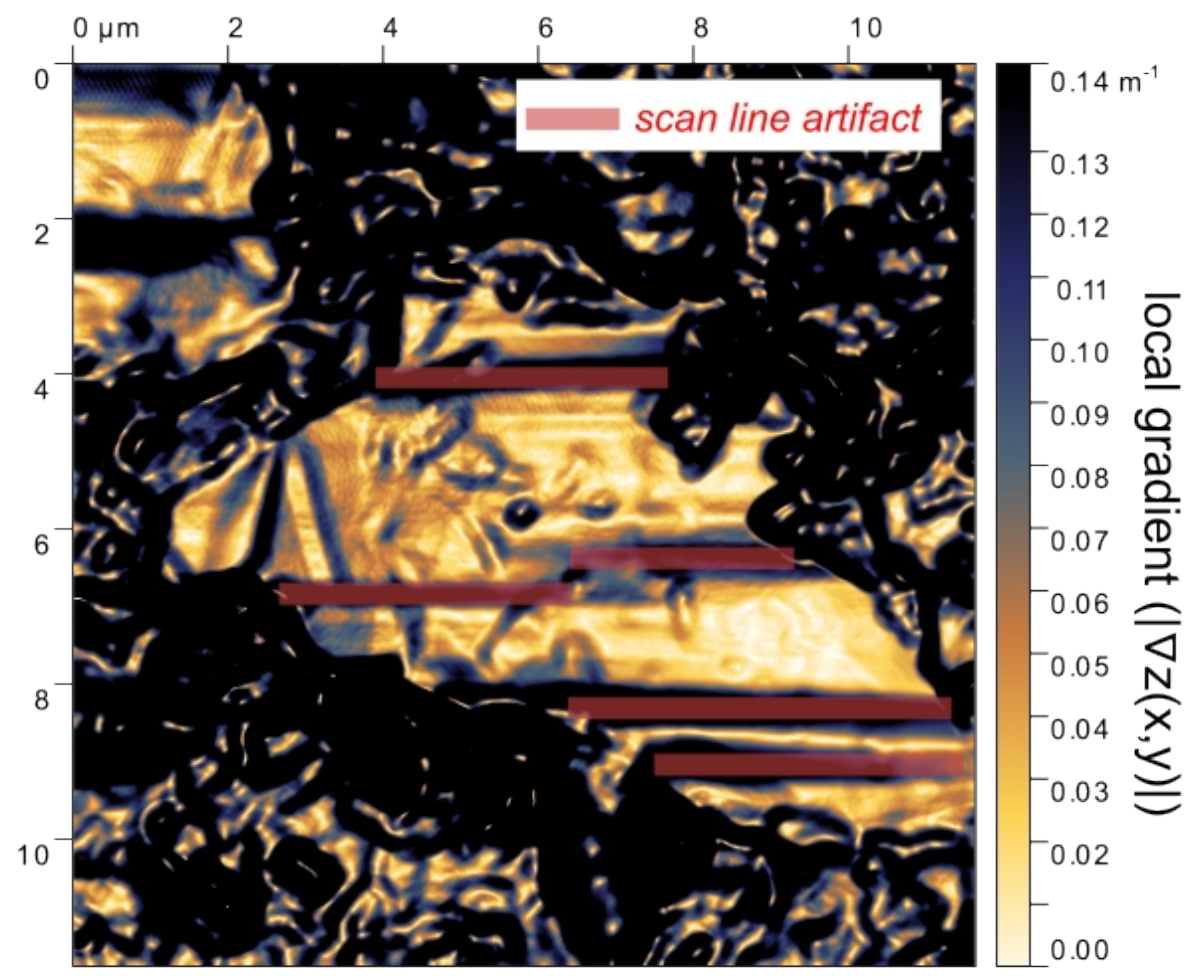

Figure S2 | Local gradient norm from AFM map. The local gradient, defined as the norm of the 2D gradient vector at each point is calculated. To account for surface roughness, a running window mean of 3 x 3 pixels (68 x 68 nm) is applied. Sharp gradients along exactly horizontal lines arise from scan line artifacts, and have been marked with a red square.

1.2. Avalanches

1.2.1. Signatures of avalanches

When a system with collective interactions, quenched disorder and near-athermal dynamics is driven slowly, it produces crackling noise or avalanches - intermittent bursts of activity due to jumps between locally stable configurations in a rough energy landscape (Baró et al., 2016; Sethna et al., 2001). When the system disorder is tuned (or self-organised) to near criticality, there will be a scale-invariant power law in avalanche size and length distribution (Perković et al., 1995). Beyond crackling noise, these system display hysteresis arising from disorder(Vives & Planes, 1994), memory between cycles(Sethna et al., 1993) , and a complex spatial distribution of avalanches (Kuntz et al., 1999; Walsh et al., 1998). It is important to note that the intermittent steps still appear and are referred to as avalanches when the system is not perfectly athermal(Baró et al., 2016; Yao & Jack, 2023), not driven quasistatically (White & Dahmen, 2003), and with disorder not equal to critical disorder(Perković et al., 1995).

The transition during graphite intercalation in the dilute stages shares the following features with these systems. The step sizes are broadly distributed, following a rough power law (Fig. S3a). The step-size distributions are tuned to the intra-particle disorder (Section 1.1, main text Extended Data Fig. 2) and sample

disorder (Section 1.2.3). The intermittent steps are dependent on the driving rate (Section 1.2.2), and the individual steps do not occur randomly over different cycles, retaining similarities in their temporal distribution (Fig. S25).

Finally, we stress that “region” labelling of the particle does not imply that the steps occur exclusively in the region. Shown in Fig. S4, we show the spatial extent of avalanches detected specifically in “region B”. The spatial extent vary broadly in spatial structure, from one small cluster within the region to the entire particle, much like the avalanche clusters which appear in RFIM near criticality. This implies that, although the collective behaviour of lithium intercalation is strongly affected by the existing spatial structure of disorder (such as shown in Supplementary Section 1.1), the steps cannot be solely attributed to kink boundaries nor do steps occur exclusively within one region confined by the kink boundaries. In fact, we have observed avalanche-like steps without the appearance of persistent spatial regions, where each avalanche cluster was more randomly distributed spatially. For example in smaller particles (Supplementary Video S2) or on a relatively kink-free exfoliated graphite flake (such as that shown in Supplementary Fig. S7a).

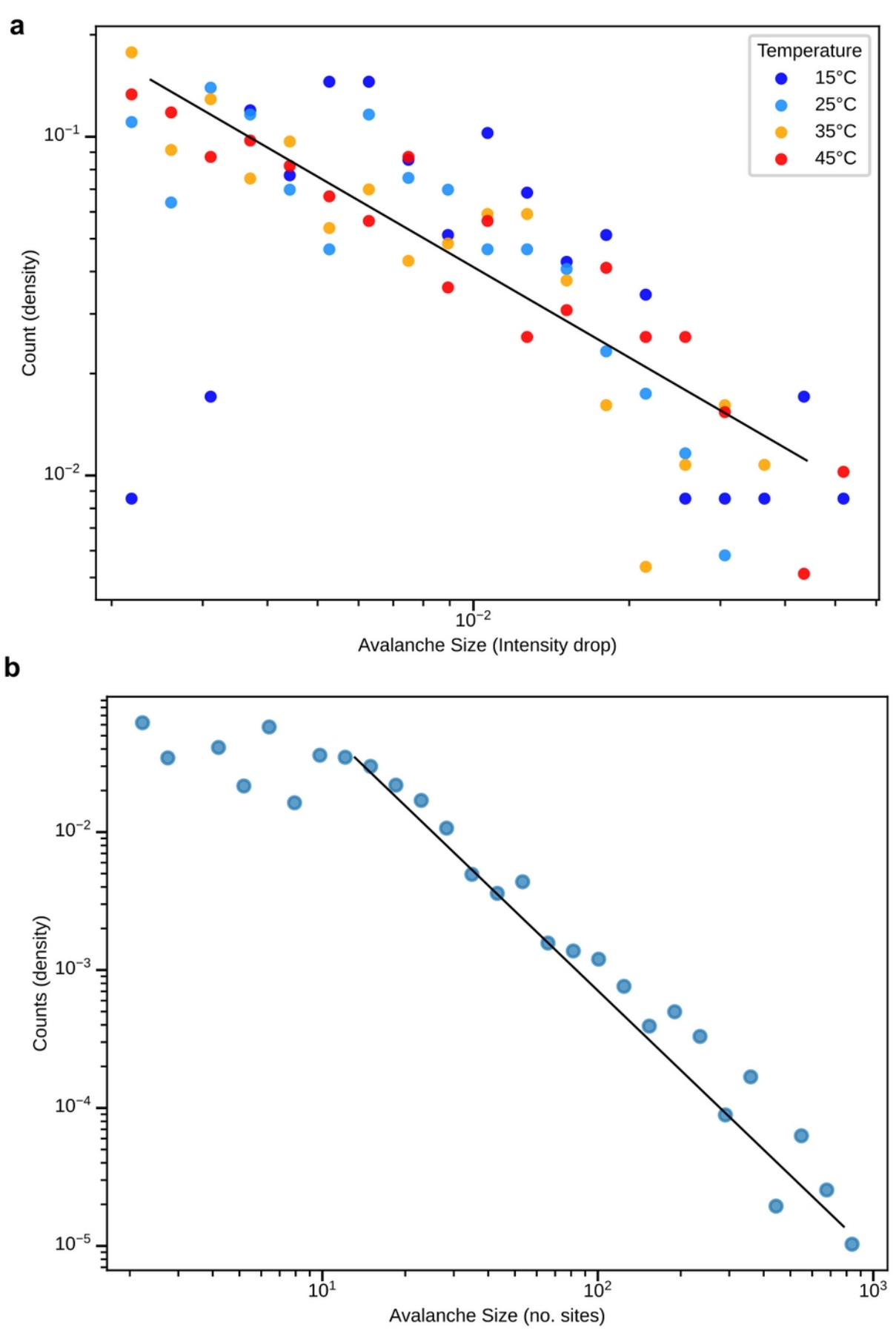

Figure S3 | Avalanche size distribution curves. A log-log histogram of avalanche size distribution is made for the experiment (**a**) and simulation (**b**). The experimental avalanche size is defined as the drop in intensity, with the intensity normalised to start of lithiation. The simulation avalanche size is defined as number of sites filled. Statistics are collected from region A, B and C during the 1'L - 4L transition during de/lithiation in cycle 1 and 2. The model statistics are collected during the 1'L - 4L transition from a single run in the 3D model (lattice size 128 x 128 x 64, beta = 10, h = 1, steps = 500,000), but similar power law statistics arise from the 2D model ($\tilde{h} \sim 0.4$) as well.

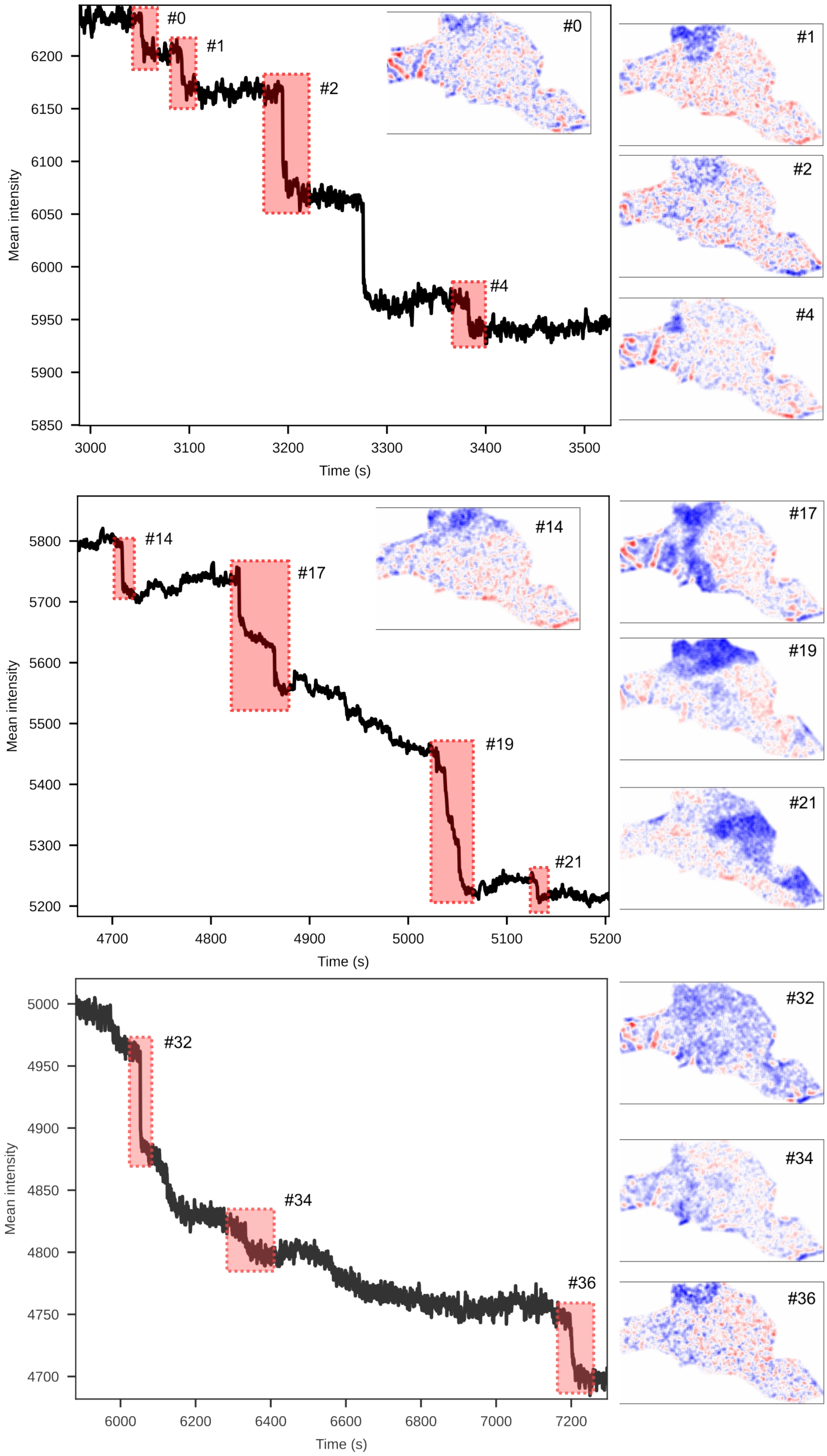

Figure S4 | Spatial distribution of each avalanche event. Each detected avalanche event is logged in the start and end time, and a differential image of the particle between these time points is constructed. A few of these differential images (**right**) is shown alongside the intensity trace (**left**), with the location of the avalanche events highlighted in the red boxes and labelled with numbers. The intensity trace and avalanche events are from Region B (from main text Fig. 1f) 15°C, cycle 1.

1.2.2. Avalanche dynamics at different C-rates

An electrode with the same composition as studied in the main text of the paper was cycled at different C-rates at 23 °C, following a C/20 formation cycle. The mean intensity from a small single particle, and corresponding differential intensity is shown in Fig. S5, for C/100, C/20, C/2 and 1C. The intermittent intensity steps were most clearly observed for slower rates (C/100, C/20), with a clear, well separated sequence of steps of various sizes throughout de/lithiation. This is better illustrated in the lower panel, showing the sequence of sharp pulses in the differential intensity plot. At higher rates, the steps became blurred due to the overlap of avalanches resulting in a longer, continuous sequence. This is clearly seen in Fig. S6, where the experiments at C/100 and 1C are compared. The total number of frames per half-cycle is kept similar (~100k frames), such that we are not limited by our temporal resolution, and a similar region (1L-4L transition, between 1.0 to 0.8 mean intensity) is shown. The well separated, sharp steps at C/100 results in correspondingly well separated and large peaks in the differential. At 1C, there is a tendency for a longer, continuous-like drop in intensity. This is consistent with findings from other avalanching systems, where at higher driving rate, the avalanches merge and combine, resulting in larger and longer pulses (Durin & Zapperi, 2004; White & Dahmen, 2003).

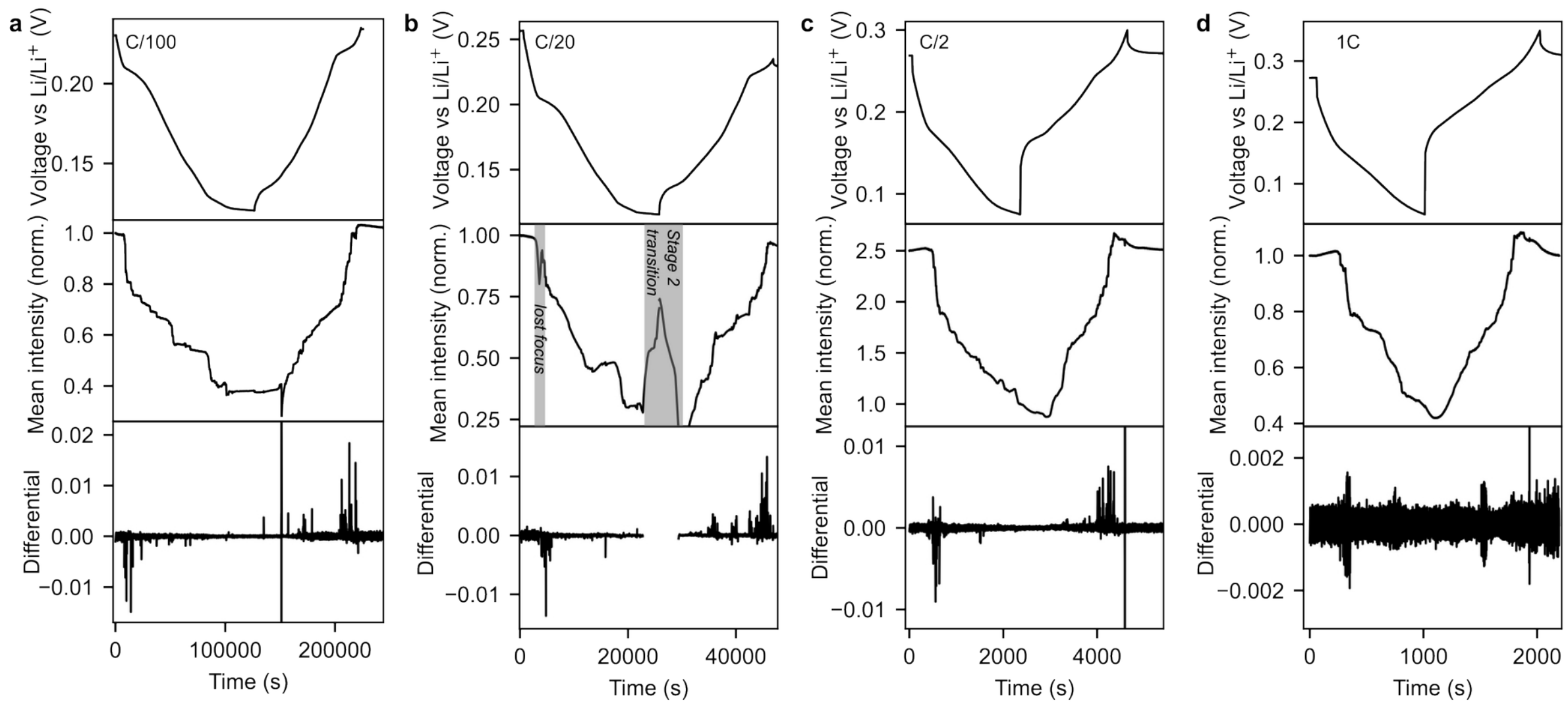

Figure S5 | Effect of C-rate. An optical coin cell, with lithium metal counter and same graphite electrode composition as studied in the main text (KS44:PVDF:super-p = 50:25:25, 80 µm self standing) is cycled with imaging at C/100 with 2 fps acquisition **(a)**, C/20 with 2 fps acquisition **(b)**, C/2 at 20 fps acquisition **(c)** and 1C at 100 fps acquisition **(d)**. The upper panels show the corresponding cell voltage during cycling. The middle panels show the corresponding mean intensity extracted from a small single particle during cycling, normalised to start of lithiation. The bottom panel show the raw mean intensity differential signal during cycling, calculated after applying a median filter (kernel = 15) to the intensity data.

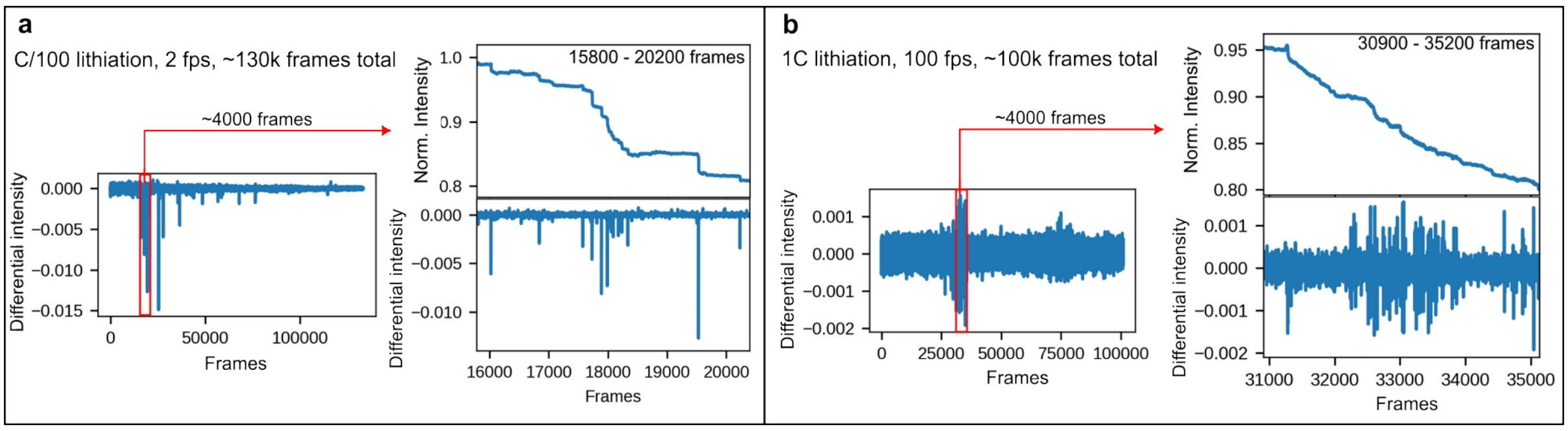

Figure S6 | Detailed comparison between C/100 and 1C. The single particle intensity data is compared between C/100 **(a)** and 1C **(b)** rates. The image is acquired much faster for 1C rate, and the total number of frames per half-cycle is kept approximately the same for both rates (~100k frames). A close up of ~4000 frames are selected during the 1L-4L transition, and the raw normalised intensity and the differential intensity are shown.

1.2.3. Disorder and sample dependence

In addition to disorder present within a single particle, as discussed in the main text, to understand the effect of graphite-type on the step-like intercalation behaviour, we have repeated the experiment with different graphite samples. We compare MCMB1028 graphite, Hitachi MAGE3 commercial battery graphite and exfoliated Kish graphite, in order from most to least disordered. Both MCMB1028 and MAGE3 were characterised in electrode, whereas the Kish graphite was first exfoliated on glass, with a graphite electrode (1:1:1 = KS44 graphite : porous carbon : PVDF) cast on top in order to observe a constant-current behaviour with a consistent rate. All samples were run at C/20, 23 °C after a formation cycle (C/20) in an optical coin cell (Figure S7). Typical SEM images from corresponding samples can be found in Figure S8.

All samples showed some intermittent, step-like behaviour, which is particularly clear in the differential intensity plots (Fig. S7d-f lower panels), however, the steps were much smaller and harder to resolve in the disordered, MCMB1028 sample (see zoomed inset, Fig. S7f middle panel), resulting in the smallest peaks in the differential plots. In the least disordered Kish graphite, much more clearly separated, sharp steps are seen, resulting in larger peaks in the differential. This is highly consistent with theoretical avalanche dynamics (Perković et al., 1999; Sethna et al., 1993), where disorder beyond the critical disorder results in larger avalanches being cut-off, and more frequent smaller avalanches overall.

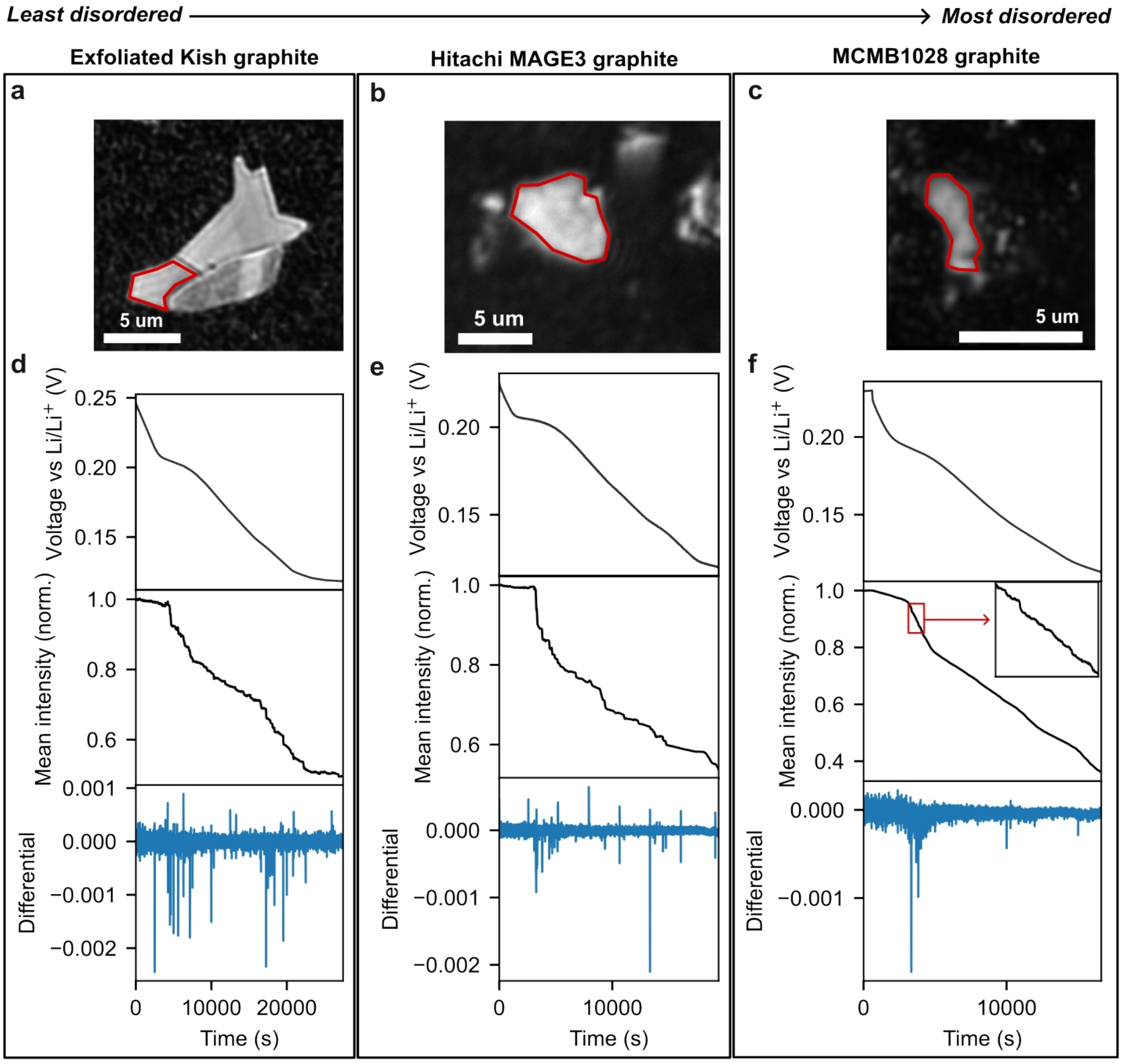

Figure S7 | Step intensity behaviour in other graphite types. a, Optical image of Kish graphite exfoliated onto glass, electrically connected with a porous electrode slurry (33:33:33 = KS44 graphite:porous carbon:PVDF). The bulk region of the exfoliated particle (indicated in red) was used for the mean intensity calculations. **b,** Optical image of single particle graphite embedded in electrode (MCMB1028, LP57/FC2 electrolyte using 40:40:20 = AM:porous carbon:PVDF electrode and lithium metal counter electrode). **c,** Optical image of single particle graphite embedded in electrode (Hitachi MAGE3, LP57/FC2 electrolyte using 40:40:20 = AM:porous carbon:PVDF electrode and lithium metal counter electrode). Region indicated by red line was used for mean intensity calculations. **d,e,f,** Cell voltage vs Lithium metal counter electrode (top panel), mean intensity (middle panel), and differential intensity (bottom panel) plotted against time. Mean intensity has been normalised to the start of cycle. Differential intensity is calculated after applying a median filter (kernel = 15) to the mean intensity. Red square ((f), middle panel) illustrates the location of the zoomed inset, which highlights the step-like features in the MCMB1028 graphite intensity plot.

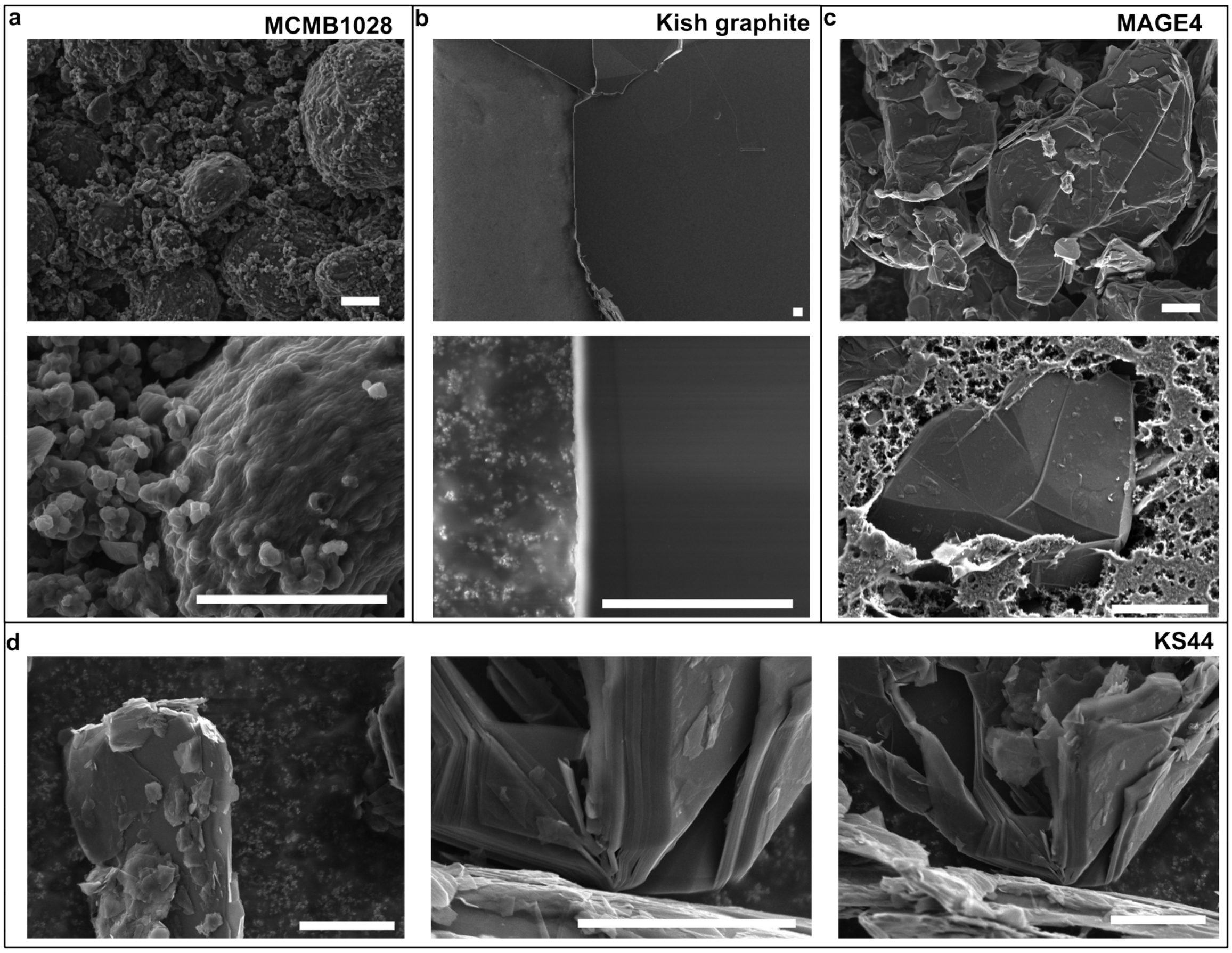

Figure S8 | SEM images of different graphite samples, highlighting kinks and bends. SEM images showcasing typical sample morphology of MCMB1028 **(a)**, Kish graphite **(b)**, Hitachi MAGE3 **(c)** and Timcal Timrex KS44 **(d)**. All images measured on the original powder samples, except for (**c**) lower panel, which shows a typical graphite flake embedded in an electrode. All scale bars, 2 um.

1.3. Optical modelling

1.3.1. Optical response of lithiated graphite

The optical reflectivity of graphite intercalation compounds (GICs) can be described by the Drude free carrier model(Dresselhaus & Dresselhaus, 2002). Briefly, in the Drude model, the dielectric function is approximated by its core dielectric with its free carrier contribution:

$$\varepsilon(\omega) = \varepsilon_\infty - \frac{\omega_p^2}{\omega^2 + i\gamma\omega},$$

Where ε_∞ is the core dielectric, γ is the damping constant and the ω_p is the plasma frequency, given by

$$\omega_p = \sqrt{\frac{Ne^2}{\varepsilon_0 m^*}},$$

where m^* is the carrier effective mass, ϵ_0 is the vacuum permittivity and N is the carrier density. We assume that lithiation results in a linear increase in carrier density with full ionisation, with lithiation x = 1 (LiC_6) for Stage 1 GIC, x = 0.5 (LiC_{12}) for Stage 2 GIC, and x = 0.22 (LiC_{27}) at the end of dilute stages or "Stage 2L" GIC (Ohzuku et al., 1993). Parameters used in the model can be found in Table S1.

As shown in Figure S9a for a wavelength of 700 nm, the Drude model predicts an initial near linear decrease in intensity during lithiation for the dilute stages, followed by an increase in intensity at the beginning of the dense stages. At high lithiation levels, the intensity change becomes less pronounced. This behaviour can be explained by the increase in carrier density for GICs changing plasma frequency. As the carrier density of graphite increases during lithiation, the plasma frequency shifts to lower wavelengths (higher energy) and eventually crosses the wavelength of the experiment. The Drude model predicts metallic reflections (i.e. reduced reflection) for wavelengths below the plasma frequency, which results in a non-monotonic intensity trend at 700 nm over the course of lithiation. This can be seen clearly by evaluating the spectral reflectivity profile shown in Figure S9b, which further illustrates this behaviour and connects the observation to the well-documented colours of Li-GICs, namely Stage 1 GIC (bright yellow), Stage 2 GIC (red), dilute stage GIC (grey-blue) as seen in previous studies(Y. Chen et al., 2021).

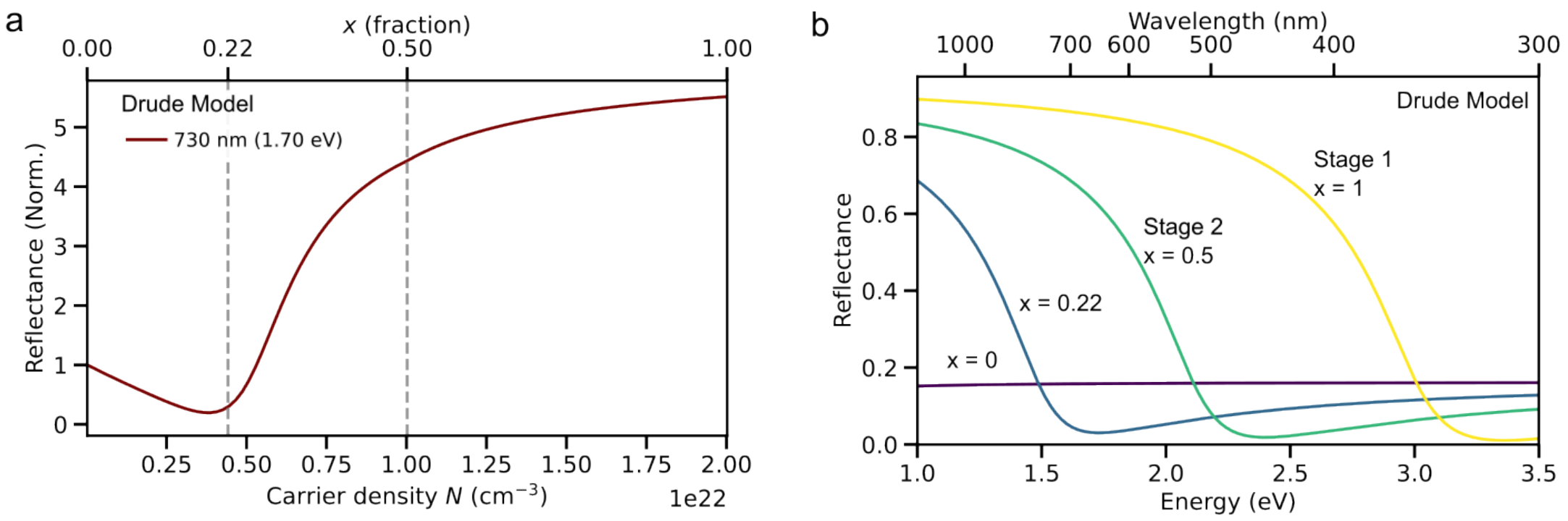

Figure S9 | Drude model of graphite reflectivity. a, Normalised reflectance of lithiated graphite at single wavelength (730 nm) according to the Drude model, as a function of lithiation (top axis) and resultant carrier density (bottom axis). Dotted vertical lines correspond to 22% lithiation (end of dilute stages) and 50% lithiation (Stage 2). **b,** The reflectance spectra vs wavelength, at various lithiation states according to the Drude model. x = 0, 0.22, 0.5, and 1 are selected for comparison.

Table S1 | Drude Model parameters

ε_∞	m^* (m_e)	N (cm^{-3})	γ (rad/s)
5.5	0.04 (graphite), 0.5 (Stage 1) (Dresselhaus & Dresselhaus, 2002; Williamson et al., 1965)	2×10^{19} (graphite), 2×10^{22} (Stage 1) (Dresselhaus & Dresselhaus, 2002)	5×10^{14}

1.3.2. Surface vs bulk intensity contributions

At λ = 730 nm, the penetration depth ($d_p = \lambda/4\pi k$) for pristine graphite is ~45 nm, or ~140 layers (Song et al., 2018). At the end of the dilute stages (x = 0.22), the penetration depth slightly decreases to ~40 nm, or ~120 layers. The graphite used in this work (Timcal Timrex KS44) has a has a reported c-axis crystallite size (L_c) of >100 nm. Since the domain size of the graphite in the c-axis is similar to the penetration depth of our technique, our experimental observations can be attributed to that of the bulk graphite response. Critically, by probing more than 100 layers we ensure that we (1) fully describe the dynamics of step-like intercalation and (2) overcome the concern that the step-like intercalation process is only a surface effect.

1.4. Additional Electrochemistry

To better understand the electrochemistry under very slow C-rates, a normal coin cell with a graphite electrode of the same composition as that studied in the main text was cycled at C/100, C/20 and C/2 in a potential range approximately corresponding to the dilute stages. A dQ/dV plot calculated from this is shown in Fig. S10, where the C/100 rate shows a reduced 1L-4L hysteresis compared to C/20, which further reduces with increasing temperature. The hysteresis values for the 1L-4L calculated from the cell voltage and single particle measurements (*) can be found in Table S2. The dQ/dV at C/20 and C/100 shows very small peaks (denoted 2a and 2b in Fig. 2 in the main text), which is highlighted in Figure S11. An interpretation of this is discussed in Supplementary Section 2.3.

Overall, the voltage hysteresis observed in the cell and in the single particle intensity can be divided into (1) mass transport and particle-electrode connectivity limitations, (2) reaction overpotential and (3) an intrinsic zero-current hysteresis arising due to non-equilibrium, athermal phase transition. In Fig. S12, the single particle intensity (averaged over entire particle), is plotted against cell capacity. At 25°C and higher, it shows very good overlap during lithiation and delithiation, demonstrating that the particle does not lag behind the electrode and little mass transport overpotential exists, except at 15°C.

The reaction overpotential and intrinsic zero-current hysteresis cannot be separated, however we have tabulated the approximate 1L-4L voltage hysteresis from the cell potential at different rates, and show that at C/100 at 45°C there exists ~9.8 mV of voltage hysteresis. This is the same as the zero-current voltage hysteresis reported previously(Mercer et al., 2021), and from this it is reasonable to assume that the voltage hysteresis in single particle intensity measurements presented in the main text is mostly composed of a combination of reaction overpotential and intrinsic hysteresis.

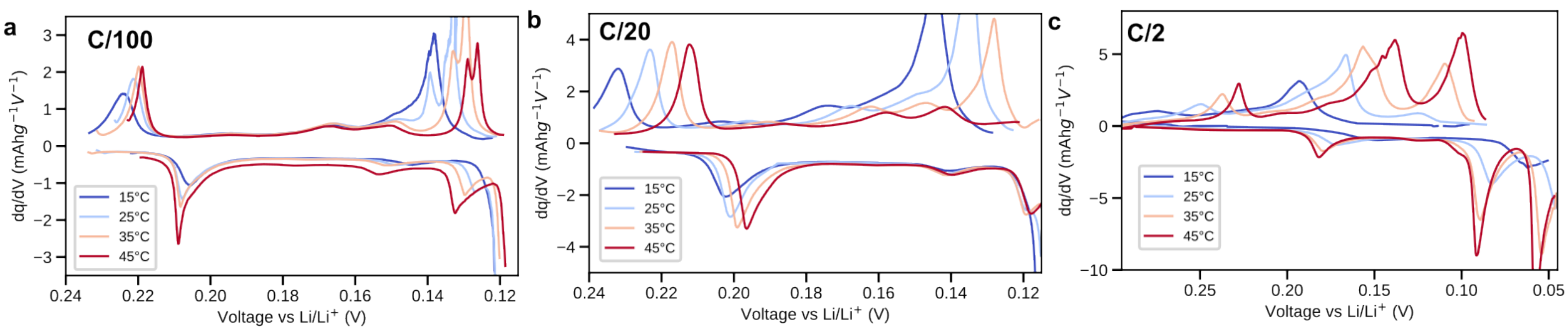

Figure S10 | C-rate and Temperature dependent dQ/dV. The differential capacity (dQ/dV) plots are calculated from a constant current de/lithiation at **a,** C/100, **b,** C/20, **c,** C/2. The voltage range is limited between ~250 mV and ~115 mV to only cycle in the dilute stages for C/100 and C/20. Note that at C/2 we lithiate beyond the dilute stages at 25°C and higher, where the two dQ/dV peaks at the lowest voltage corresponds to the two dense stage transitions. A normal coin cell with lithium metal counter and a graphite electrode of the same composition as that studied in the main text (KS44:PVDF:super-p = 50:25:25, 80 μm self standing) is used, with a custom built heated cell holder.

Table S2 | 1L-4L voltage hysteresis measured from cell potential during constant current de/lithiation at various C-rates (Fig. S10). **single particle 1L-4L voltage hysteresis, measured from mean intensity over particle plotted vs cell voltage.*

	**C/2**	**C/20**	**C/100**
**15°C**	120 mV	30 mV (*41 mV**)	18 mV
**25°C**	79 mV (*84 mV**)	23 mV (*25 mV**)	13.2 mV (*14 mV**)
**35°C**	58 mV	18 mV (*20 mV**)	11 mV
**45°C**	46 mV	16 mV (*18 mV**)	9.8 mV

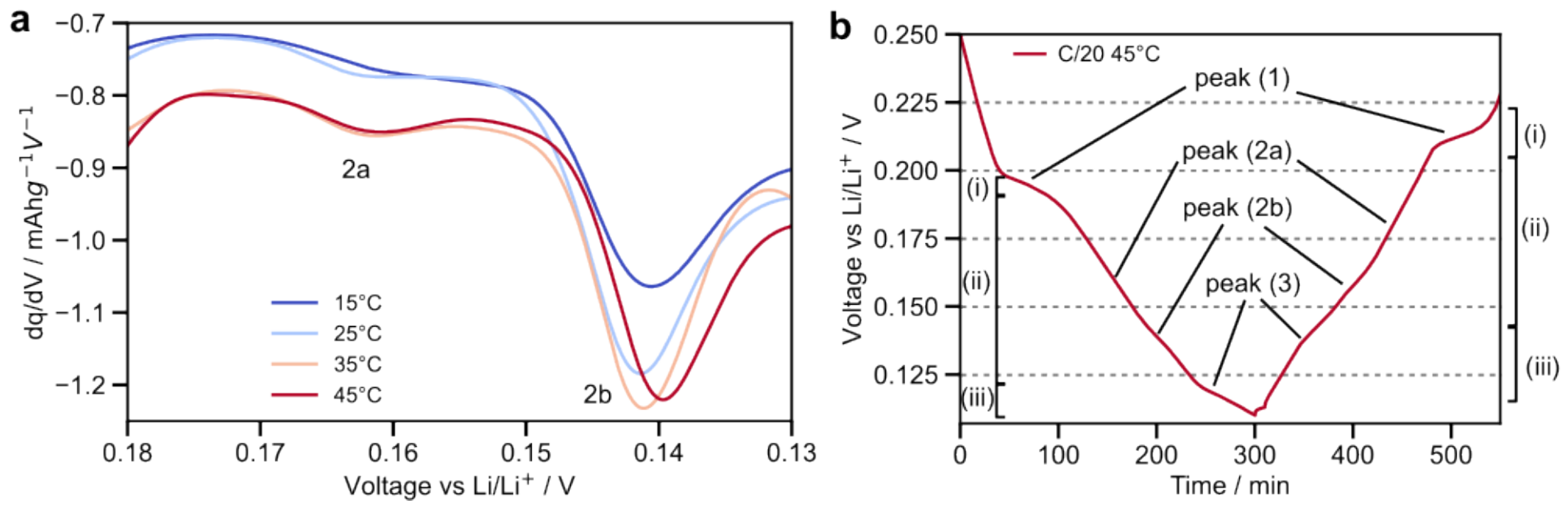

Figure S11 | Detailed C/20 dQ/dV peaks and voltage profile. a, Differential capacity plot (dQ/dV) shown in main text Fig. 2b, zoomed in to show peak 2a (left) and 2b (right) during lithiation. **b,**Voltage curve during C/20 partial cycling at 45 °C, from which the differential capacity is calculated. dQ/dV peak locations have been marked (1)-(3) and the approximate voltages corresponding to regime (i)-(iii) according to main text Fig. 2a have been marked.

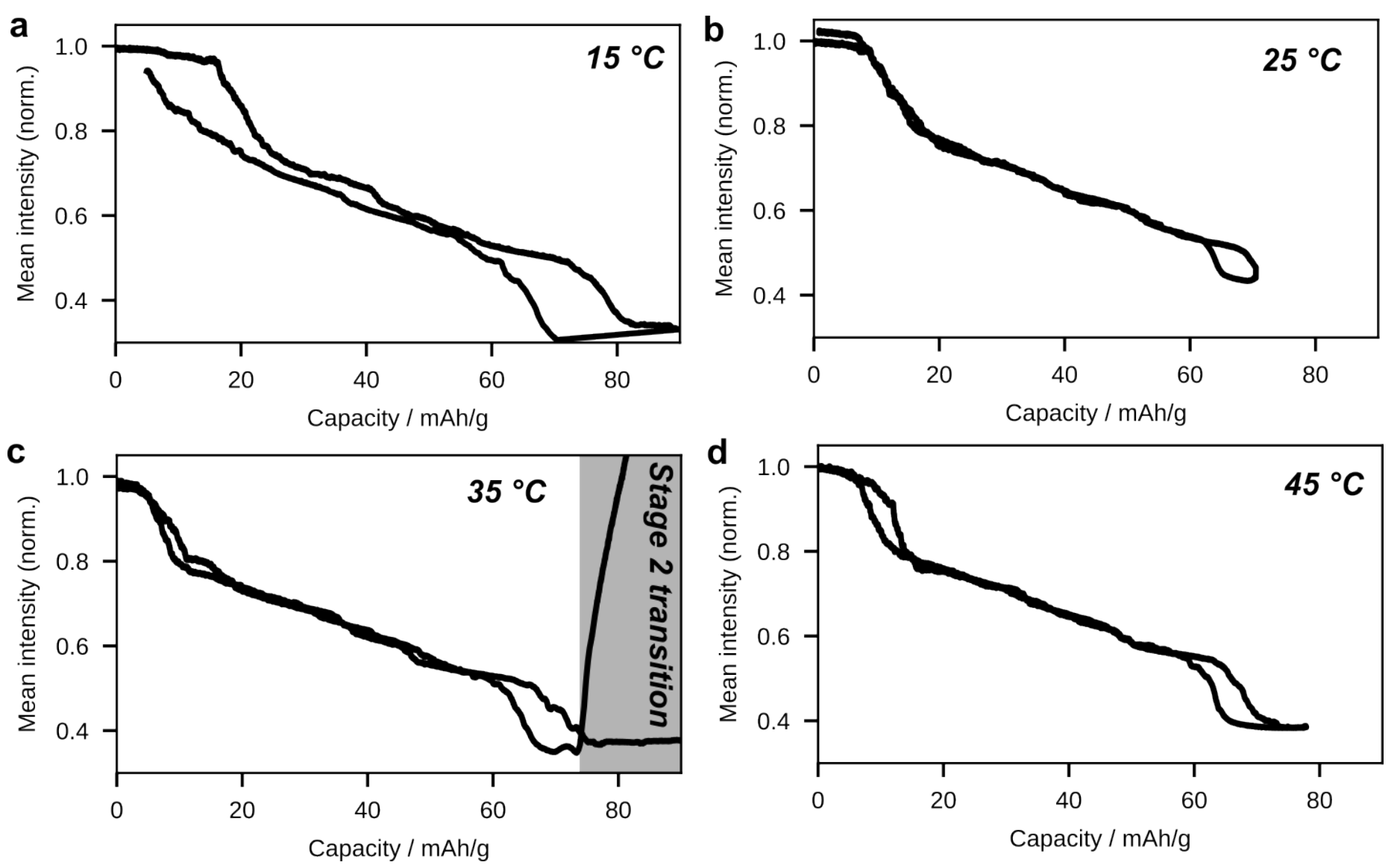

Figure S12 | Particle mean intensity vs capacity. Average particle intensity is plotted against cell capacity, from same dataset shown in Figure 2a of the main text. Data from cycle 1, at 15 °C, 25 °C, 35 °C, 45 °C, in (**a**) – (**d**) respectively.

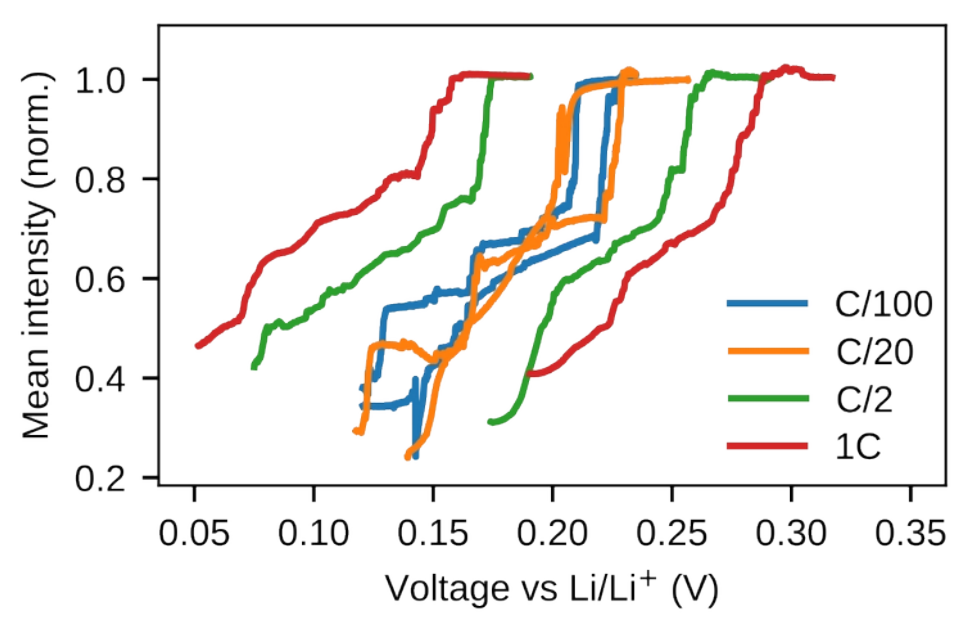

Figure S13 | Mean intensity vs Cell voltage at different rates. The mean intensity from a small single particle is shown against interpolated cell voltage. Same dataset as shown in Figure S5-6.

Section 2: Supplementary Modelling

2.1. Modified 2D random field Ising model

Previous studies on graphite lithiation have often used multilayer coupled phase field modelling (Chandesris et al., 2019; Smith et al., 2017), which can accurately capture the kinetic behaviour of a diffusive, phase separating system, but struggle with the incorporation of long range repulsive interlayer coupling needed to observe higher order stages(Schon et al., 1988) and disorder, which is known to play an important role in graphite (Dahn et al., 1990; Zheng et al., 1995) and phase-transition dynamics(T. Chen et al., 2023) .

We adopt a minimal model that incorporates the interplay between intra and interlayer coupling, disorder, and temperature. These features lead to avalanche-like phenomena during the filling. We consider a lattice of $L_x \times L_y$ sites; the site at position (x, y) can be either filled with lithium ($s_{x,y} = 1$) or empty ($s_{x,y} = -1$). Hence the filling fraction is given by:

$$\phi = \frac{1}{2L_x L_y} \sum_{x,y} (s_{xy} + 1)$$

The dynamics involves individual spins changing their state, which corresponds to Li ions filling or emptying a region within a single gallery in the system. Hence our model does not capture kinetics of Li diffusion, which we assume is not rate-limiting during de/lithiation in the slow constant current (C/20) experiment. The same assumption allows us to mimic the galvanostatic (constant current) experiment by a ramped potential (formally equivalent to cyclic voltammetry), see below. We take periodic boundary conditions, in order to minimize finite-size effects in the simulations.

The interactions of the Li atoms are modelled as a random-field Ising model with attractive intralayer (x) coupling up to nearest neighbour, and repulsive interlayer (y) coupling up to third nearest neighbour. The second and third neighbour coupling were assumed to be completely screened as per previous models(Chandesris et al., 2019). The disordered structure of the galleries is modelled in an idealized way by assigning random fields independently to each site ($h_{x,y}$), drawn from a Gaussian distribution with mean zero and standard deviation σ. The resulting energy is

$$E = -J \sum_{x,y} s_{x,y} s_{x+1,y} + K_1 \sum_{x,y} s_{x,y} s_{x,y+1} + K_2 \sum_{x,y} s_{x,y} \frac{1 - s_{x,y+1}}{2} s_{x,y+2} + K_3 \sum_{x,y} s_{x,y} \frac{1 - s_{x,y+1}}{2} \frac{1 - s_{x,y+2}}{2} s_{x,y+3} - \sum_{x,y} (\mu + h_{x,y}) s_{x,y}$$

where J is the intralayer coupling constant, K1, K2, K3 are interlayer coupling constants (see Table S2); also μ is the potential and $h_{x,y}$ is the random field on site (x, y). (The factors of $(1 - s_{x,y+1})/2$ etc incorporate the screening between the layers.)

The model dynamics proceeds by Monte Carlo moves in which a site $(\mathrm{x,y})$ is selected at random; we propose to change its state from $s_{x,y}$ to $-s_{x,y}$ and this proposal is accepted with probability $\min[1, e^{-\Delta E(x,y)/(k_B T)}]$ where T is the temperature, k_B is Boltzmann's constant, and $\Delta E(x, y)$ is the difference in energy before and after the proposed change. A Monte Carlo sweep corresponds to $L_x \times L_y$ proposed moves so that one proposes to flip each spin (ie fill/empty the lattice site) on average once per sweep. The mimic quasi-static filling, we ramp the potential μ slowly from μ_{init} to μ_{final} over a total simulation time of N_{MC} Monte Carlo steps (mcs).

We adopt dimensionless variables in which energies are measured relative to the intralayer coupling J. Since the energy only enters the dynamics via the dimensionless quantity $\Delta E(x,y)/(k_\mathrm{B}T)$, the dependence of the simulation results on the model parameters is fully determined the following set of dimensionless parameters:

$$k_\mathrm{B}T/J, \quad \sigma/J, \quad K_1/J, \quad K_2/J, \quad K_3/J, \quad \mu_\mathrm{init}/J, \quad \mu_\mathrm{final}/J, \quad N_\mathrm{MC}, \quad L_x, L_y$$

For simplicity, we fix the values of the last 8 parameters to the values given in Table S2. Note that the interlayer couplings decrease proportional to the squared distance between the layers; the values of μ_init and μ_final are chosen such that the minimal energy state at the initial and final time have approximately zero-filling and half-filling respectively.

We vary the remaining parameters which are: $\tilde{T} = (k_\mathrm{B}T/J)$, measuring the size of thermal fluctuations relative to intralayer attraction; and $\tilde{h} = \sigma/J$ which measures the strength of the disorder, relative to intralayer interactions. We explore the model behaviour as a function of these parameters, shown in Figure S14. At low temperature and disorder strengths, instead of local interaction between partially filled rows, the entire row fill at once, as evidenced by the large steps in the hysteresis curves (red squares in Fig. S14). This was a result of the increasing correlation length in the x-direction (intralayer) beyond the lattice size, and alleviated by running the simulation with a larger cell size.

We emphasize that this is a minimal model that captures the interplay of disorder, temperature, and anisotropic interactions. We expect its behaviour to be robust in that more detailed modelling of interactions or disorder implementation would change the quantitative behaviour, but the trends and qualitative features would be preserved.

The initial simulations were run using parameters which were later converted to dimensionless parameters for the model description in the main text, as they better quantify the relative significance of each parameter. The conversion does not affect model simulation results. These parameters and conversion can be found below in Table S3.

Table S3 | 2D Model parameters

	J	*K1*	*K2*	*K3*	µ range	*Total mcs steps*	*Lattice size*
**Simulation params.**	0.4	0.5	0.125	0.056	-2.0, 0.5	80000	200 (x), 80 (z)
**Dimensionless equiv.**	1	1.25	0.3125	0.14	-5.0, +1.25	80000	200 (x), 80 (z)

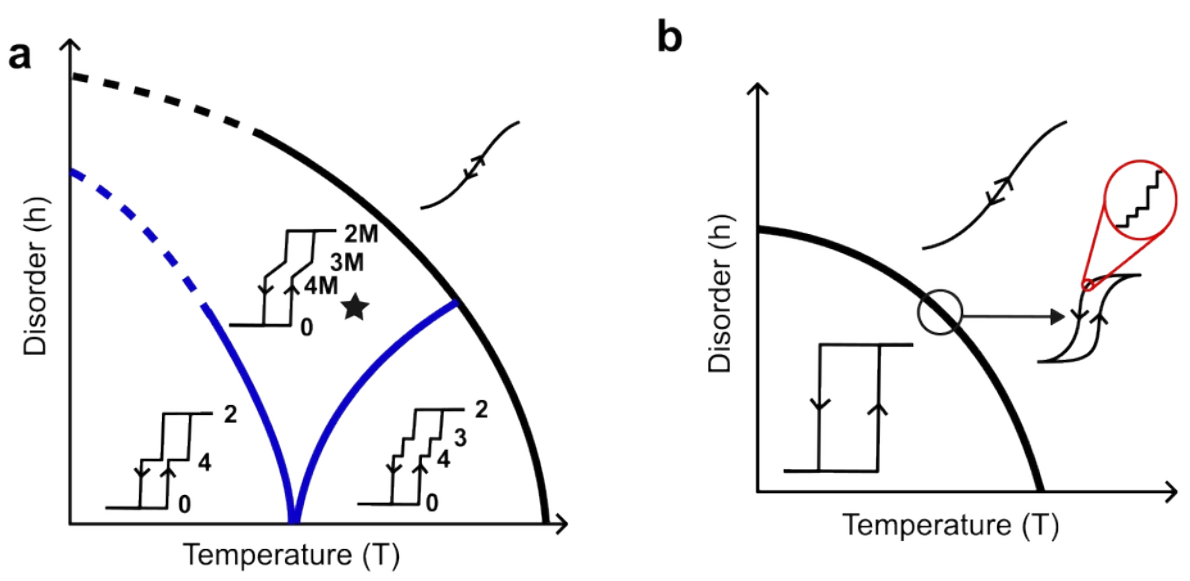

Figure S14 | Random field Ising model and trends. a, A schematic adapted from Supplementary Fig. S15 showing the general filling hysteresis curve behaviour of the model at different disorder strength and temperatures, between empty to half-full lattice. The model shows transition between a number of stable states, denoted by (stage) numbers, which become more rounded at higher disorder/temperatures. The "M" after the number denotes that the configuration is disordered and mixed. The star denotes the relevant disorder-temperature position of the model discussed in the main text. **b**, Schematic showing the field (potential) driven behaviour of the standard random field Ising model (RFIM) with varying disorder (h) and temperature (T).

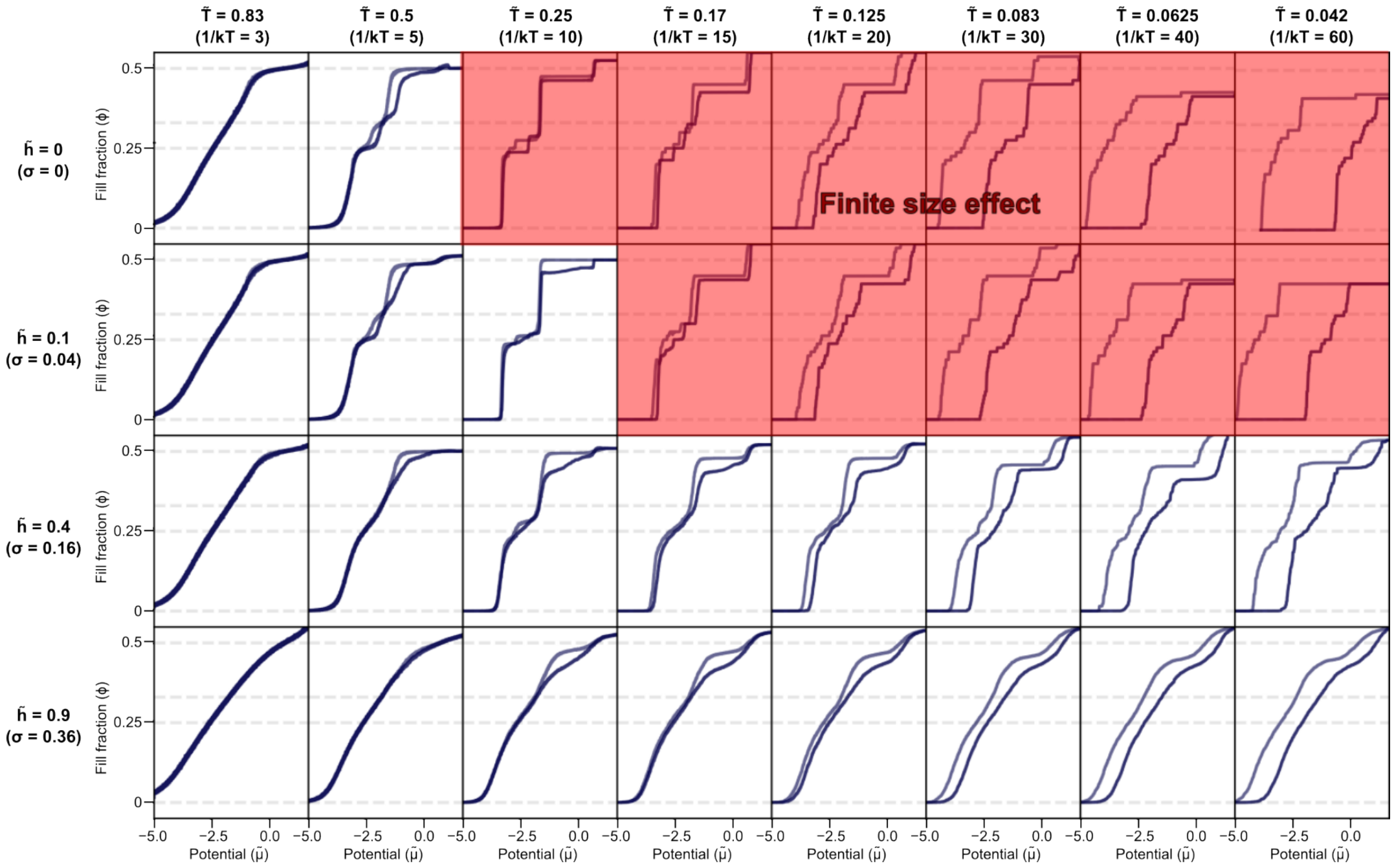

Figure S15 | Model behaviour over temperature and disorder variation. The filling/emptying hysteresis curve vs applied field (potential) drawn for the model over different temperature and disorder. At low disorder strengths and low temperatures, the correlation length in the x direction increases, leading to finite size effect indicated by the red squares. In this regime, the correlation length in the x-direction > lattice size in the x-direction, and the entire row fills at once, leading to step-like behaviour.

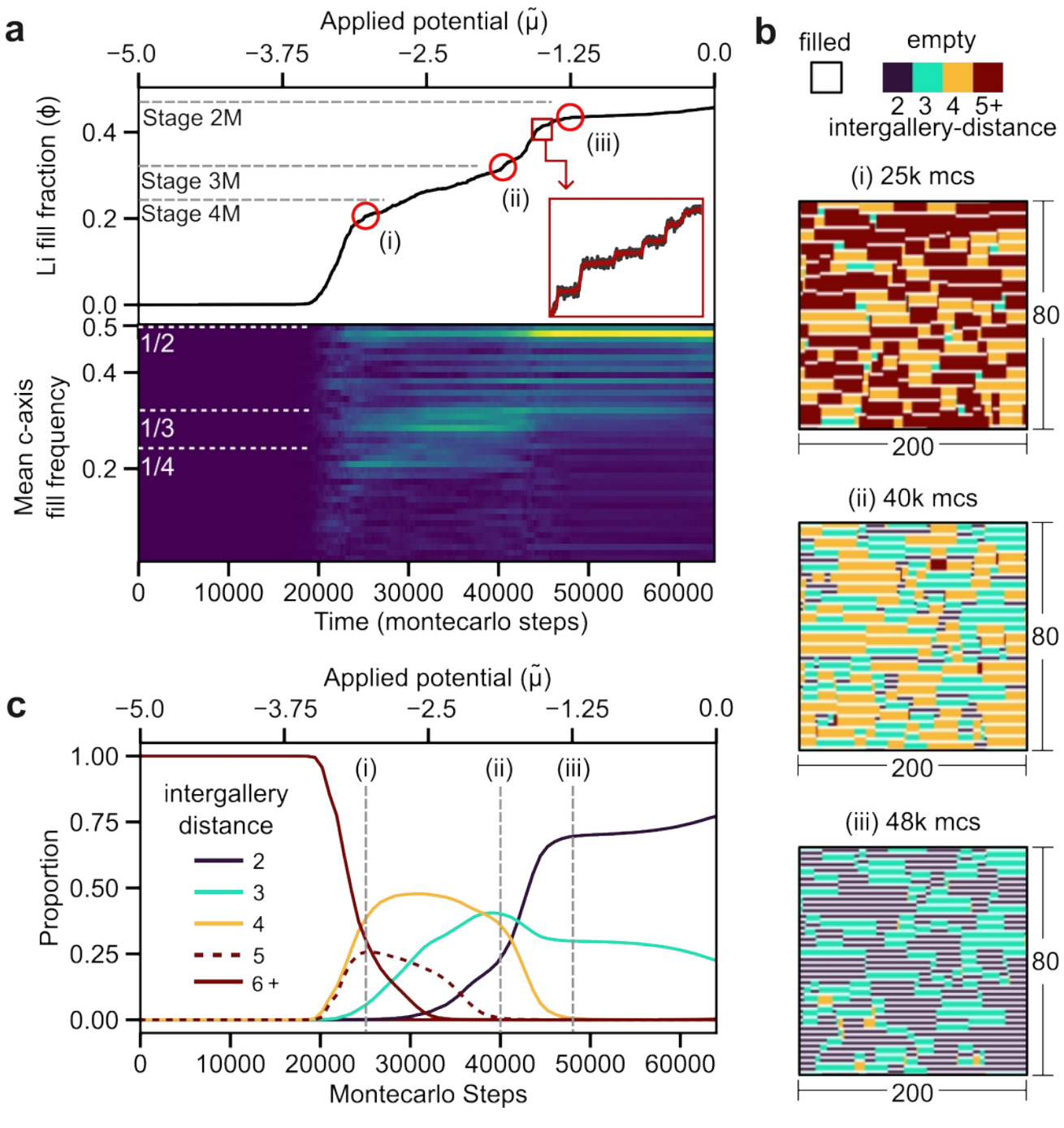

Figure S16 | Behaviour of disordered 2D model. a, Mean lattice fill fraction (ϕ) vs Monte Carlo steps (mcs) during a single ramped potential simulation (upper panel), with disorder ($\tilde{h}$ = 0.4), and temperature ($\tilde{T}$ = 0.125), with total mcs = 80k. Stage 4M, 3M, 2M indicate the disordered mixture of staging distances which, on average, appear to have 1/2, 1/3 and 1/2 filling. Red circles denote time points (i)-(iii) at which the lattice pictures in **(c)** are taken from. Red square inset highlights a portion of the filling curve which demonstrates stepped (avalanche) behaviour (mcs = 45.7k – 47.2k). The respective Fourier transform of the lattice in the c-direction vs Monte Carlo steps (lower panel). **b,** The lattice structure shown at different time points (i)-(iii). Occupied sites are drawn in white, and unoccupied sites are coloured according to the respective c-axis separation between occupied sites. **c,** Every c-direction gap between occupied sites are classified according to the distance, and the statistic is plotted as a population proportion vs time. 80 slices are taken from 0 to 80k mcs. The time points corresponding to **(b)** are marked with a dotted line (i),(ii),(iii). Results only shown up to 64k mcs ($\tilde{\mu}$ = 0) for clarity.

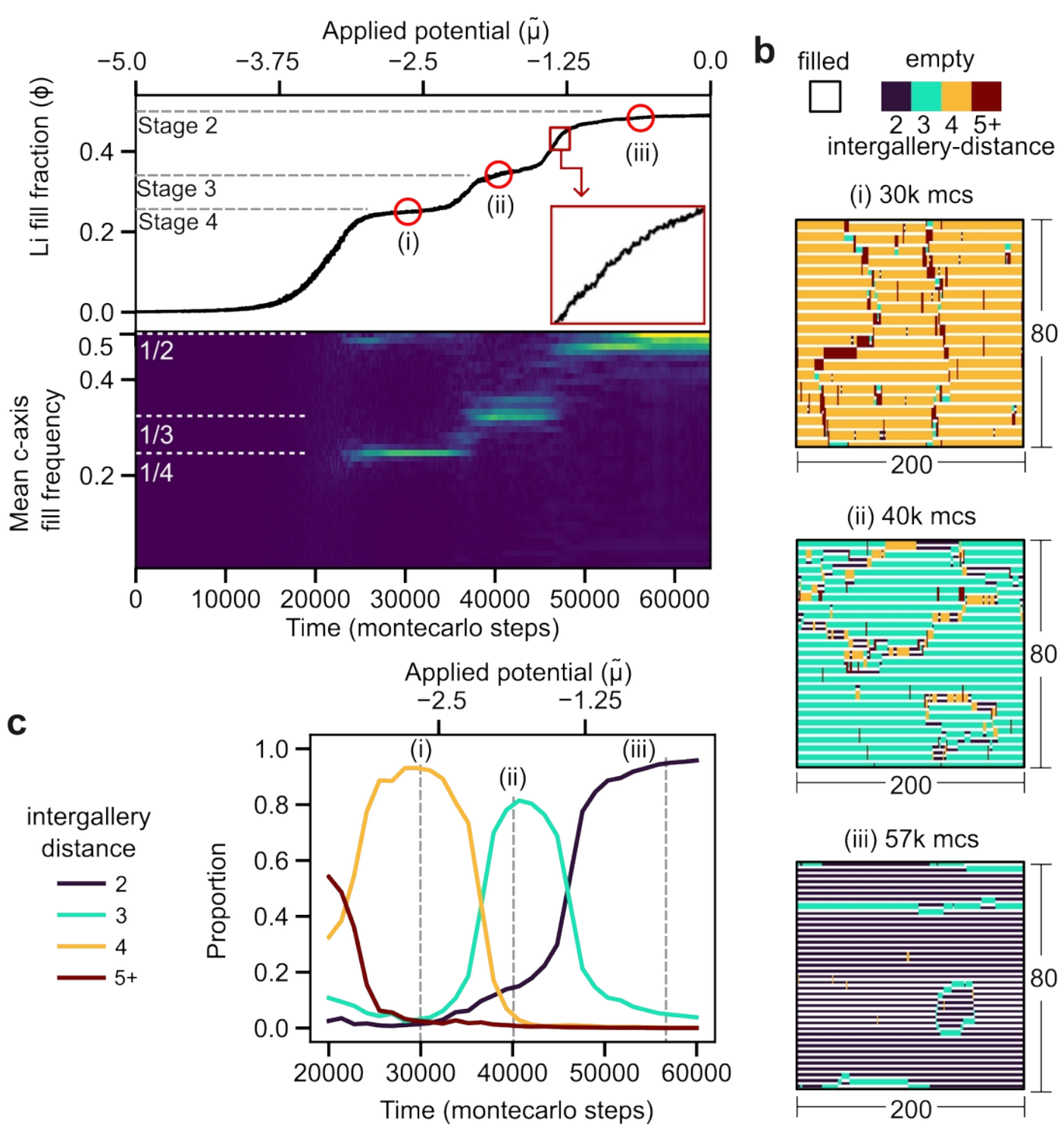

Figure S17 | Staging behaviour of disorder-free, thermally activated 2D model. a, Mean lattice fill fraction vs Monte Carlo steps (mcs) during a single ramped field simulation (upper panels), with parameters $\tilde{h} = 0$, $\tilde{T} = 0.5$, total mcs = 80k. Red circles denote time points at which the lattice pictures in **(b),(c)** are taken from. Close up of the filling curve (inset) demonstrating a lack of avalanches (47.5k – 49.5k mcs). The respective Fourier transform of the lattice in the c-direction vs Monte Carlo steps (lower panel). **b,** The lattice structure shown at different times. Occupied sites are drawn white, and unoccupied sites are coloured according respective c-axis separation between occupied sites. **c,** Every c-direction gap between occupied sites are classified according to the distance, and the statistic is plotted as a population proportion vs time. 100 slices are taken from 0 to 80k mcs, with the previous time slices marked with a dotted line (i), (ii),(iii). Results only shown up to 64k mcs ($\tilde{\mu} = 0$) for clarity.

2.2. Modified 3D random field Ising model

In order to demonstrate that we retain the same qualitative behaviour in the 3D model, we have simulated a 3D lattice with the attractive intralayer coupling extended into the y-direction. The coupling terms and formulation are otherwise identical to the 2D model, and typical parameters can be found in Table S4. Exploration of temperature and disorder parameter space revealed that the transition is qualitatively similar as shown in Fig. S18. The transition is sharp with a large hysteresis at low temperature and disorder, and becomes highly rounded, with hysteresis disappearing at high temperature and disorder. In between the two regimes, at intermediate temperatures ($\tilde{T} < 0.2$) and disorder ($\tilde{h} \sim 1$), we observe a sharp-gradual-sharp trend during filling and emptying. Due to the larger size of the lattice, more simulation steps were necessary to achieve close to quasi-static driving rates. The effect of model driving rate is shown in Figure S19.

An example is shown in Fig. S20a, where the lattice filling dynamic is shown during a filling simulation. We take slices from the lattice at 3 time points (marked i – iii), and show an XY slice (Fig. S20b) and an XZ slice (Fig. S20c). The XZ slice is qualitatively similar to the 2D model, where due to disorder and frustrated interactions, there is a mixture of distances between filled galleries during the simulation and at no point does any pure staging periodicity dominate. A Fourier transform of the lattice, computed by taking the 3D Fourier transform and averaging kx and ky directions, is shown in Figure S20a lower panel. A broad peak, equivalent to the (001) peak in diffraction, appears at approximately k = 0.25, and continuously shifts to k ~ 0.33 in the continuous, sloping region between the “stage 4-like” (4M) and “stage 3-like” (3M) as marked in the upper panel.

In the XY slice (Fig. S20b), we see an emergence of a cluster with a complex spatial distribution. Although in the example shown, there is a single connected cluster, in other layers and simulations, there may be multiple disconnected clusters, which eventually become connected via a series of avalanches. The evolution in a single XY slice, resembles a 2D standard RFIM model, where filling progresses via intermittent clusters due to disorder and athermal dynamics, with the added complication of repulsive interlayer interactions. This can be seen in the filling curve of individual XY layers, shown in Fig. S20d. Interestingly – although there is no dynamic “layer shuffling” or thermal rearrangements due to quenched disorder and athermal dynamics – still some layers may empty while others fill due to the repulsive interlayer interactions. A combined animation of the simulation can be found in Supplementary Video S5.

Table S4 | 3D Model parameters.

J	*K1*	*K2*	*K3*	μ range	*Total mcs steps*	*Lattice size*
1	3	0.75	0.333	-10, -2	100,000	64 (x), 64 (y), 32 (z)

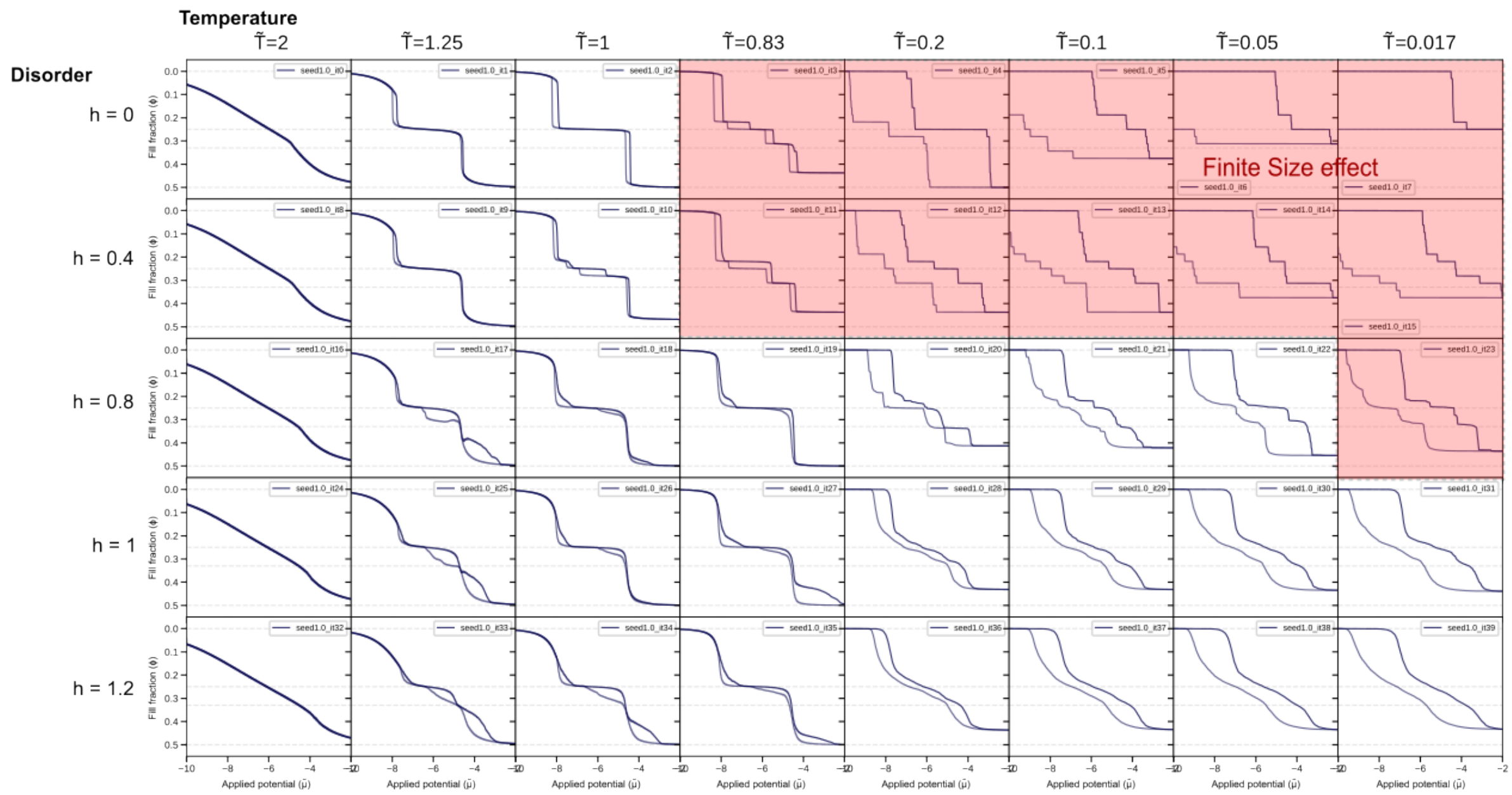

Figure S18 | 3D model behaviour over disorder-temperature space. Parameters can be found in Table S4. Similar to the 2D model, at low disorder strengths and low temperatures, the correlation length in the xy directions increase, and when this correlation length exceeds the xy lattice size, the entire layer fills at once. This finite size effect is indicated by the red squares.

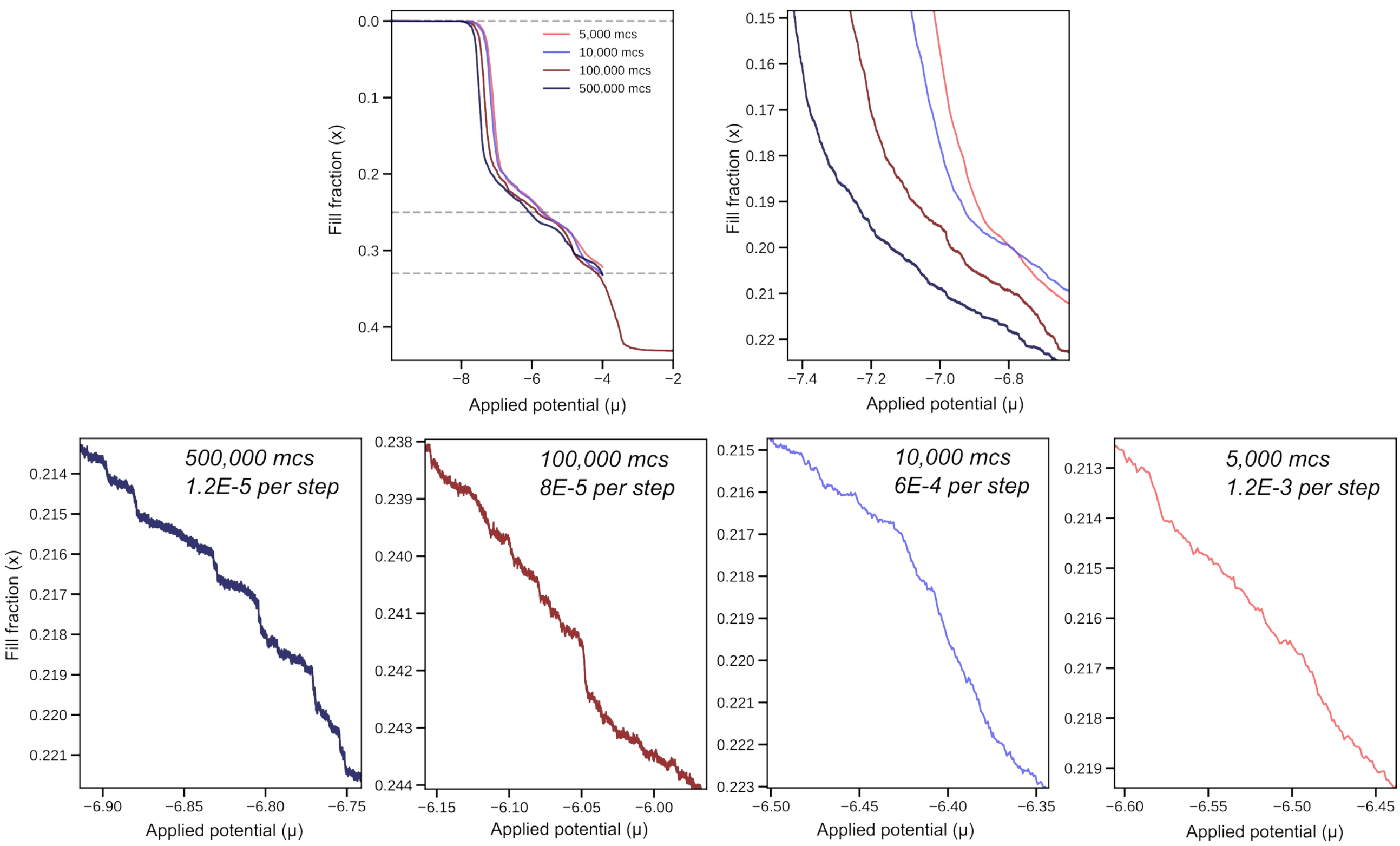

Figure S19 | Driving rate dependence of model. Temperature ($\tilde{T}$) = 0.1, disorder ($\tilde{h}$) = 1. Other parameters as shown in Table S4.

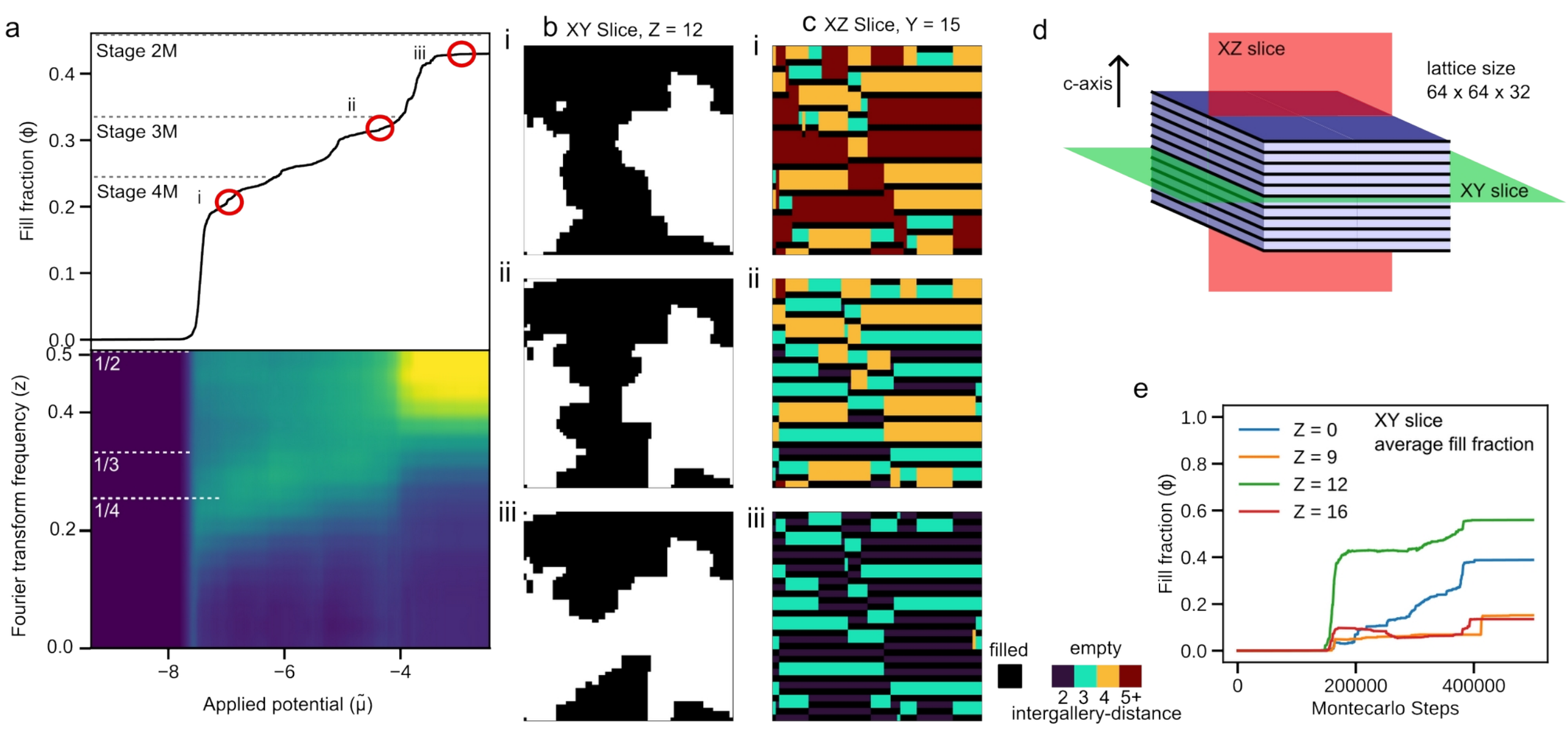

Figure S20 | Example of lattice evolution during 3D simulation. a, The fill fraction during a filling simulation (upper panel) with time points of interest marked with a red circle. Stage 4M, 3M, 2M indicate the disordered mixture of staging distances which, on average, appear to have 1/2, 1/3 and 1/2 filling. A 3D Fourier transform of the lattice is averaged in the kx and ky direction, and its evolution over the simulation is shown as a heat map (lower panel). **b,** An XY slice at Z = 12, and **c,** an XZ slice at Y = 15, are shown at various times as marked in (a). **d,** A schematic demonstrating an XY and XZ slice in the lattice. **e,** A selection of XY slices is extracted, and their individual mean fill fraction is plotted vs simulation time. Intermediate temperature ($\tilde{T}$ = 0.1) and disorder ($\tilde{h}$ = 0.9), with 500k total Monte Carlo steps in 64 x 64 x 32 lattice size was used for the simulation.

2.3. Further discussion of model results

In Fig. S16, we simulate a lattice with medium disorder ($\tilde{h} = 0.4$) at low temperature ($\tilde{T} = 0.125$) and plot its mean fill density (ϕ) with time, this is same as shown in the main text. The filling curve (Fig. S16a upper panel) shows a sharp transition from empty to stage 4-like filling (ϕ~0.21) (i), then a sloping transition from quasi-stage 4 to quasi-stage 3 (ϕ~0.31) (ii), and finally a sharp transition from quasi-stage 3 to quasi-stage 2 (ϕ~0.45) (iii). Lack of pure staging is demonstrated by the broad, ill-defined peaks in the c-axis Fourier transform of the Li-ion positions (Fig. S16a lower panel). To further highlight this aspect, it is instructive to evaluate the c-axis distance between occupied sites in the lattice. For a system in an equilibrium stage-n phase, we expect these distances to be equal to n, except for domain interfaces and random thermal fluctuations. However, at every time point there exists always a mixture of distances (Fig. S16c) and critically, despite this, the model follows the same experimental filling trend. We label the critical points (i)-(iii), stages 4M, 3M and 2M respectively, with M implying a highly mixed stage-like character to distinguish them from true stages with long range order.

At zero disorder ($\tilde{h} = 0$) and higher temperature ($\tilde{T} = 0.5$), the model can exhibit true stages without mixing disorder. This is illustrated in Supplementary Fig. S17, where fast thermal rearrangements and long range correlation allows system wide transitions between equilibrium phases of empty, stage 4, 3 and 2. This demonstrates that static disorder and close to athermal dynamics can break up interlayer correlation, leading to local domains, hysteresis, avalanches and metastability. Due to decaying interlayer repulsion, the system is most affected by disorder for higher order stages, resulting in a rounded, continuous-like transition between "stage 4-like" and "stage 3-like" filling periodicities.

The significance of static disorder on phase transition behaviour is well studied in standard RFIM, where long range correlation, allowing a sharp, system-wide first-order transition, is broken by disorder and thermal fluctuations, thereby making the transition more rounded and continuous. Near the critical line, disorder allows the formation of finite metastable domains, and avalanches appear as each local domain fills (Fig. S14).

2.4. Further model-experiment comparison

We have already discussed the comparison of experimental trends to what is expected from a general avalanching system, in particular the power law (Section 1.2.1) size distribution, driving rate dependence (Section 1.2.2) and sample disorder dependence (Section 1.2.3). In addition, in the main text, we have discussed the effect of single particle local disorder on avalanche spread (temporally) and avalanche size distribution. Here we outline some of similarities and features from the 2D and 3D model that can be compared to the experiment.

The discussion of experimental temperature trend is more subtle but still notable. See Fig. S21a,b which shows the the 2D model filling/emptying trend and avalanche size distribution as a function of temperature. It is consistent with previous studies in standard RFIM(Yao & Jack, 2023) where at higher temperatures, (1) large (high $\Delta\phi$) avalanches occur less frequently and (2) smaller avalanches occur more frequently, due to thermal fluctuations exciting smaller avalanches and consequently preventing the build-up required for large avalanches to occur. However, note the experimental trend (Fig. S22) does not change substantially, which is due to the experimental temperature range (T = 288 K to 318 K, approx. 10% increase) being far smaller than that presented in the model ($\tilde{T} = 0.042$ to 0.125, approx 300% increase in Fig. S21b). Over a similarly narrower temperature range (Fig. S21c), a trend in the avalanche size distribution is too small to be visible in the simulation statistics. Similarly, there is only small changes in the hysteresis over this narrow range, which indicates that the experimental trend in the avalanche statistics and voltage hysteresis is more related to reaction overpotential and mass transport.

Interestingly, higher temperatures lead to thermal-fluctuation induced back-hopping, leading to step changes in the opposite direction of the driving force. These reverse hops are evident even within a narrower temperature window, and shown in Fig. S23a, where the total number of avalanches increases with temperature along with an increase in the number of reverse hops. We also experimentally detect these reverse hops, where a local region seemingly delithiates during a lithiation cycle and vice versa as demonstrated in Fig. S24. These reverse hops become more frequent at higher temperatures (Fig. S23b), supporting the idea that the avalanches are thermally activated jumps between shallow metastable states. Critically, it implies that we are in a regime where thermal fluctuation, disorder strength and long-range coupling energies are all relevant.

The non-equilibrium transition pathway demonstrated by the simulations can give rise to asymmetry in the filling vs emptying behaviour. This is seen in Fig. S21a, lower panel, where the differential fill fraction (equivalent to the experimental dQ/dV curve) is calculated over 140 simulation runs, and shows 4 peaks during filling, and 3 during the emptying simulations. This originates from the fact that we have a mixture of many intergallery distances which become un/stable at certain potential ranges, but due to disorder, this potential range is broad and overlaps significantly unless slowly ramped. This is illustrated in Figure S26. The experiment dQ/dV also possess asymmetry, however it is reversed, with 3 clear peaks during lithiation and 4 during delithiation. Nonetheless, the results may differ with a slower simulation in a 3D lattice, and we speculate that similar effect may cause asymmetry in the experiment.

An interesting consequence of static (quenched) disorder is that the model runs are sensitive to the structure of this randomness. As shown in Fig. S25a, when the disorder structure is kept the same, the model returns similar results over many runs, as seen by the similarity of the set of green or red traces, respectively. The model supports our experimental observation presented in Fig. S25b, whereby each spatial region of the graphite particle displays similar curves with avalanches at similar positions over different cycles, providing evidence for a unique, structural disorder in each region.

Finally, as discussed in the main text, each avalanche in the model is usually a result of a small domain in a single layer filling. This is demonstrated in the 3D model case, in Figure S24, where a few selected avalanche events are shown as a lattice differential in a 3D and 2D map. Each step corresponds to a connected cluster in a single layer filling up, where the cluster size and morphology varies widely. A comparison of Supplementary video S1 and S5 is interesting here.

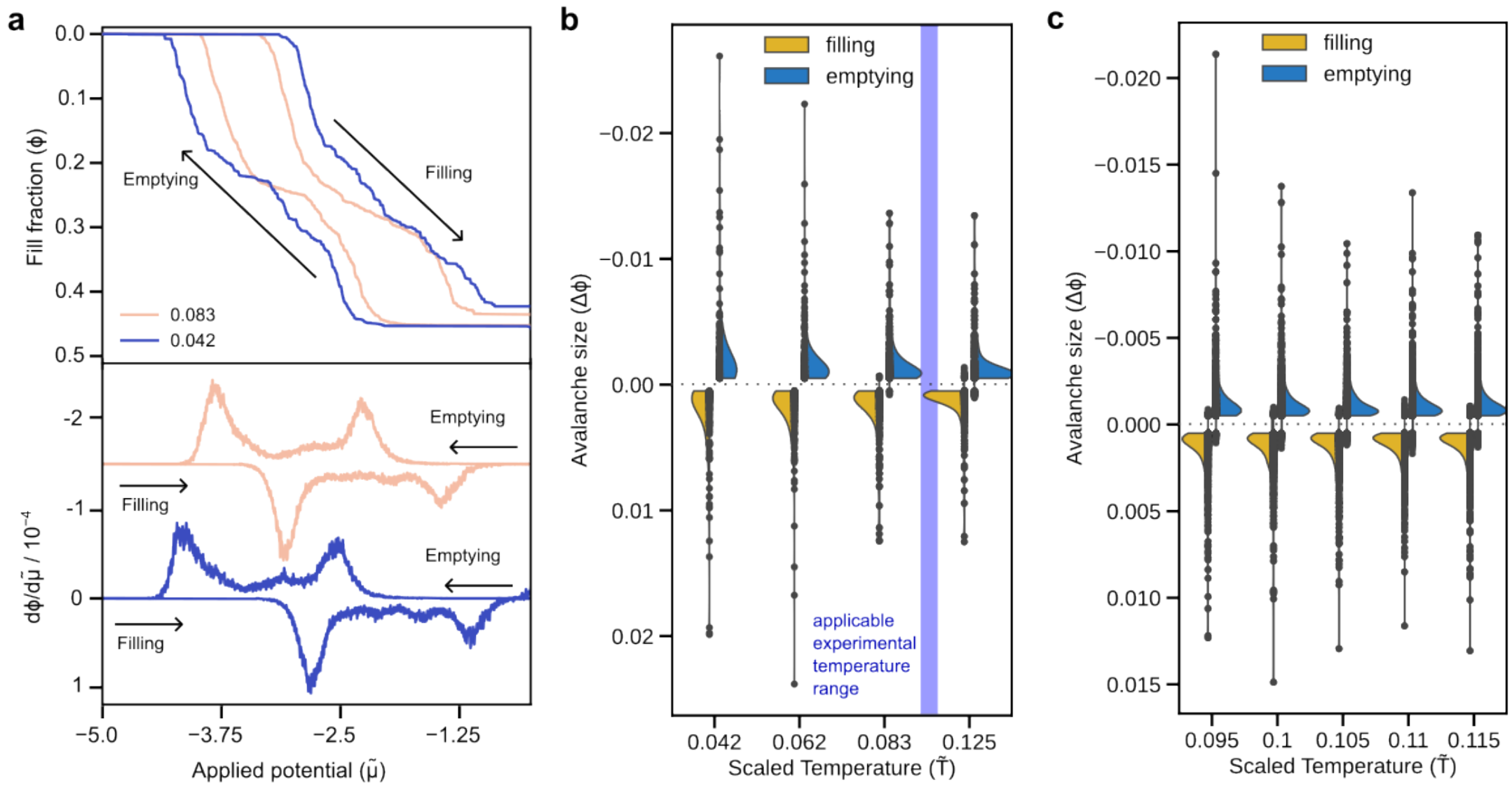

Figure S21| Detailed hysteresis curve analysis and avalanche statistics from 2D model. a, Mean lattice fill fraction (ϕ) vs applied potential ($\tilde{\mu}$) during filling and emptying simulations at different temperatures for the disordered model ($\tilde{h}$ = 0.4, mcs = 80k) for a single run (upper panel). The differential fill fraction per potential ($d\phi/d\tilde{\mu}$) during the filling/emptying simulations for the corresponding temperatures (lower panel). The differential signal was calculated from an average of 140 runs using a low pass filter (Butterworth, 1/50). **b,c,** Violin plots showing the detected avalanche size distribution at different temperatures with dotted line drawn at zero intensity. Over a wide (~300% increase) temperature range (a), and a narrower (~20% increase) temperature range, corresponding better to the experimental temperature range. Statistics from a single filling/emptying run per temperature in the disordered model ($\tilde{h}$ = 0.4, mcs = 80k), where size is defined as the magnitude of the change in average fill fraction. Y-axes has been reversed in order to aid comparison of simulation to experiment.

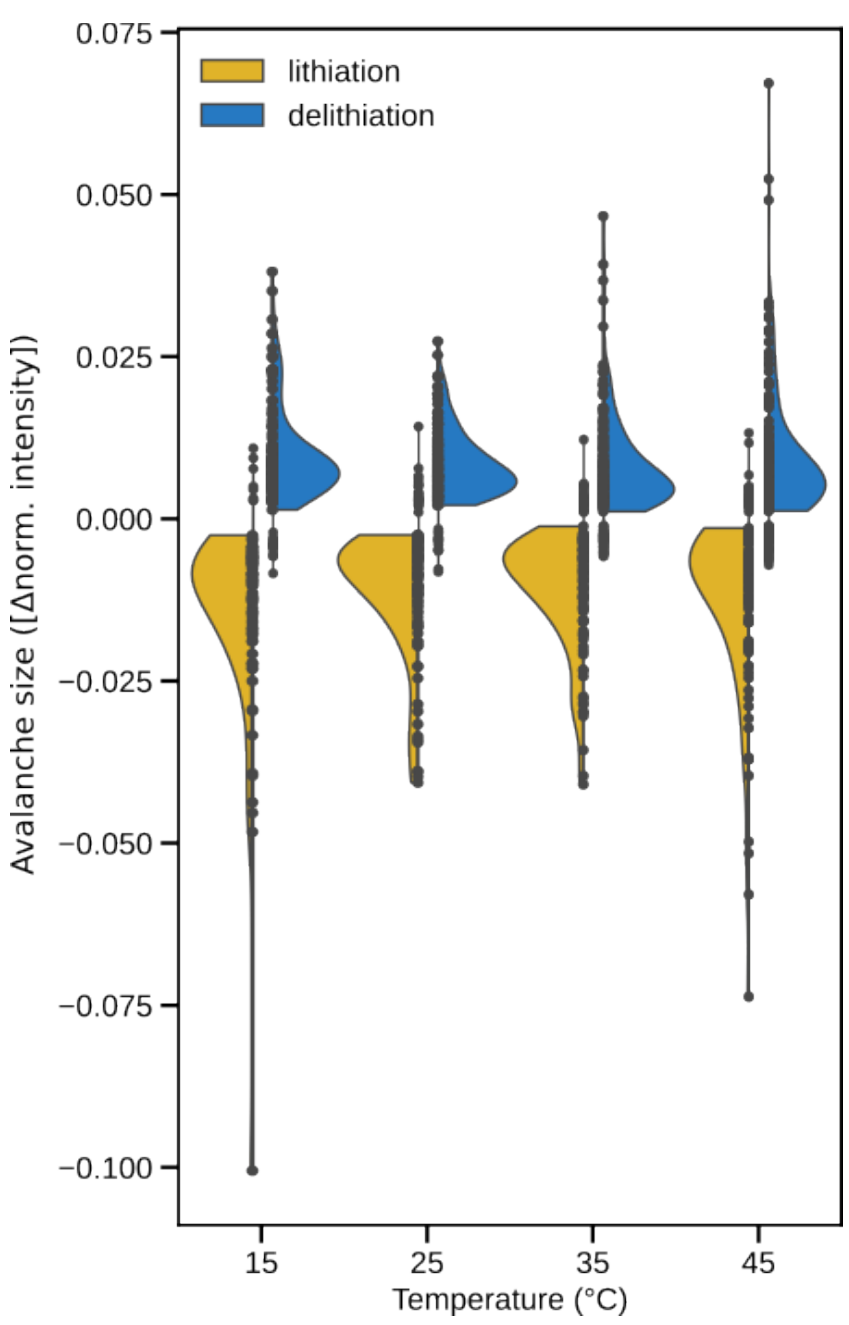

Figure S22 | Avalanche statistics from experiment. Violin plots showing the detected avalanche size distribution at different temperatures. Statistics from experiment during cycles 1 and 2, with de/lithiation at all temperatures, where size is defined as change in the mean optical intensity. The experiment statistics were collected only from the 1L-4L window, from regions A, B and C which have similar levels of disorder.

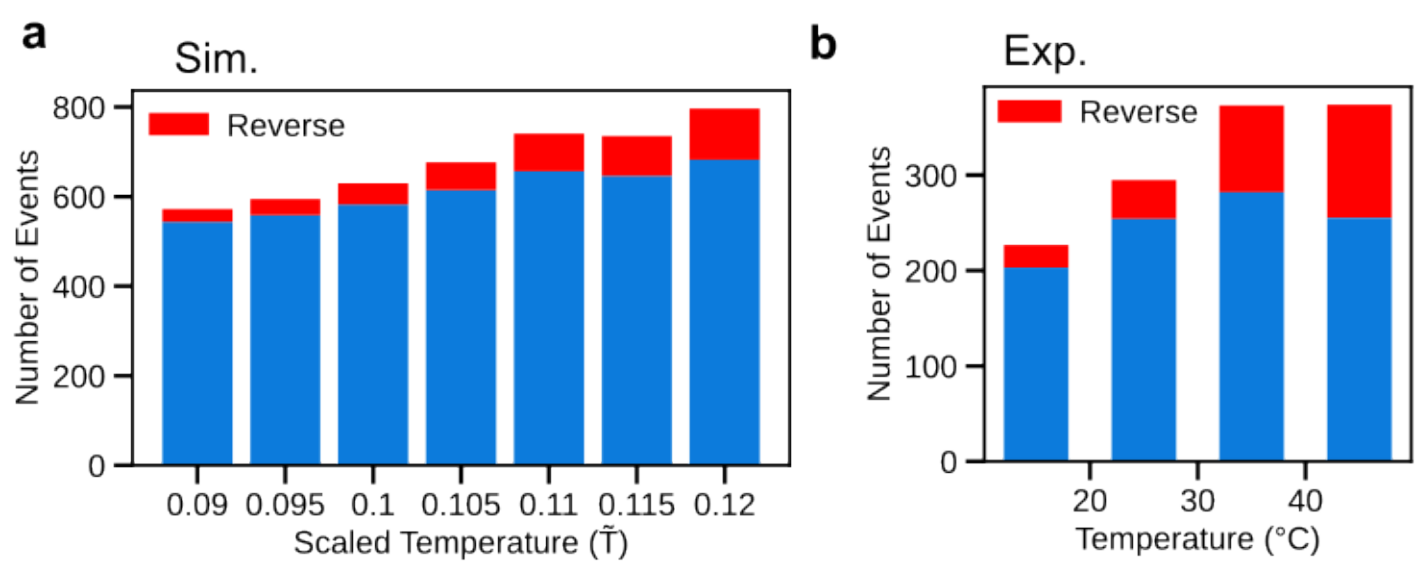

Figure S23 | The number of detected avalanches. The number of detected avalanches as a function of temperature, with the fraction corresponding to reverse events (thermal-fluctuation back-hopping) marked in red. From the 2D model (**a**) and the experiment (**b**). Total number per cycle, per run. The model corresponds to the same dataset shown in Fig. S19. The experiment statistics were collected only from the 1L-4L window, from regions A, B and C which have similar levels of disorder.

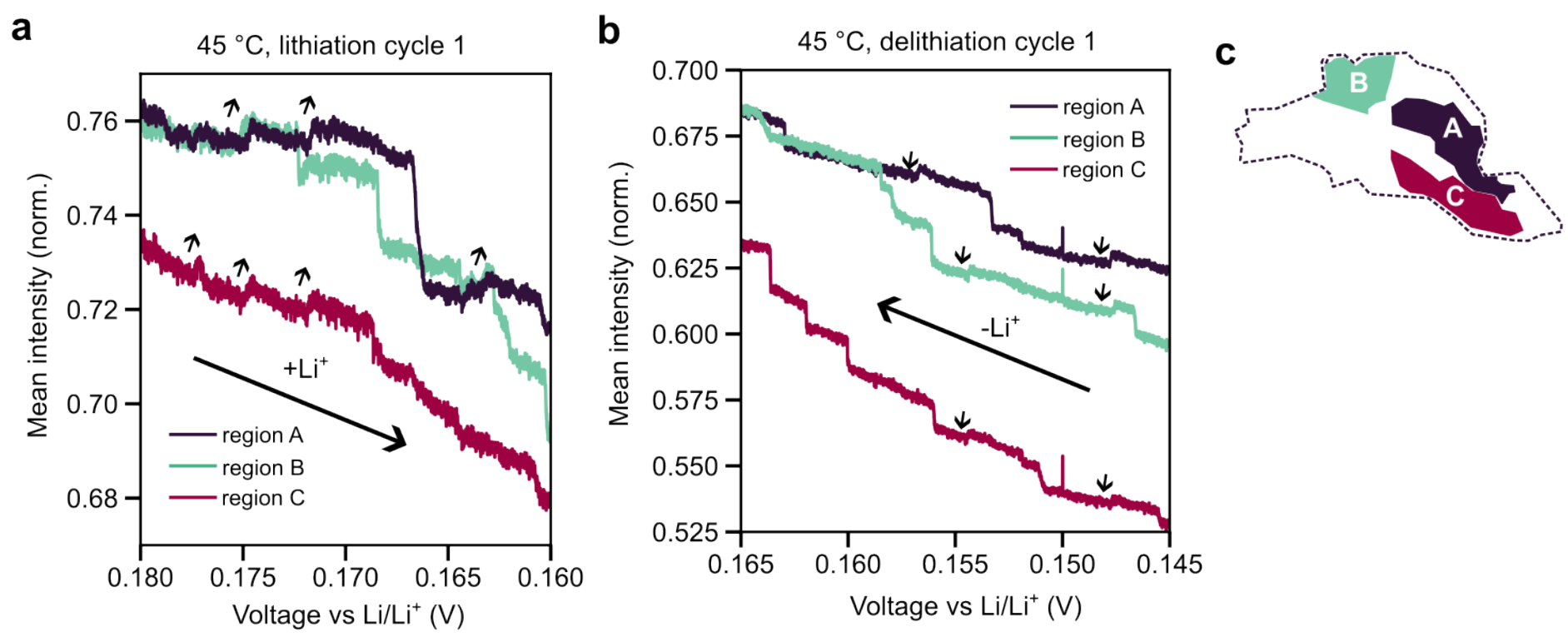

Figure S24 | Illustration of experimental reverse avalanches. Mean intensity (normalised) vs Voltage shown for three selected regions during 45 °C (a) lithiation and (b) delithiation. Both de/lithiation cycles and all regions show "reverse avalanche" behaviour, where there is a step up (during lithiation) or step down (during delithiation), going against the overall trend. These small steps are marked with an arrow.

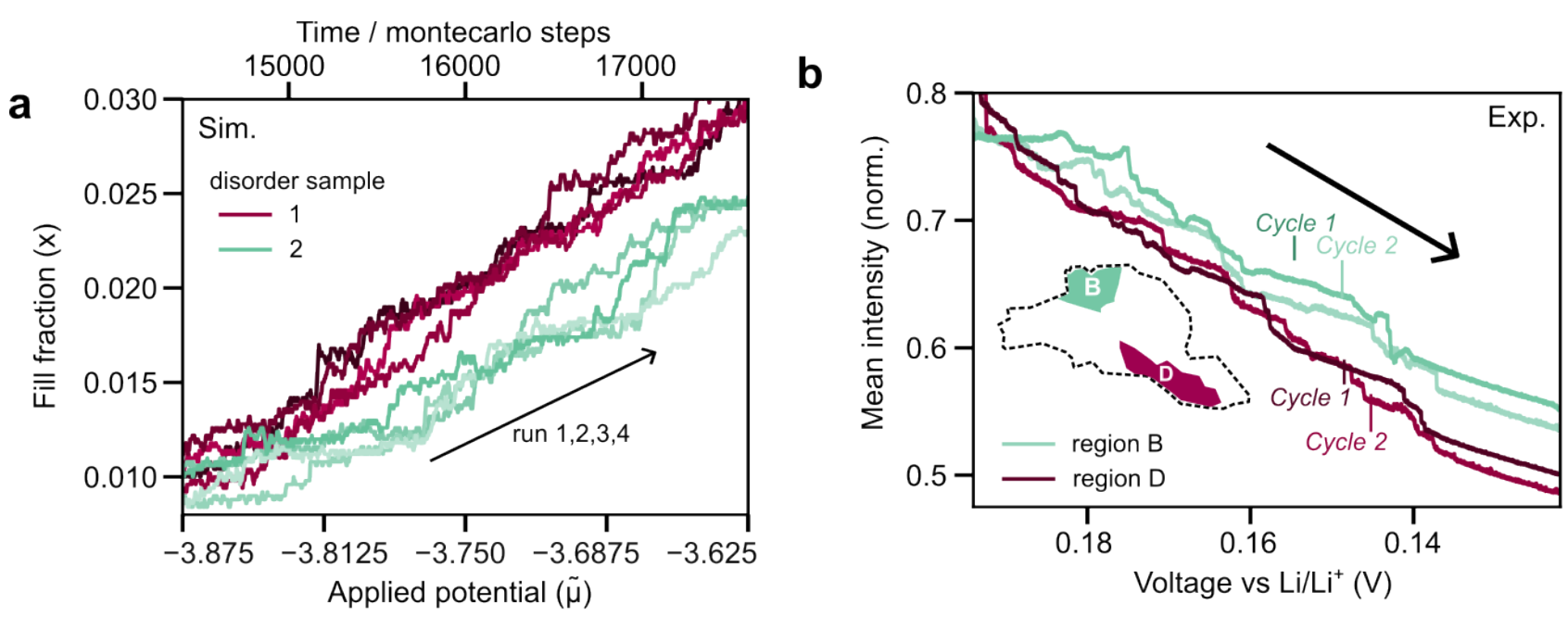

Figure S25 | Memory effect on cycle to cycle reproducibility a, Demonstration of quenched disorder effect for the high-disorder case. ($\tilde{h}$ = 0.9, $\tilde{T}$ = 0.0625, mcs = 80k). The simulation is repeated 4 times each with disorder sample 1 (red) or sample 2 (green). **b,** Experimental optical intensity trace during lithiation (15 °C, C/20) in the first and second cycle with the x axis interpolated to the cell potential. Mean intensity from 2 different regions (marked B and D) shown.

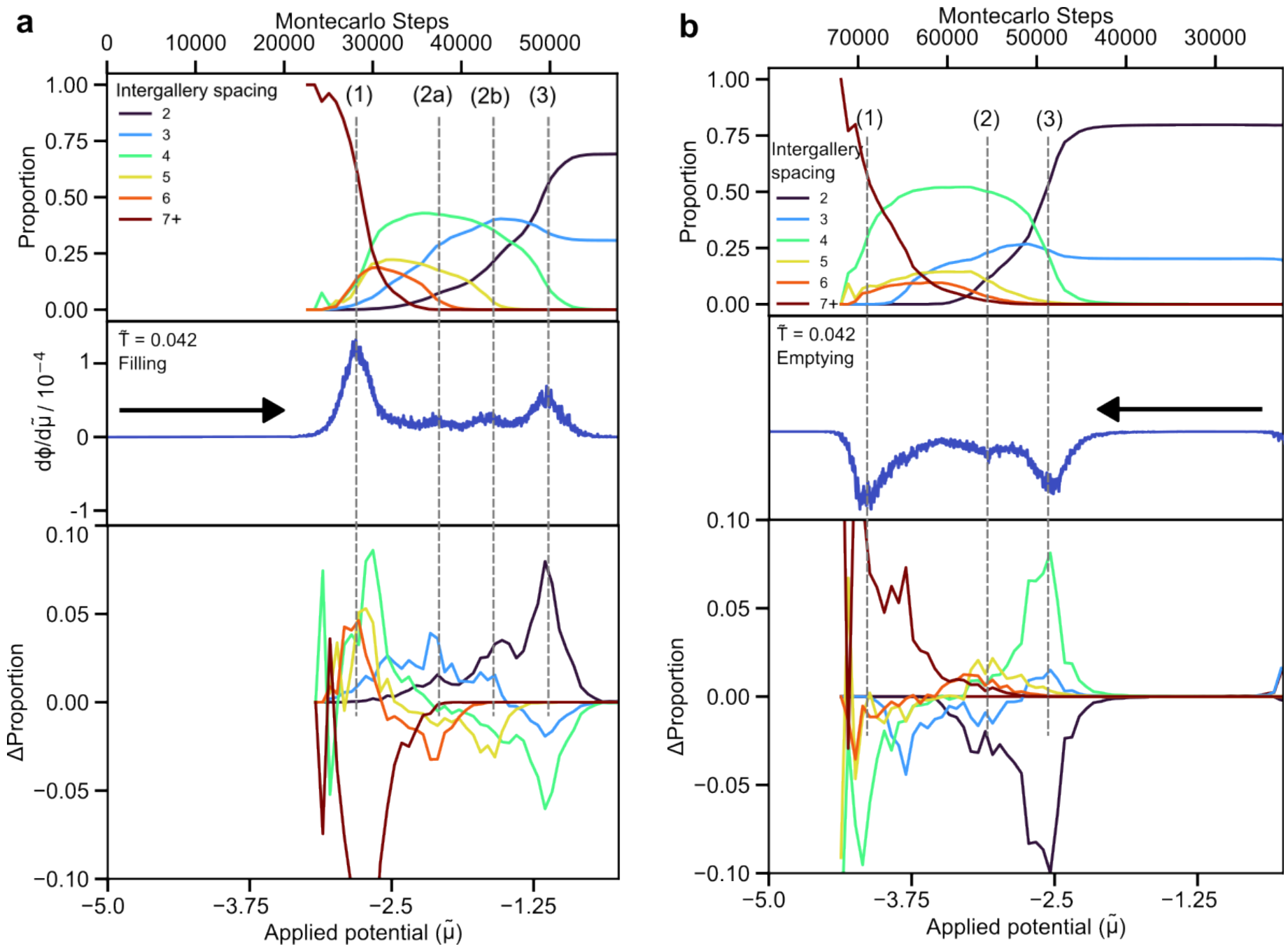

Figure S26 | Population dynamics of intergallery spacing in the model. a,b, Staging population dynamics during filling (**a**) or emptying (**b**) at $\tilde{T} = 0.043$, $\tilde{h} = 0.4$. Statistics gathered over 140 runs. The top panel shows the population of intergallery spacing vs time. The intergallery spacing is defined as the vertical distance between filled lattice sites in the model. Every empty lattice blocks is classified as either 2, 3, 4, 5, 6, or 7+ according to the distance between the respective filled sites. The middle panel shows the overall differential filling curve, with dotted vertical line drawn at peak positions. The bottom panel shows the time differential of different spacing populations. Small peaks in the differential filling curve can be interpreted as a result of population dynamics. For example, peak 3 in (**b**) could be interpreted as the conversion from Stage 3 and 4, to Stage 2 spacing.

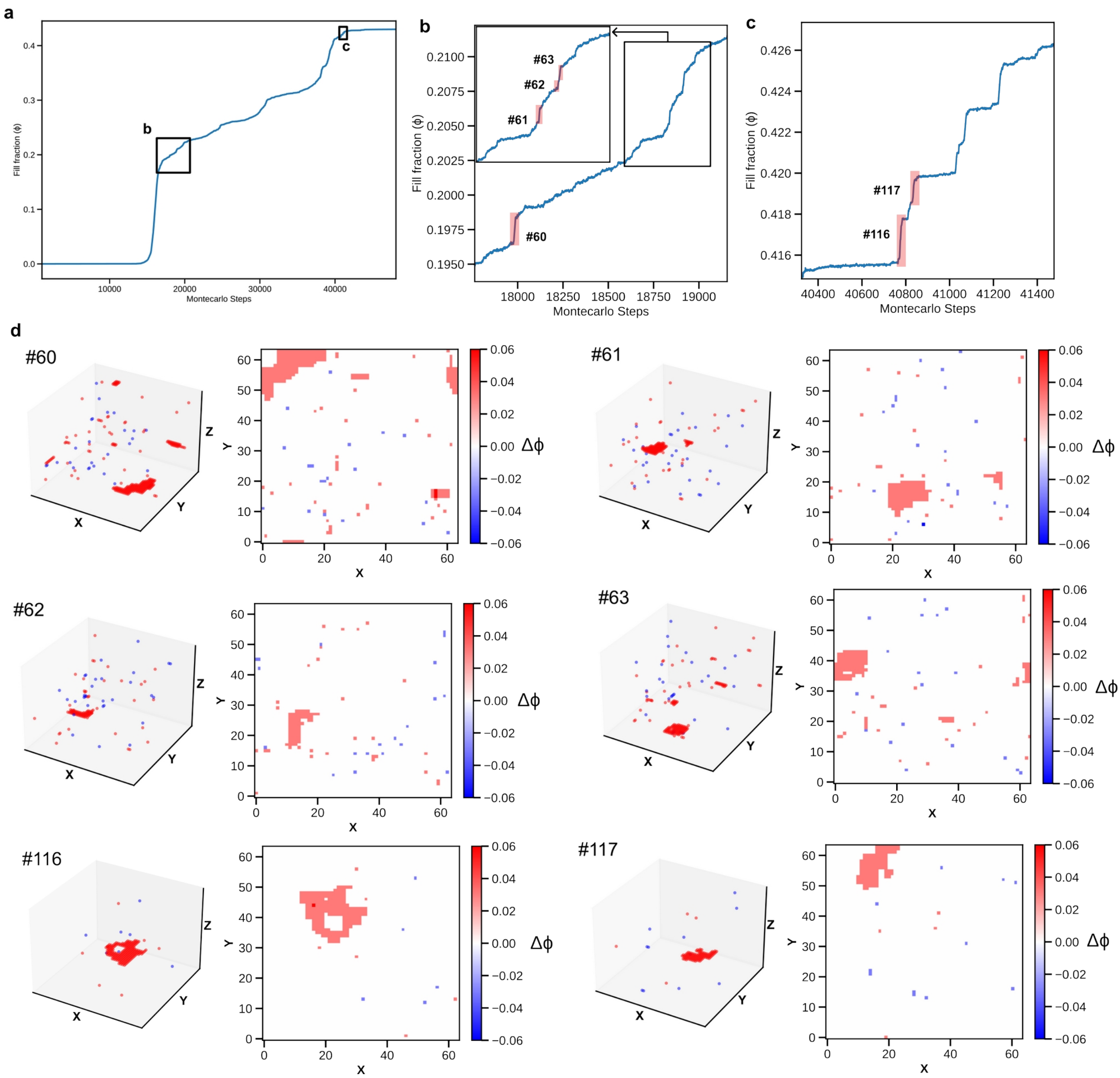

Figure S27| Origin of avalanches in 3D model. a, Mean fill fraction curve during filling simulation from 3D model same as shown in S18. Small squares indicate areas selected for the zoom-ins. **b,c,** Zoomed in area showing clear avalanche steps. Red squares highlight single avalanches, which are numbered. **d,** Lattice picture of highlighted and numbered avalanches from (b) and (c). The change in lattice occupation during a single detected avalanche is calculated and shown in a 3D schematic (left) or a 2D XY averaged map (right). Red indicates filling and blue indicates unfilling.

2.5. Model limitations and alternative mechanisms

We have shown the results of RFIM as a minimal model that reproduces (1) staging and (2) avalanches under the simplest conditions. To do so, it incorporates uncorrelated disorder (random field) and coupling constants required to form staging compounds (Schon et al., 1988) in a discrete lattice. It is meant as a useful qualitative model reproducing many emergent experimental features and linking them to a well known theoretical framework, but not as a quantitative model. We apply periodic boundary conditions and assume that our experimental results are representative of bulk behaviour. We also assume that the dominant dynamics is that of quasi-static evolution via metastable minima in the lithium configurational energy landscape under slow driving. Thereby, we neglect diffusion and surface reaction by assuming that, on the

simulated time scales, the reaction overpotential is small at slow C-rates (Supplementary Section 1.4) and that the solid state ionic diffusion is sufficiently fast ($x^2/2D$ ~ 0.1 s (Persson et al., 2010)).

This motivates future work on a more realistic, quantitative model with continuum, phase field formulations, similar to those previously applied to battery material systems (Chandesris et al., 2019; Fraggedakis et al., 2020; Smith et al., 2017) incorporating diffusion and surface reaction boundary conditions. Quenched disorder may also be realised as a random variation in coupling constants rather than site occupation energy, much like the discrete random bond Ising model(Vives & Planes, 1994a). In fact, although the disorder is spatially uncorrelated in the current model, the particle disorder has been partially experimentally characterised as arising from elastic deformation and kinks (see Section 1.1). Such correlated disorder can explain the emergence of persistent cluster regions, as seen in the experiment, and the spatio-temporal correlation between them, which our model is unable to reproduce. In order to reproduce intra-particle correlations discussed in the main text, it may be necessary to investigate coupled systems or to incorporate spatially correlated disorder to subdivide the simulation lattice into smaller regions.

We stress that (1) avalanches are not necessarily uniquely associated with specific regions of the graphite particle, their size and location are random and broadly-distributed (Supplementary Section 1.2.1), and (2) disordered features which can contribute to break-up of correlation and formation of metastable states span a wide range of length scales, whose impact on Li dynamics is difficult to quantify. Furthermore, lattice disorder may arise from the lithium insertion process itself, such as layer opening, shear stresses due to changes in layer stacking, and lattice expansion and stress redistribution. Indeed, a self-generated disorder with cycling has been previously reported in Martensitic transformations (F. J. Pérez-Reche et al., 2009; F.-J. Pérez-Reche et al., 2007) which is another prototypical system demonstrating avalanches and return point memory(Amengual et al., n.d.; Sethna et al., 1993, 2001). Together, these factors highlight both the challenges and the importance of developing more quantitative models incorporating realistic disorder.

Section 3: Supplementary Methods

3.1. Image analysis

All post-acquistion image processing and analyses were done using python, using numpy multidimensional arrays and h5py for data saving and management in hdf format. First, each experiment was post processed to correct for x-y drift. To do this, a small frame around the particle was selected and edge detected (canny edge detection, k1 = 100, k2 = 150). The relative translation was found using phase correlation on the edges (between n^{th} and 0th frames). Using the relative x-y jitter, the frames were corrected using 2D interpolation (python fast_interp package) and saved for further processing.

For facile processing and analysis, the dataset was segmented into different temperatures (15, 25, 35, 45 °C), cycles (1 or 2), and lithiation/delithiation (see Fig. S28). For each segment, the data size was reduced by masking for pixels within the particle and saving them as a sparse array for later reconstruction. Similarly, the initial regions (regions A, B, C, D, as shown in the main text) were selected manually by masking out reoccurring regions observed in the differential frames and saved individually as a sparse array. To calculate the normalised mean intensity, the mean intensity from the initial 10 frames (final 10 frames for delithiation cycles) were averaged from the respective dataset (region, particle or entire frame), and used to normalise the mean intensity trace.

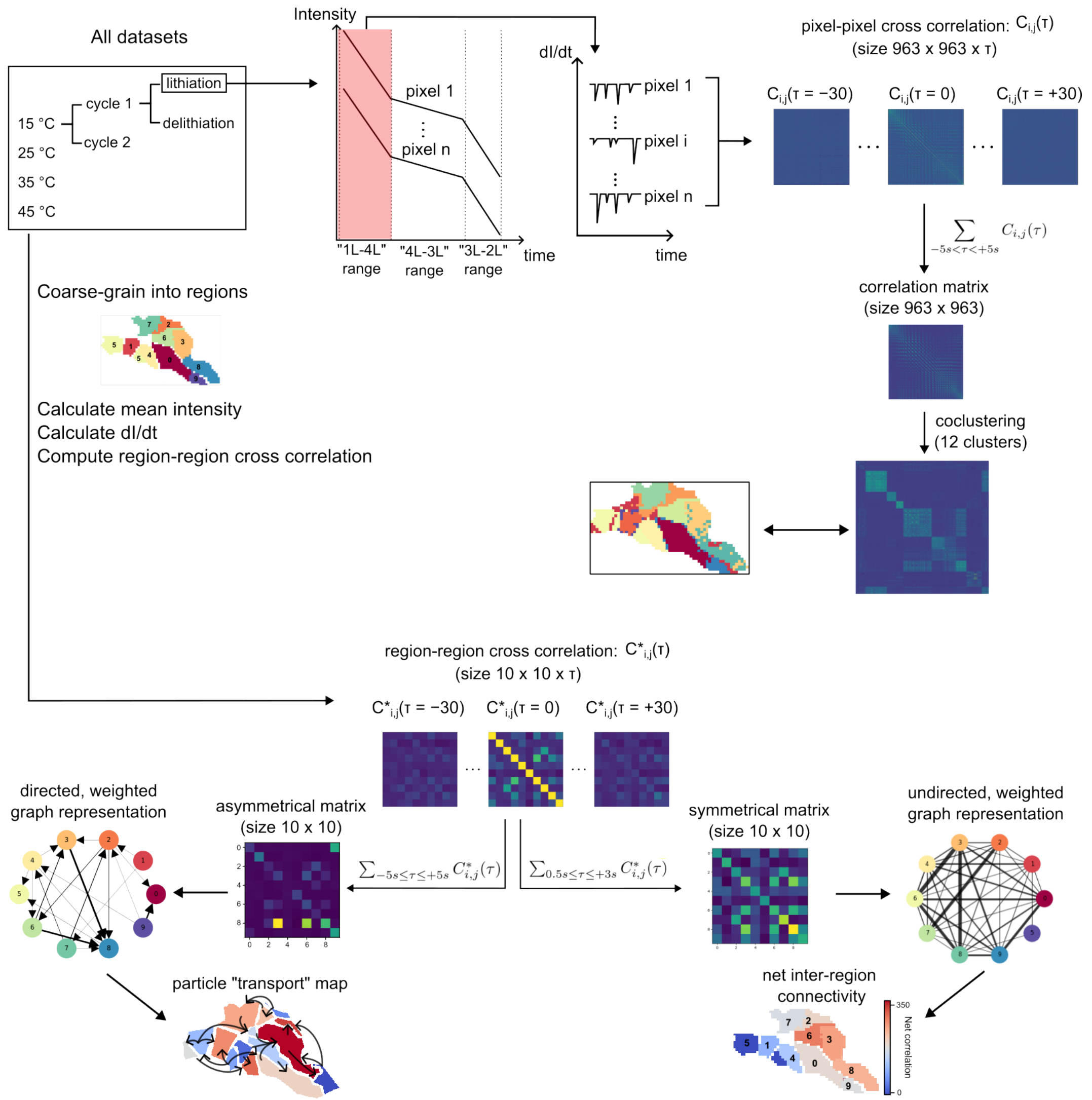

Figure S28 | Schematic showing the data processing pipeline for spatio-temporal correlation discussed in the main text.

3.2. Spatio-temporal correlation analysis

For the image time series taken during the dilute stages, the mean intensity predominantly consists of steps with some noise, hence resulting in its differential time series (dI/dt) being composed of "pulses". A cross correlation between a time-series of pulses at pixel-i and at pixel-j will describe the likelihood of a step-event (avalanche) at pixel-j occurring a certain time-lag τ after a step-event at pixel-i.

The data processing pipeline is shown in the schematic in Figure S28. Our experimental dataset consisted of sets from different temperatures, cycles, and de/lithiation. Each set was further subdivided into three time ranges, corresponding to the sharp – gradual – sharp fall in mean intensity. This was necessary because the behaviour near the start ("1L-4L" range) and end ("3L-2L" range) of the dilute stages showed different temporal characteristics. The processing was performed separately on each set and time-range, where the 2D image series was spatially binned (5 x 5 pixels), and the pixels interior to the particle (963 pixels) was stored as a flattened time-series array (time x pixels). The boolean mask corresponding to the interior pixels was the same over different datasets except for some minor translation and rotation to account for the slight x-y drift between measurements. An edge preserving smoothing filter (median filter, window size = 7.5 s) is applied to the time axis and subsequently the differential time series, dI/dt – defined as frame [i] – frame [i-1] (time step = 0.5 s) – is calculated. Each time series is then mean subtracted and normalised and the cross correlation function was computed from this time series. Specifically, the time-lag (τ) dependent discrete cross correlation was computed between every pair of pixels (i,j), for the processed time series ($C_{i,j}(\tau)$). For analysis, the cross correlation function is summed over a time-lag window to obtain a correlation matrix which contains information on the pixel-i – pixel-j correlation within the summed time window.

To obtain the particle regions, the correlation function was summed from -5 s to +5 s ($\sum_{-5s \leq \tau \leq +5s} C_{i,j}(\tau)$, 15 °C, lithiation cycle 1) and spectral coclustering was performed (Dhillon, 2001) with 12 clusters on the resulting correlation matrix. Two of the clusters, corresponding to edge states, were discarded and the resulting clusters were used with minor adjustments as the 10 regions as outlined in Figure S25. The result of clustering with various parameters is explored in Figure S29.

The original dataset consisting of 963 pixels was coarse grained into 10 regions, using the masked regions found above. The boolean masks were used with minor translation and rotation between sets. The mean intensities from each region were used to recompute the correlation function ($C^*_{i,j}(\tau)$) with the same steps as previously described, namely the mean intensity of each region was median filtered, and its differential time series were mean subtracted and normalised before calculating the region-region cross correlation. The correlation matrix then describes the region-i to region-j correlation at the given lag-time.

The total inter-region correlation (main text Fig. 4b) was found by summing the correlation function over a large time window ($A^*_{i,j} = \sum_{-5s \leq \tau \leq +5s} C^*_{i,j}(\tau)$) and summing the cross correlation values for each region except for self-correlation (net correlation for region i = $\sum_{j, i \neq j} A^*_{i,j}$). This analysis was performed for all datasets (all temperatures, de/lithiation, cycles 1 and 2), and the average value for each region is found and shown as the colormap on the particle in main text Fig. 4b. Note that the inter-region correlation values did not differ significantly between temperature and de/lithiation cycles.

Next, a directional relationship between regions was calculated by summing the correlation function over non-zero lag-times. To produce the main text Fig. 4c, the correlation function is summed between 0.5 s and 3 s ($A^*_{i,j} = \sum_{0.5s \leq \tau \leq +3s} C^*_{i,j}(\tau)$), which results in a asymmetrical correlation matrix. A positive value in

the matrix was interpreted as an event in region j being likely to occur after event in region i, within the time window, here [0.5s, 3s]. This can be mapped onto a graph with the nodes as the particle regions. Instead of constructing a bi-directed graph, we used the difference between symmetric element as the weight between nodes. Specifically, for $A^*_{i,j} - A^*_{j,i}$ there would be a directed node from j to i if positive and i to j if negative, with weight $|A^*_{i,j} - A^*_{j,i}|$.

To find the time-lag dependent inter-region correlation shown in main text Fig. 5d, we repeated the method outlined above for a specific time-lag (x) such that, $\sum_{x-0.5s\leq\tau<x+0.5s} C^*_{i,j}(\tau)$, for x = +1, +3 and +5 s from which a directed graph is constructed. This was repeated for all cycle and temperature datasets for the "1L-4L" range. For all graphs found, the edges are filtered such that only 10 edges with highest weights are selected from each graph, and edges which occur in the majority of datasets are filtered for. The final graph is constructed from the filtered edges, and replotted on top of the particle regions for clarity. This is repeated for different selected time-lags, producing the figure in main text Fig. 4d.

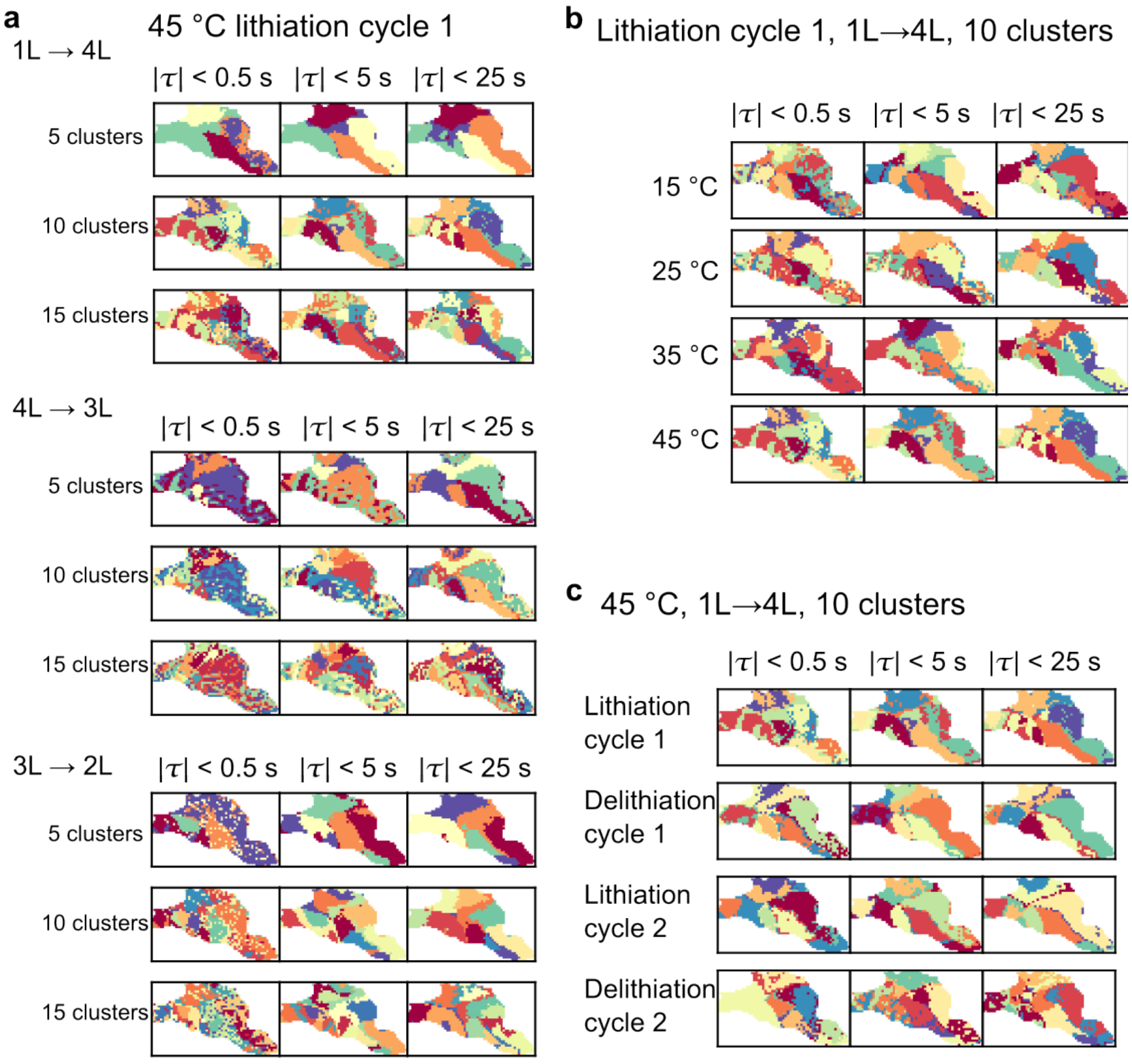

Figure S29 | Spectral co-clustered particle regions with different parameters. a,b,c Particle regions are found by calculating the cross correlation matrix ($\sum_{|\tau|<k} C_{i,j}(\tau)$) at various time lag sums (k = 0.5, 5, 25 s), spectral co-clustering given 5, 10, or 15 clusters. Results are shown for different electrochemical regimes (a), at different temperatures (b) and over different cycles and de/lithiation (c). Refer to the main text and supplementary methods for a detailed discussion.

3.3. Avalanche detection algorithm

A custom python script was written for avalanche detection, and an example of the process is shown in Figure S30. For the mean intensity trace (left panel, black line) for each masked region, a moving window median with size 15 (7.5 s) was applied to the time axis as an edge preserving filter (left panel, blue line) prior to calculated the mean from which the differential was calculated ((i+1)th – ith frame, right panel, black line). The differential signal was smoothed with a moving window average of size = 5 (2.5 s) (right panel, blue line) and the time points were classified as an avalanche if the absolute value of the differential signal exceeded a threshold (cutoff = 0.0004). The start, end points and change in intensity were collected from every segment satisfying these criteria (left panel, light black, right panel, light red). Very small avalanches with net intensity change smaller than 0.2% of the noise floor were ignored, where the noise floor is defined as the standard deviation of the mean intensity in the initial 100 frames where the intensity does not change (or for delithiation cycles, the final 100 frame). The accepted events and associated values were stored in a dataframe for plotting.

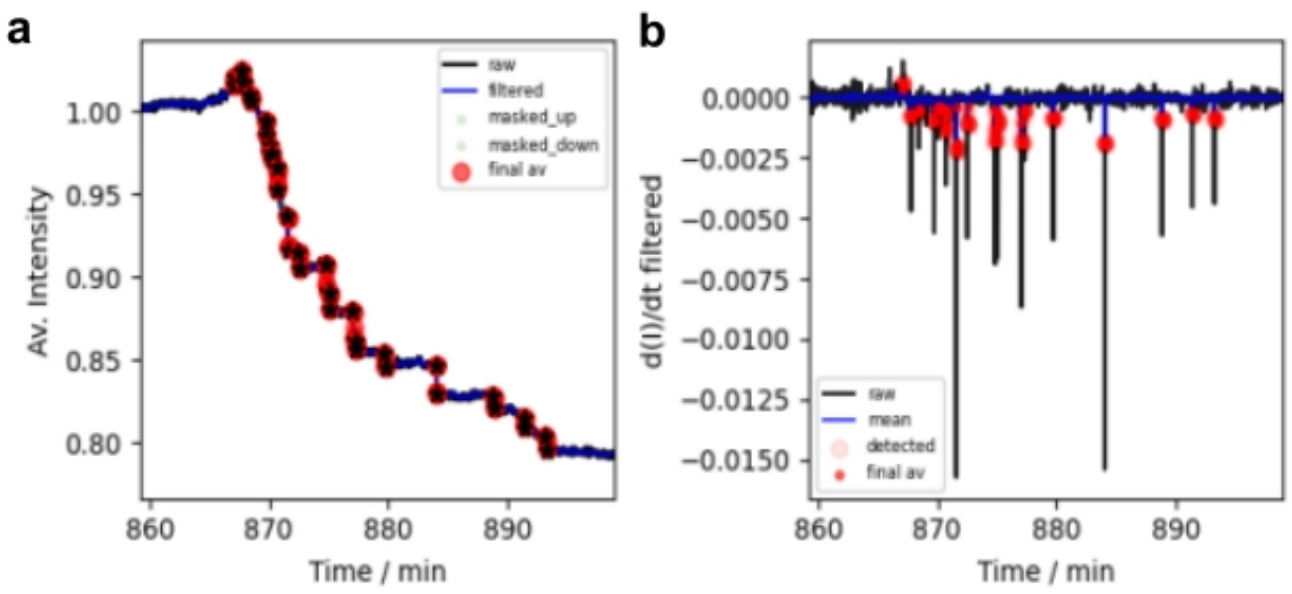

Figure S30 | Avalanche detection schematic a, Intensity trace used for avalanche detection shown in black (raw) and blue (after median filtering). Detected avalanche data points are coloured in red, with the start and end points marked with star. **b,** The differential intensity used for the avalanche detection. Black trace follows the differential as calculated from the median filtered signal, the blue trace follows the moving window mean and the red dots are drawn where the threshold is exceeded.

Section 4: Supplementary Figures

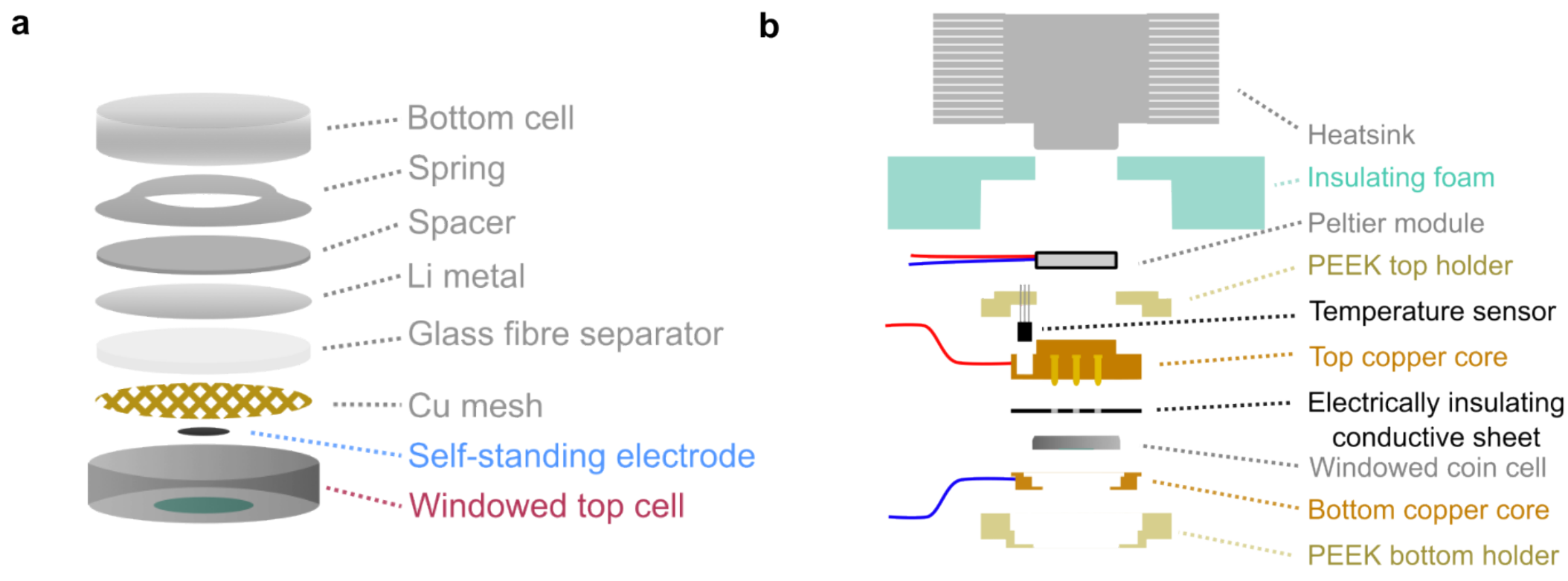

Figure S31 | Apparatus schematic. **a,** Schematic of the optical coin cell geometry used. **b**, Schematic of the custom made temperature controlled holder.

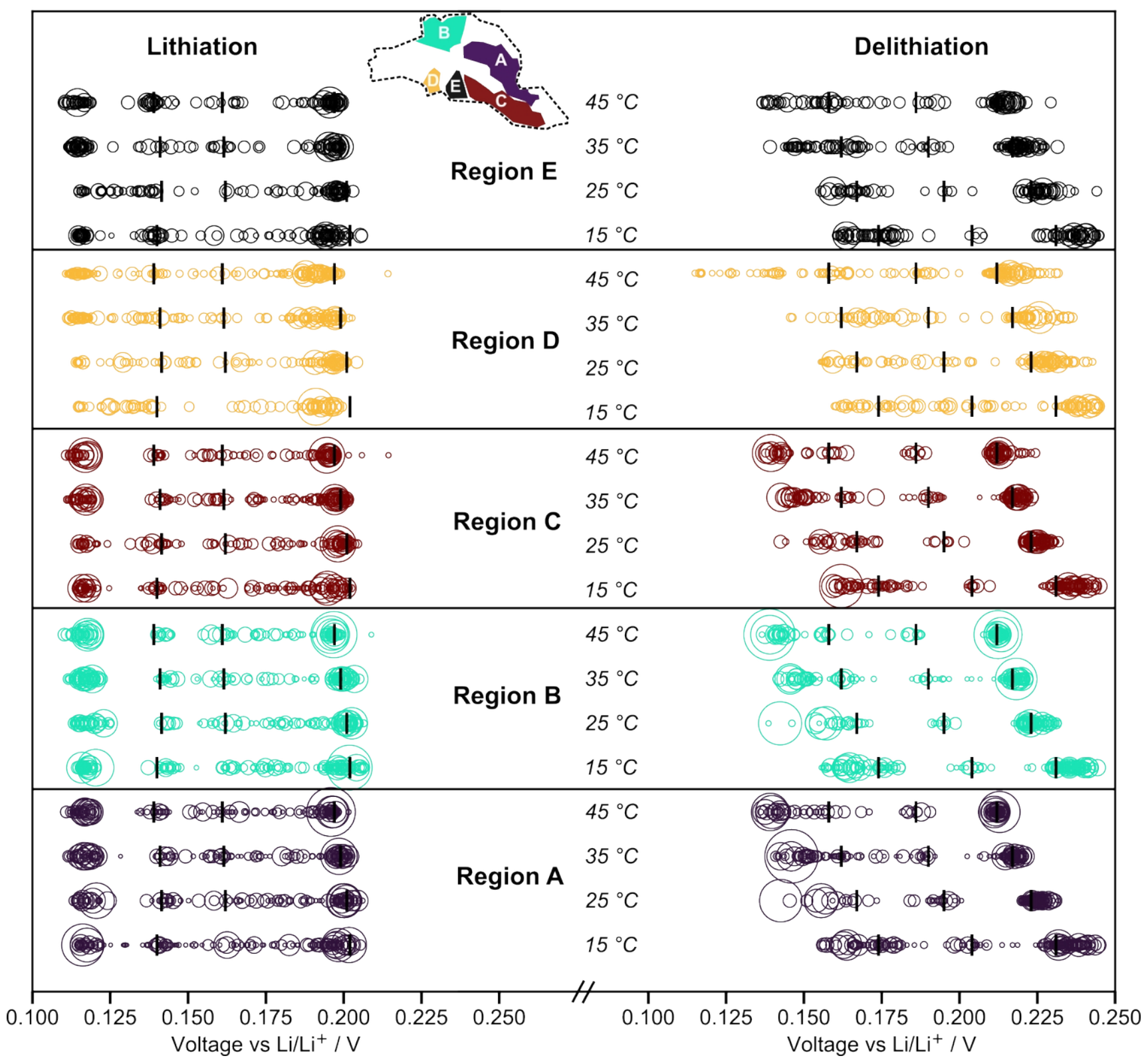

Figure S32 | Full experimental avalanche scatter plots. a,b, Scatter plot of the avalanches that occur during delithiation (right panel) or lithiation (left panel), detected from the normalised mean optical intensities in regions A-E, in cycles 1 and 2, as a function of temperature. Each circle represents one avalanche event, with its x-position denoting the voltage at which it occurs and the size denoting the change in intensity. Region is indicated in particle map shown in upper middle. A black vertical line indicates the peak position of the processes 1, 2a, and 3 denoted in main text Figure 2.

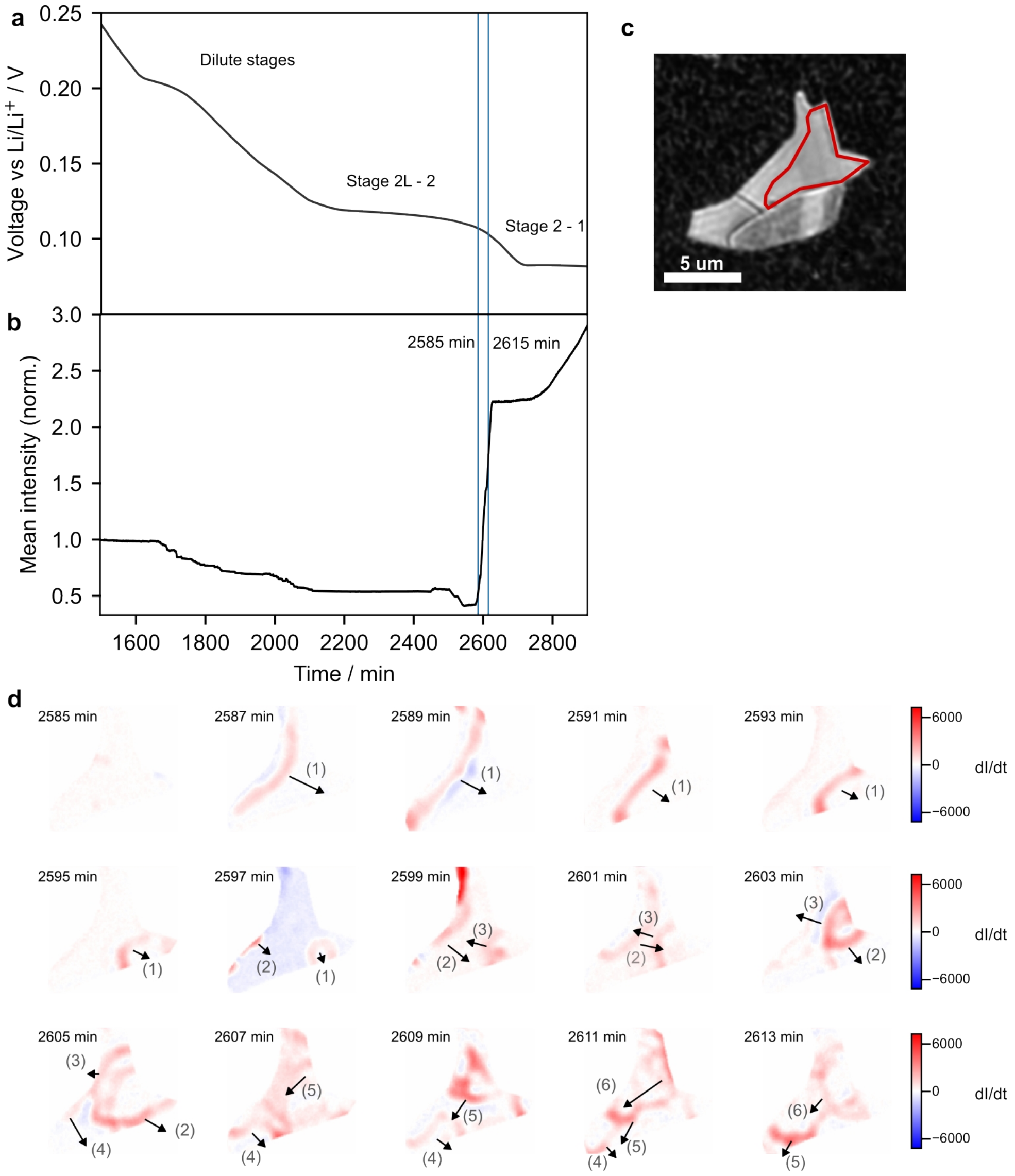

Figure S33 | Stage 2L to 2 transition in Kish graphite. a, The voltage profile during formation cycle at C/20 current rate, during lithiation on the cell shown in Supplementary Fig. S7a,d. Approximate transitions are denoted above the voltage curves. The time of interest between 2585 – 2615 min, highlighted with vertical blue lines, lie near the end of the Stage 2L to 2 transition. **b,** The mean intensity from the particle region (red highlighted region in (**c**)) during cycling, normalised to the start of lithiation. The particle undergoes the Stage 2L-2 transition near the end of the plateau, as highlighted with the blue vertical lines. **c,** The imaged particle (Kish exfoliated graphite), with the region of interest highlighted in red. **d,** The sequential differential images of the region of interest from the particle during a part of the Stage 2L-2 transition. The images show multiple phase fronts propagating through the particle region. The start time as well as nucleation point differ between phase fronts. A selection of these are denoted with a numbered arrow. The images are obtained by subtracting images 30 s apart with 3 x 3 pixel binning. Red (blue) indicate an instantaneous increase (decrease) in the local optical intensity.

Section 5: References

Badr, H. O., & Barsoum, M. W. (2023). On ripplocations and the deformation of graphite. *Carbon*, *201*, 599–615. https://doi.org/10.1016/j.carbon.2022.09.042

Baró, J., Planes, A., & Vives, E. (2016). Avalanche dynamics in driven materials. *CONTRIBUTIONS to SCIENCE*, (11), 153–162. https://doi.org/10.2436/20.7010.01.226

Chandesris, M., Caliste, D., Jamet, D., & Pochet, P. (2019). Thermodynamics and Related Kinetics of Staging in Intercalation Compounds. *The Journal of Physical Chemistry C*, *123*(38), 23711–23720. https://doi.org/10.1021/acs.jpcc.9b05298

Chen, T., Yang, J., Barroso-Luque, L., & Ceder, G. (2023). Removing the Two-Phase Transition in Spinel $LiMn_2$ O_4 through Cation Disorder. *ACS Energy Letters*, *8*(1), 314–319. https://doi.org/10.1021/acsenergylett.2c02141

Chen, Y., Chen, K.-H., Sanchez, A. J., Kazyak, E., Goel, V., Gorlin, Y., Christensen, J., Thornton, K., & Dasgupta, N. P. (2021). *Operando* video microscopy of Li plating and re-intercalation on graphite anodes during fast charging. *Journal of Materials Chemistry A*, *9*(41), 23522–23536. https://doi.org/10.1039/D1TA06023F

Dahn, J. R., Fong, R., & Spoon, M. J. (1990). Suppression of staging in lithium-intercalated carbon by disorder in the host. *Physical Review B*, *42*(10), 6424–6432. https://doi.org/10.1103/PhysRevB.42.6424

Dhillon, I. S. (2001). Co-clustering documents and words using bipartite spectral graph partitioning. *Proceedings of the Seventh ACM SIGKDD International Conference on Knowledge Discovery and Data Mining*, 269–274.

Dresselhaus, M. S., & Dresselhaus, G. (2002). Intercalation compounds of graphite. *Advances in Physics*, *51*(1), 1–186. https://doi.org/10.1080/00018730110113644

Durin, G., & Zapperi, S. (2004). *The Barkhausen effect* (arXiv:cond-Mat/0404512). arXiv. http://arxiv.org/abs/cond-mat/0404512

Kuntz, M. C., Perkovic, O., Dahmen, K. A., Roberts, B. W., & Sethna, J. P. (1999). Hysteresis, Avalanches, and Noise: Numerical Methods. *Computing in Science & Engineering*, *1*(4), 73–81. https://doi.org/10.1109/5992.774844

Mercer, M. P., Peng, C., Soares, C., Hoster, H. E., & Kramer, D. (2021). Voltage hysteresis during lithiation/delithiation of graphite associated with meta-stable carbon stackings. *Journal of Materials Chemistry A*, *9*(1), 492–504. https://doi.org/10.1039/D0TA10403E

Ohzuku, T., Iwakoshi, Y., & Sawai, K. (1993). Formation of Lithium-Graphite Intercalation Compounds in Nonaqueous Electrolytes and Their Application as a Negative Electrode for a Lithium Ion (Shuttlecock) Cell. *Journal of The Electrochemical Society*, *140*(9), 2490–2498. https://doi.org/10.1149/1.2220849

Perković, O., Dahmen, K., & Sethna, J. P. (1995). Avalanches, Barkhausen Noise, and Plain Old Criticality. *Physical Review Letters*, *75*(24), 4528–4531. https://doi.org/10.1103/PhysRevLett.75.4528

Persson, K., Sethuraman, V. A., Hardwick, L. J., Hinuma, Y., Meng, Y. S., Van Der Ven, A., Srinivasan, V., Kostecki, R., & Ceder, G. (2010). Lithium Diffusion in Graphitic Carbon. *The Journal of Physical Chemistry Letters*, *1*(8), 1176–1180. https://doi.org/10.1021/jz100188d

Schon, J. C., Adler, D., & Dresselhaus, G. (1988). Theory of staging in intercalation compounds. *Journal of Physics C: Solid State Physics*, *21*(33), 5595–5614. https://doi.org/10.1088/0022-3719/21/33/005

Sethna, J. P., Dahmen, K. A., & Myers, C. R. (2001). Crackling noise. *Nature*, *410*(6825), 242–250. https://doi.org/10.1038/35065675

Sethna, J. P., Dahmen, K., Kartha, S., Krumhansl, J. A., Roberts, B. W., & Shore, J. D. (1993). Hysteresis and hierarchies: Dynamics of disorder-driven first-order phase transformations. *Physical Review Letters*, *70*(21), 3347–3350. https://doi.org/10.1103/PhysRevLett.70.3347

Smith, R. B., Khoo, E., & Bazant, M. Z. (2017). Intercalation Kinetics in Multiphase-Layered Materials. *The Journal of Physical Chemistry C*, *121*(23), 12505–12523. https://doi.org/10.1021/acs.jpcc.7b00185

Song, B., Gu, H., Zhu, S., Jiang, H., Chen, X., Zhang, C., & Liu, S. (2018). Broadband optical properties of graphene and HOPG investigated by spectroscopic Mueller matrix ellipsometry. *Applied Surface Science*, *439*, 1079–1087.

Vives, E., & Planes, A. (1994). Avalanches in a fluctuationless first-order phase transition in a random-bond Ising model. *Physical Review B*, *50*(6), 3839–3848. https://doi.org/10.1103/PhysRevB.50.3839

Walsh, B., Austvold, S., & Proksch, R. (1998). Magnetic force microscopy of avalanche dynamics in magnetic media. *Journal of Applied Physics*, *84*(10), 5709–5714. https://doi.org/10.1063/1.368835

White, R. A., & Dahmen, K. A. (2003). Driving Rate Effects on Crackling Noise. *Physical Review Letters*, *91*(8), 085702. https://doi.org/10.1103/PhysRevLett.91.085702

Yao, L., & Jack, R. L. (2023). Thermal vestiges of avalanches in the driven random field Ising model. *Journal of Statistical Mechanics: Theory and Experiment*, *2023*(2), 023303. https://doi.org/10.1088/1742-5468/acb7ee

Zheng, T., Reimers, J. N., & Dahn, J. R. (1995). Effect of turbostratic disorder in graphitic carbon hosts on the intercalation of lithium. *Physical Review B*, *51*(2), 734–741. https://doi.org/10.1103/PhysRevB.51.734
