# Supplementary material for: Avalanche-like intercalation and intraparticle correlations in graphite
Source: Nature. 2026 Jul 29;656(8127):364–9. doi: 10.1038/s41586-026-10862-4 (PMC13468132; doi:10.1038/s41586-026-10862-4)
Supplement: Supplementary file 2 [file 41586_2026_10862_MOESM2_ESM.pdf]

nature portfolio

Peer Review File

Avalanche-like intercalation and intraparticle correlations in graphite

Corresponding Author: Professor Akshay Rao

Any redactions in this file are there to maintain patient confidentiality, the confidentiality of unpublished data, or to remove third-party material.

This file contains all reviewer reports in order by version, followed by all author rebuttals in order by version.

Version 0:

Reviewer comments:

Referee #1

(Remarks to the Author)
This reviewer would like to raise the following concerns and questions before this manuscript can be considered for publication in the Nature journal:

• Contrary to similar studies of Li-ion cathode materials, which exhibit much lower optical density, the light penetration depth in LixC is limited to surface regions, that is, less than a few tens of nanometers. On the other hand, the electrochemical potential response is largely determined by the behavior of the LixC bulk. It is unclear how the observed changes in local optical reflectivity at the particle surface can be extrapolated to the behavior of the material's bulk.
• For almost all graphite donor intercalation compounds, the optical reflectivity is typical of metallic free carrier absorption, that is, optical reflectivity increases with the concentration of the donor ion. Please explain why the observed optical intensity of diluted LixC is lower than that of pristine graphite.
• The observed variation in lithiation/delithiation rates between different areas or so-called "grains" and its translation into different lattice structures may appear as a conceptual shortcut across three orders of magnitude in length scale. Although the authors have made a solid effort to characterize the surface structure of the particle, this reviewer finds it insufficient to justify the proposed "avalanche" model. How is the observed mosaic pattern related to the structure/morphology/topology of the graphite single flake? Is this flake a conglomerate of polycrystalline primary particles, which consist of small (10-20 nm) graphene domains? In other words, how do the authors envision ion and electron barriers and percolation networks in such a large particle (>10 micrometers)?
• The observed step-like intensity features are linked with avalanche-like changes in the order parameter between metastable states, that is, discrete lithium occupation patterns in the graphite lattice. This could be the case on the individual graphene domain level (approximately 10-30 nm), but it is difficult to assume that such an effect can extend across large polycrystalline domains (approximately 2-7 micrometers) as observed in this study.
• The authors briefly state that “…our model is an idealized representation of disorder, such as stacking faults and turbostratic disorder in graphite. In reality, as lithium diffuses into graphite, a layer opening barrier, grain boundary diffusion barrier, and shear stresses from AA to AB layer stacking will add to the complexity of disorder”. Considering the graphite lattice expansion upon Li+ intercalation, the likelihood of instant changes in local ion transport pathways and electronic percolation networks between "grains" is not negligible. This effect will also be enhanced at higher temperatures, as ion diffusion in solids is exponentially dependent on temperature. In fact, the incomprehensible time-lag dependent intra-particle correlations shown in Figure 5 could be consistent with such a mechanism based on mechanical stress (re)distribution.
• The authors do not elaborate or speculate on how this new mechanism of intercalation in graphite is relevant to the performance of these types of electrodes in a more practical setting. This could motivate more interest in the mechanism and its investigation into other materials.
• Figure 1 plots c d and e have the x axis as time in (min). This could be converted into the expected composition of LixC based on the constant current and mass of the electrode. A compositional dependence in the graphite would provide a better connection to what composition these avalanches are occurring, rather than the arbitrary time of the electrochemical experiment.
• The experiments are conducted at C/20. Is the avalanche behavior rate dependent?
• There is no need to specify the unnormalized current (7 uA). Saying C/20 is sufficient or at least the mA/g or mA/cm2 to be

more specific.

In summary, this reviewer fully recognizes the novelty and value of the experimental results presented in this study. However, the proposed data interpretation and conclusions appear to be insufficiently documented and/or confronted with alternative mechanisms to ensure that the proposed methodology and discussion are sound and acceptable.

Referee #2

(Remarks to the Author)
I co-reviewed this manuscript with one of the reviewers who provided the listed reports.

Referee #3

(Remarks to the Author)
For all the technical mastery and inventiveness demonstrated in the manuscript, I believe it requires another round of careful review.

My main concern is that no avalanche behaviors have been demonstrated, contrary to the claims made in the title, abstract, and elsewhere in the manuscript. I appreciate the effort to introduce more physics and rigor into the field; however, when aiming for rigor, the work is held to a higher standard, and I do not believe the manuscript meets it.

The manuscript uses inappropriate terminology and analogies and does not convincingly support its claims. Not every abrupt transition should be labeled as an avalanche, and what is observed does not resemble avalanche behavior to me. The authors themselves provide damning evidence: the observed temperature dependencies are inconsistent with avalanching, and there are no signatures of amplification, which are present in related systems. It appears the authors may have been carried away by their analogies.

I suggest the manuscript remove these analogies and focus on what is actually observed: stepping and interdomain correlations. The Glauber-like Ising model introduced does not prove or disprove any particular point. It seems more like a qualitative exercise, yielding the behaviors it was designed to produce. While these behaviors may be new to battery scientists and chemists, they are not novel to physicists or materials scientists.

The nature of graphite lithiation is inherently complex, involving a succession of quasi–solid-solution and first-order transitions with phase coexistence. The manuscript's claim in the Introduction that the structural and dynamic aspects of this process remain poorly understood is, in fact, an understatement. As Timofeev-Resovsky once said, we have "progressed from false knowledge to true misunderstanding."

Grey and co-authors aim to demonstrate rapid, "avalanche-like" phase transitions during the initial stages of Li intercalation into graphite, which are traditionally viewed as disordered solid solutions. However, I am not convinced that "avalanche" is the appropriate term. These are relatively fast transitions, observed with a modest 500 ms time resolution. In physics, the term "avalanche" typically implies a snowballing effect with amplification, such as in avalanche photodiodes (impact ionization). I see no evidence for such amplification in the data. In other contexts — avalanche upconversion in optics, Townsend discharges, superconducting flux avalanches — avalanches always involve multiplication or amplification of particles, excitations, or correlations.

The manuscript invokes Barkhausen noise, which is sometimes called a magnetic avalanche. This phenomenon is indeed a collective cascade of domain-wall motion: unpinning events release stress, destabilizing neighboring regions, producing a snowballing effect. Signatures of such amplification include crackling noise, with power-law distributions in amplitude, duration, and power spectrum. Similar principles apply to ferroelectric avalanches, historically called polarization Barkhausen noise.

The authors have not demonstrated such characteristics typical of critical phenomena involving amplification. Without these demonstrations, they should not use terminology reserved for phenomena with proven amplification mechanisms. The observed phase transitions may or may not be avalanche-like, but this still requires rigorous proof.

In particular, the random-field Ising model presented does not justify the use of avalanche terminology. It is not a proper dynamic model; it is a Glauber-like model for thermally activated metastable transitions. It imposes periodic boundary conditions, ignores Li diffusion, and lacks domain walls and defects. Its main purpose appears to be incorporating interlayer interactions. The statistics observed in the model (Figure S9) bear no resemblance to the experimental avalanche systems. By design, this type of model produces stepping once thermal excitation exceeds local barriers — which is intrinsic to the model and requires no computation. In this sense, the model is not instructive.

It is especially concerning that, as the authors themselves note on p. 12, the observed temperature behavior is inconsistent with expectations from Glauber-type models. Explaining this discrepancy as a deficiency in the detection algorithm is not sufficient. A model alone cannot establish similarity to avalanches known in other systems involving defect pinning at domain walls; it remains purely speculative.

A more compelling part of the manuscript is the spatio-temporal correlation between domains, analyzed using a graph representation of domain boundaries. The data convincingly show correlations between stepping in adjacent domains.

However, these correlations do not prove an avalanche mechanism or validate the model. They suggest varying degrees of interdomain connectivity without clarifying its origin. This is disappointing, as extensive effort in establishing correlations yields limited insight.

Referee #4

(Remarks to the Author)
The work by Han et al. studies the emergent dynamics of ion insertion and extraction in graphite particles, one of the most relevant anode materials used in Li-ion batteries. The authors use operando optical scattering microscopy coupled with classical electrochemical measurements to unravel the behavior of graphite under ion (de)intercalation for filling fractions up to 50%. Their objective is to reveal the emergent phase transitions rooted in the ion--ion interactions that coexist within and between neighboring layers of graphite in the dilute regime, a region that remains poorly understood even today. Their optical signal measurements reveal the existence of an 'avalanche'-like behavior in the dilute regime (0.25 to ~0.10 V vs Li/Li^+) when graphite is cycled under a C/20 rate and different temperatures (15 oC, 25 oC, 35 oC, and 45 oC). They attribute this behavior to the inherent quenched disorder present within the graphite structure. To test this hypothesis, they performed numerical Monte Carlo calculations based on a model Hamiltonian that describes nearest and next-nearest neighbor interactions between spins (occupied and unoccupied sites), which demonstrated very similar phenomenology to what the experiments showed. Lastly, the authors close their article with a correlation map across the single graphite particle they studied, demonstrating how specific regions undergo correlated ion insertion and extraction.

Overall, this work is interesting as it introduces a perspective grounded in fundamental physical chemistry and physics to one of the most classical materials used in Li-ion batteries. It also advances the understanding of graphite as an intercalation material in a regime that is rarely explored with such characterization methods, largely due to the inherent difficulty of performing in-operando measurements. I personally enjoyed the ideas behind this work, though one could argue that the observations and phenomenology presented here are limited to a single material. In addition to these comments, I have more specific questions and suggestions for the authors regarding their work. These are:

1. I find it necessary to pursue experiments at both much lower and higher C-rates, ideally below the diffusion-limited current in these graphite systems. This would clarify how equilibrium and non-equilibrium factors influence the observed behavior and whether non-equilibrium forcing smooths out the discrete jumps seen in the optical measurements.

2. The authors appear to have focused their analysis and conclusions on measurements from a single particle. I assume that these observations were reproduced in multiple samples, and it would therefore be beneficial to demonstrate consistency across several particles. While I understand this may require significant effort, such validation would strengthen the claim that the observed behavior is intrinsic to graphite and not specific to one experimental sample.

3. In the experiments, the filling fractions start and end at the same point, indicating that the endpoint for lithiation matches the starting point for delithiation. However, in the simulations, this is not the case. Why? I can imagine this might arise if each simulation begins from a newly sampled initial configuration. If so, the simulations do not fully mirror the experimental protocol, which may explain the observed hysteresis.

On a related note, there is a clear voltage jump between the end of lithiation and the start of delithiation, indicating a reaction overpotential, especially at lower temperatures. It would therefore be valuable to show that at lower applied C-rates, this overpotential decreases and the material approaches quasi-static lithiation and delithiation. This would effectively decouple thermodynamic from kinetic effects, which I believe aligns with the authors's broader message.

4. The argument: 'However note that the step-detection algorithm is prone to detecting multiple step events as a single large event as the time separation between steps decreases. Therefore, at higher temperature as the events bunch more in time (see Fig. 2b), clusters of smaller events may frequently be detected as a single large avalanche, resulting in deviation from the trend,' appears rather weak in explaining the disconnect between the phenomenological simulation model and the experimental results. I would suggest a more comprehensive investigation into why the model fails to capture the experimental trends.

5. The current modeling approach appears very simple. While I appreciate the value of simplicity in a complex system such as ion intercalation materials, the present model is quite far from representing the structure of graphite. Specifically, the model is purely two-dimensional, with quenched disorder only in the yz-directions, completely neglecting the emergence of nucleated phases in the xy-plane, where surface tension effects are expected to play an important role. Additionally, the disorder is defined as site-specific rather than expressed through variations in the coupling constants between layers, which could lead to qualitatively similar behavior.

It would strengthen the work if the authors perform a more comprehensive analysis regarding system size effects, as the current model is too small to draw conclusions that can meaningfully compare to experiments. Given the standards of a Nature publication, I would expect a much more extensive computational study that goes beyond a simple 2D Ising-like model to include 3D systems and, ideally, extensions toward phase-field-type models. Such an effort would connect the present work to two decades of literature on graphite modeling and provide a more complete physical picture. Lastly, it was never mentioned how the authors chose the rate at which they varied the baseline chemical potential in their model.

6. In connection to comment (5) and the last paragraph of the introduction:

'Finally, using the cross-correlation function as an effective way of analysing the stochastic filling events, we demonstrate that the seemingly random sequence of avalanches revealed in our optical experiments possess definite spatio-temporal correlations patterns, related to the regions and disorder identified in the electron backscatter diffraction (EBSD) map.'

This finding provides a strong motivation for pursuing a more comprehensive computational analysis to understand how lithiation and delithiation progress in quenched disordered systems, rather than limiting the discussion to the correlation level of experimental observations.

Minor comments

1. There are several typos in the SI of the paper. I would suggest a more careful proofreading of the submitted material. It would also be very helpful to include a complete reference list in the SI, as several papers are not properly cited and I had to infer which works the authors refer to.

2. It would be great to quantify the ion fractions corresponding to the dilute regime, both in the introductory paragraph and throughout the text. This would help readers directly relate the observed phenomena to the corresponding stored energy.

3. In the sentence 'Phase transitions in battery materials are often categorized as a solid-solution 12,13 or phase separating 14--17, where lithiation in graphite has been reported to have both first-order biphasic transitions and 'pseudo' solid-solutions 9,18,19,' I have two comments:
i) I do not believe this phrasing is precise. How can a phase transition itself be categorized as a solid solution?
ii) I do not think the cited works explicitly establish the terminology of 'pseudo solid-solution,' so it is a bit difficult to interpret in the broader context of phase transitions.

4. In the sentence 'This is fundamentally different from our previous optical microscopy studies where we observed first-order biphasic behaviour 24 or solid-solution diffusion 25,26 within cathode materials,' it would be helpful to explicitly mention the specific chemistries studied previously.

5. In Fig. 3b, it would be useful to include an explicit schematic illustrating the definition of the inter-gallery distance. It took me some time to distinguish the white lines and understand the coloring. Moving the schematic from Extended Data Fig. 2a into Fig. 3b would greatly improve clarity for readers.

Version 1:

Reviewer comments:

Referee #1

(Remarks to the Author)
While I still find some of the points made by the authors in the manuscript and their responses to the reviewers somewhat debatable, I am satisfied with the clear statements regarding the limitations of their experimental approach, observations, theoretical extrapolations, and conclusions. High-quality scientific papers benefit from a dedicated 'Limitations' section; this manuscript is no exception, it would help turn inherent uncertainties into a strength. Considering this broader context, I find the paper acceptable for publication.

Referee #2

(Remarks to the Author)
I co-reviewed this manuscript with one of the reviewers who provided the listed reports.

Referee #3

(Remarks to the Author)
I was Reviewer 3 of the original submission. First, I would like to thank the authors for their detailed and thoughtful replies, as well as for the additional work undertaken in response to the reviews. I continue to regard this as an excellent paper. I also suspect that some of the remaining controversies would ultimately be better addressed through broader communal scrutiny than through further debate within the confines of journal review.

I am pleased that the authors have revised the manuscript to make more careful claims regarding their observations and to clarify several points that concerned both myself and the other reviewers. However, this improvement has come at a certain cost: the paper has become rather bloated and, in places, difficult to read. With respect, the theoretical model still appears only tenuously connected to the experimental section and, in my view, does not meaningfully strengthen it. The manuscript now reads somewhat like an enforced union of two papers, one of which would seem entirely deserving of publication in Nature, while the other perhaps less so. I wonder whether a separation might ultimately serve the study better.

In its present form, the experimental and theoretical components appear to belong to rather different categories in terms of novelty, achievement, excitement, and rigour. It is also difficult not to notice, from the tenor of the reviewers’ comments,

which part of the manuscript is responsible for most of the contention.

For this reason, I would suggest removing the section entitled “Phase transition behavior of a disordered system…” and focusing the present manuscript more squarely on the phenomenology and qualitative aspects of the observations. The theoretical material could then be developed into a separate and more coherent paper, drawing together the relevant material currently dispersed between the main text and the Supplementary Information, supplemented with the experimental results where appropriate, and submitted to a journal more suited to that discussion.

At present the theoretical section is compressed to such a degree that the narrative feels rushed and depends heavily on the Supplementary Information. Many readers will likely not pursue that material, particularly as a large fraction of the readership will not be physicists. Even for a specialist, the argument is not always straightforward to follow in its current form.
Having read the other reports, I am also not entirely convinced that review of this rather technical physics section by battery scientists is proving particularly effective. It would benefit, in my opinion, from evaluation by experts in the specific modelling approaches being employed and their relation to the current state of the art. Such a discussion would be more appropriately considered as a self-contained theoretical manuscript rather than as an adjunct to the present experimental study.

My concern is that the theoretical section may overwhelm and distract the general reader from what is, in my view, the genuine and uncontroversial novelty of the work: the careful and masterful experimental observations and their unification with other such behaviors observed in other contexts.

I offer these comments in a constructive spirit. I fully appreciate that the authors on the theoretical side will not share this view. Nevertheless, if they are as confident in their explanations as their rebuttals suggest, I wonder whether separating the two strands might ultimately prove advantageous. Instead of a single, overextended manuscript, the result might well be two strong papers.

As regards the experimental sections, now that the more problematic claims have been moderated and clearer delineations introduced, I would have no objection to publication in Nature. With respect to the theoretical parts, however, I would prefer not to enter into a detailed debate regarding the finer points of the modelling or the resolution of the associated controversies. In my view, a striking experimental paper reporting new phenomena does not require a hurried theoretical framework, particularly where claims of understanding remain provisional. Indeed, even over the course of this review process, some of the proposed explanations have already evolved rather substantially while the confident tone remained unchanged.

Referee #4

(Remarks to the Author)

Open Access This Peer Review File is licensed under a Creative Commons Attribution 4.0 International License, which permits use, sharing, adaptation, distribution and reproduction in any medium or format, as long as you give appropriate

credit to the original author(s) and the source, provide a link to the Creative Commons license, and indicate if changes were made.
In cases where reviewers are anonymous, credit should be given to 'Anonymous Referee' and the source.
The images or other third party material in this Peer Review File are included in the article's Creative Commons license, unless indicated otherwise in a credit line to the material. If material is not included in the article's Creative Commons license and your intended use is not permitted by statutory regulation or exceeds the permitted use, you will need to obtain permission directly from the copyright holder.
To view a copy of this license, visit https://creativecommons.org/licenses/by/4.0/

Response Letter

Dear reviewers:

We genuinely thank all reviewers for their interest in our manuscript and the time spent providing detailed and valuable comments. We have made changes to the manuscript and restructured Supplementary Information. New additions and changes to the manuscript that have been made in response to the reviewers is highlighted in blue. Not all changes made to the Main manuscript and Supplementary are included here (blue) so as to focus on the key changes - please refer to the full document for more details.

Reviewer #1 :

This reviewer would like to raise the following concerns and questions before this manuscript can be considered for publication in the Nature journal:

We thank the reviewer for their endorsement of the novelty and their interest in the study overall. We provide a point-by-point response below.

R1.1: *Contrary to similar studies of Li-ion cathode materials, which exhibit much lower optical density, the light penetration depth in LixC is limited to surface regions, that is, less than a few tens of nanometers. On the other hand, the electrochemical potential response is largely determined by the behavior of the LixC bulk. It is unclear how the observed changes in local optical reflectivity at the particle surface can be extrapolated to the behavior of the material's bulk.*

We acknowledge that the penetration depth of Li-GIC in the visible is limited to the sub/surface of the particle, and that the overall lithiation state of a large, single crystalline particle may differ from that of the surface. However, it is import to stress that this work is focused on the study of intercalation dynamics in smaller, thinner particles (c-axis crystallite size (L_c) $\gtrapprox$ 100 nm, Timcal Timrex KS44), and in particular, the demonstration of step-like intercalation events as a new phenomenon in the dilute stages. For this, we believe that the penetration depth (40-45 nm) is sufficient to describe the overall dynamic system, and the results we observe are not only limited to the surface. We have included Supplementary Section 1.3.2 to explain this:

Supplementary 1.3.2: Surface vs bulk intensity contributions

At λ = 730 nm, the penetration depth ($d_p = \lambda/4\pi k$) for pristine graphite is ~45 nm, or ~140 layers (Song et al., 2018). At the end of the dilute stages (x = 0.22), the penetration depth slightly decreases to ~40 nm, or ~120 layers. The graphite used in this work (Timcal Timrex KS44) has a has a reported c-axis crystallite size (L_c) of >100 nm. Since the domain size of the graphite in the c-axis is similar to the penetration depth of our technique, our experimental observations can be attributed to that of the bulk graphite response. Critically, by probing more than 100 layers we ensure that we (1) fully describe the dynamics of step-like intercalation and (2) overcome the concern that the step-like intercalation process is only a surface effect.

R1.2: *For almost all graphite donor intercalation compounds, the optical reflectivity is typical of metallic free carrier absorption, that is, optical reflectivity increases with the concentration of the donor ion. Please explain why the observed optical intensity of diluted LixC is lower than that of pristine graphite.*

The reviewer is correct in that, generally, reflectivity is expected to increase with lithiation (and corresponding increase in carrier density) past the plasma frequency according to the well established Drude Model, due to the onset of metal-like reflection. Interestingly, the onset of metal-like behaviour is wavelength dependent, and for a given wavelength of light, prior to metallic reflection onset, we predict a drop in reflectivity. To explore this in more detail, we have modelled the reflectivity drop as well as the overall optical response at 730 nm, in Supplementary Section 1.3.1:

Supplementary 1.3.1: Optical response of lithiated graphite

The optical reflectivity of graphite intercalation compounds (GICs) can be described by the Drude free carrier model (Dresselhaus & Dresselhaus, 2002). Briefly, in the Drude model, the dielectric function is approximated by its core dielectric with its free carrier contribution:

$$\varepsilon(\omega) = \varepsilon_\infty - \frac{\omega_p^2}{\omega^2 + i\gamma\omega},$$

Where ε_∞ is the core dielectric, γ is the damping constant and the ω_p is the plasma frequency, given by $\omega_p = \sqrt{\frac{Ne^2}{\varepsilon_0 m^*}}$, where m^* is the carrier effective mass, ϵ_0 is the vacuum permittivity and N is the carrier density. We assume that lithiation results in a linear increase in carrier density with full ionisation, with lithiation x = 1 (LiC_6) for Stage 1 GIC, x = 0.5 (LiC_{12}) for Stage 2 GIC, and x = 0.22 (LiC_{27}) at the end of dilute stages or "Stage 2L" GIC (Ohzuku et al., 1993). Parameters used in the model can be found in Table S1.

As shown in Figure S9a for a wavelength of 700 nm, the Drude model predicts an initial near linear decrease in intensity during lithiation for the dilute stages, followed by an increase in intensity at the beginning of the dense stages. At high lithiation levels, the intensity change becomes less pronounced. This behaviour can be explained by the increase in carrier density for GICs changing plasma frequency. As the carrier density of graphite increases during lithiation, the plasma frequency shifts to lower wavelengths (higher energy) and eventually crosses the wavelength of the experiment. The Drude model predicts metallic reflections (i.e. reduced reflection) for wavelengths below the plasma frequency, which results in a non-monotonic intensity trend at 700 nm over the course of lithiation. This can be seen clearly by evaluating the spectral reflectivity profile shown in Figure S9b, which further illustrates this behaviour and connects the observation to the well-documented colours of Li-GICs, namely Stage 1 GIC (bright yellow), Stage 2 GIC (red), dilute stage GIC (grey-blue) as seen in previous studies(Y. Chen et al., 2021).

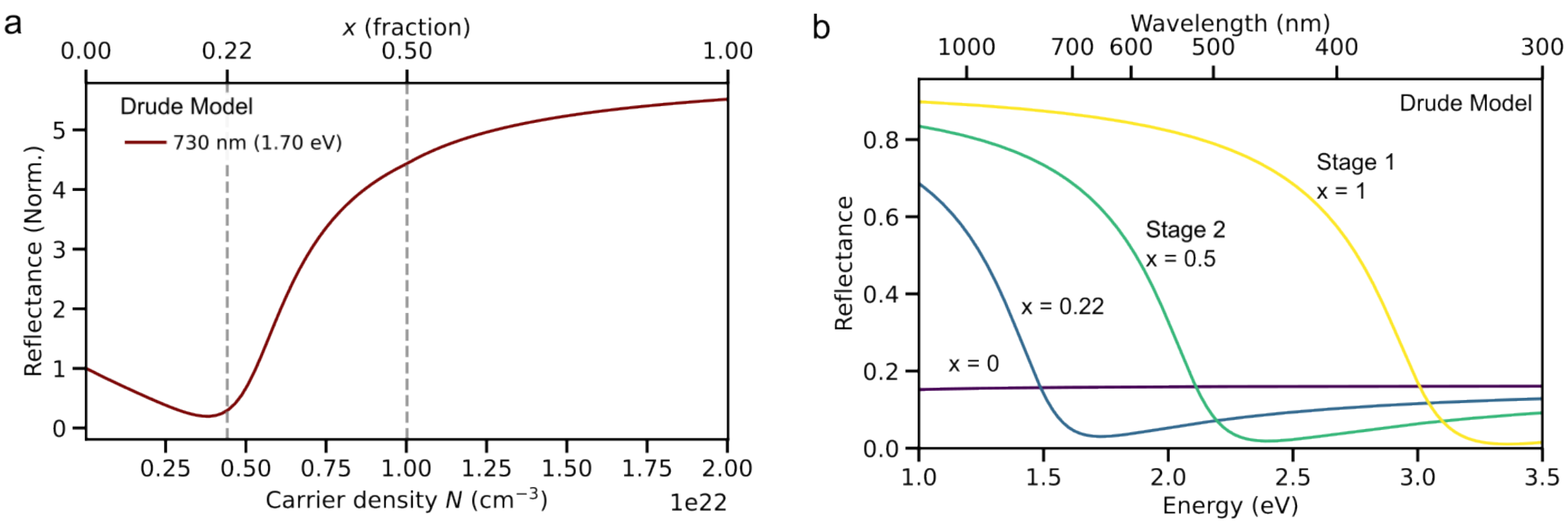

Figure S9 | Drude model of graphite reflectivity. a, Normalised reflectance of lithiated graphite at single wavelength (730 nm) according to the Drude model, as a function of lithiation (top axis) and resultant carrier density (bottom axis). Dotted vertical lines correspond to 22% lithiation (end of dilute stages) and 50% lithiation (Stage 2). **b,** The reflectance spectra vs wavelength, at various lithiation states according to the Drude model. x = 0, 0.22, 0.5, and 1 are selected for comparison.

Table S1 | Drude Model parameters

ε_∞	m^* (m_e)	N (cm^{-3})	γ (rad/s)
5.5	0.04 (graphite), 0.5 (Stage 1) (Dresselhaus & Dresselhaus, 2002; Williamson et al., 1965)	2×10^{19} (graphite), 2×10^{22} (Stage 1) (Dresselhaus & Dresselhaus, 2002)	5×10^{14}

R1.3: *The observed variation in lithiation/delithiation rates between different areas or so-called "grains" and its translation into different lattice structures may appear as a conceptual shortcut across three orders of magnitude in length scale. Although the authors have made a solid effort to characterize the surface structure of the particle, this reviewer finds it insufficient to justify the proposed "avalanche" model. How is the observed mosaic pattern related to the structure/morphology/topology of the graphite single flake? Is this flake a conglomerate of polycrystalline primary particles, which consist of small (10-20 nm) graphene domains? In other words, how do the authors envision ion and electron barriers and percolation networks in such a large particle (>10 micrometers)?*

We address this point fully together with point 4 below.

R1.4: *The observed step-like intensity features are linked with avalanche-like changes in the order parameter between metastable states, that is, discrete lithium occupation patterns in the graphite lattice. This could be the case on the individual graphene domain level (approximately 10-30 nm), but it is difficult to assume that such an effect can extend across large polycrystalline domains (approximately 2-7 micrometers) as observed in this study.*

We thank the reviewer for helping clarify the discussion on the particle morphology. We have studied the particle ex-situ in more detail, and in short, the graphite particle studied in the main text is not a conglomerate of polycrystalline particles. In order to clarify the particle morphology, disorder and its correlation with the avalanche "regions" as described in the paper, we have pursued a more detailed characterisation of the particle using AFM, EBSD and ex-situ SEM. We have added *Supplementary Section 1.1.1*, showcasing some of these results.

Secondly, we realise that the "region" labelling in the paper may have led to confusion and we apologise. We have termed "region" for the spatial areas in which avalanches tend to occur together. Each avalanche event varies broadly in its size and spatial extent, but there are clear, statistical tendencies to occur in each region (see *Supplementary Section 1.2.1* in response to R3.5 for more detail). In fact, we avoid describing the regions as "grains" as the whole particle can be considered a single grain. We also avoid calling them them "domains" to prevent confusion with Daumas-Herold islands known in literature, which are believed to ranges in scale from a few nm up to 1 μm (Kortan et al., 1982; Safran, 1987; Thomas et al., 1980). We have added a *Supplementary Section 1.1.2* discussing this in more detail.

Finally, we have made changes to the main text (in italics) to highlight this better:

Main:

Critically, *in Supplementary Section 1.1, we discuss the grain reference orientation deviation (GROD) map derived from ex-situ EBSD measurement and the particle topology map,* which demonstrate that these regions may be associated with *localised disorder related to elastic deformation*, with areas associated with region D (and E) showing a *higher density of disorder.*

Supplementary 1.1.1: Particle characterisation

The cell was disassembled post-cycling and the particle was characterised ex-situ. The scanning electron microscopy (SEM) image shows the single particle of graphite partially imbedded in the electrode (Fig. S1a). From electron backscatter diffraction (EBSD) measurements, the inverse pole figure map (Fig. S1c) together with pole figures of the (0001) and (10-10) lattice vectors (Fig S1d, (i) and (ii) respectively) demonstrate that the particle is a continuous single crystal of graphite presenting the basal plane (0001) as opposed to polycrystalline, nanodomain graphite (e.g. MCMB, see Fig S8a). There are some subtle bends and kinks on the particle, better shown in the side-on image (Fig. S1b), which can be quantified and mapped by analysing the grain reference orientation deviation (GROD) in the EBSD data (Fig. S1f). This misorientation is likely due to elastic deformation, rather than due to grain boundaries between polycrystalline grains. In addition, the overall misorientation is relatively small (< 4°), and in most parts, evolves smoothly. We extract the GROD magnitude along a line (Fig. S1f black line) and plot its change in Fig. S1g. The equivalent line is drawn on the image of the clustered regions (Fig. S1e), and the boundaries between expected cluster regions is overlayed on Fig. S1g (dotted lines, and numbers above the plot). The areas of constant GROD slope shows excellent agreement with the expected cluster regions. A constant

GROD slope indicates areas of constant elastic deformation, likely caused by bends and kinks in the particle.

To understand this further, we pursued atomical force microscopy (AFM) mapping of the particle. The AFM topology map (Fig. S1h) show the mostly smooth surface of the particle. Note that the scan line artifacts occur in the slow scan direction (x-direction), making the topology less reliable in the vertical direction. Similarly, an equivalent line is drawn on the topology map (Fig. S1h, black line) and the profile change along this is shown in Fig. S1i which clearly demonstrates the mostly flat particle surface separated bends and kinks. Again, the boundaries between expected cluster regions are indicated with vertical black lines. Overall, the bends and kinks are well correlated with the regions and the areas of constant GROD slope. For example the sharp kink at ~0.6 μm corresponds well to the sharp gradient between two areas of constant GROD slopes (Fig. S1g) near 0.6 – 0.8 μm, and the boundary between region 5 and 1 in Fig. S1e. Flake-type battery graphites are known to have such kinks, and examples of this in KS44 (studied in main text) and Hitachi MAGE3 graphite are shown in Fig. S8.

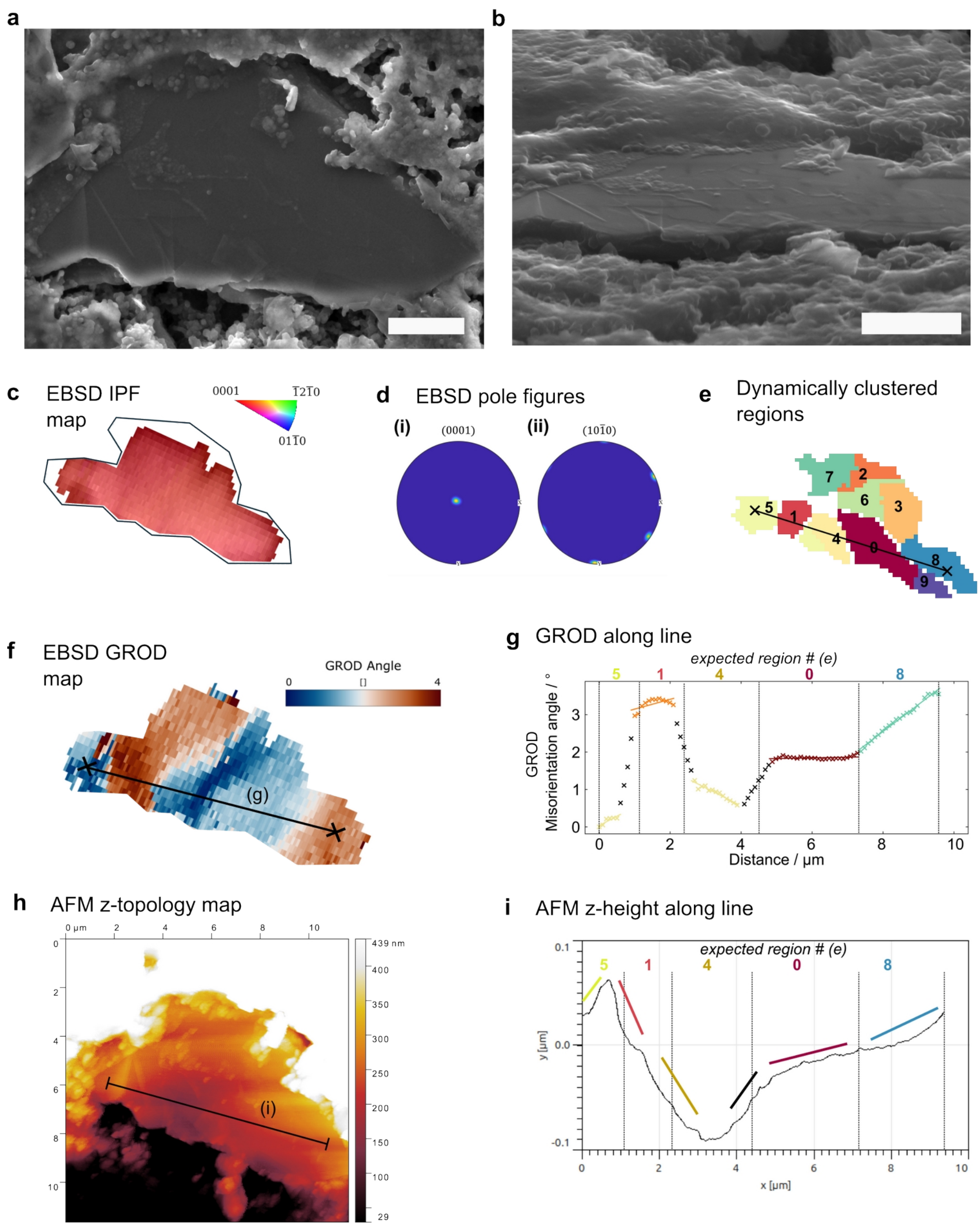

Figure S1 | EBSD, AFM and high-resolution SEM characterisation of graphite particle. a,b, Close up SEM images of the particle without tilt **(a)** and at 70° tilt **(b)**. Scale bar 2 µm. **c,** Electron backscatter diffraction (EBSD) map with inverse pole figure (IPF) color coding, measured from the ex-situ graphite particle in the electrode after cycling. The inverse pole figure (IPF) mapping colourcode is shown by the legend (top right). **d,** EBSD pole figures, integrated over the entire particle, for (0001) lattice vector **(i)**, and (01-10) lattice vector **(ii)**. **e,** Particle map showing clusters found through Spectral Coclustering method from the spatio-temporal correlation described in the main text Fig. 4. A white dotted line indicates the line of interest. **f,** The grain reference orientation deviation (GROD) map of the particle, showing the grain misorientation colourcoded as in legend (top right). A line of interest is drawn on top, in black. **g,** GROD misorientation value shown along the line of interest, with coloured numbers and vertical black dotted line indicating the location of expected cluster regions. **h,** Atomic force microscopy topology map of the particle imbedded in the electrode. The line of interest is drawn in black. **i,** The z-height profile along line of interest, with coloured numbers and vertical black dotted line indicating the location of expected cluster regions.

1.1.2: Disorder correlation with clustered regions

The ex-situ characterisation strongly suggests that disorder in the particle may originate from elastic deformation due to bends and kinks. This suggest that the clustered regions during intercalation (at least partially) originate from the kink boundaries and ripplocations disorder(Badr & Barsoum, 2023).
In fact, this suggests that the poor connectivity of clustered regions in the left side of the particle, identified via correlation methods (main text Fig. 5b), may be related to higher density of kinks and elastic disorder on the left side of the particle. In Fig S2 we show the local gradient norm on the particle calculated from the AFM z-topology map. This better highlights areas of constant curvature and location of kinks. Ignoring the horizontal scan line artifacts, the left side of the particle has more kinks and bends whereas the lower right side is flatter and continuous. This is in agreement to the connectivity correlation intensity sums (main text Fig. 5b) as well as the temporal distribution of avalanches (Supplementary Fig. S27), indicating that poor intraparticle connectivity and broadened temporal spread of avalanches occur in regions with higher elastic deformation.

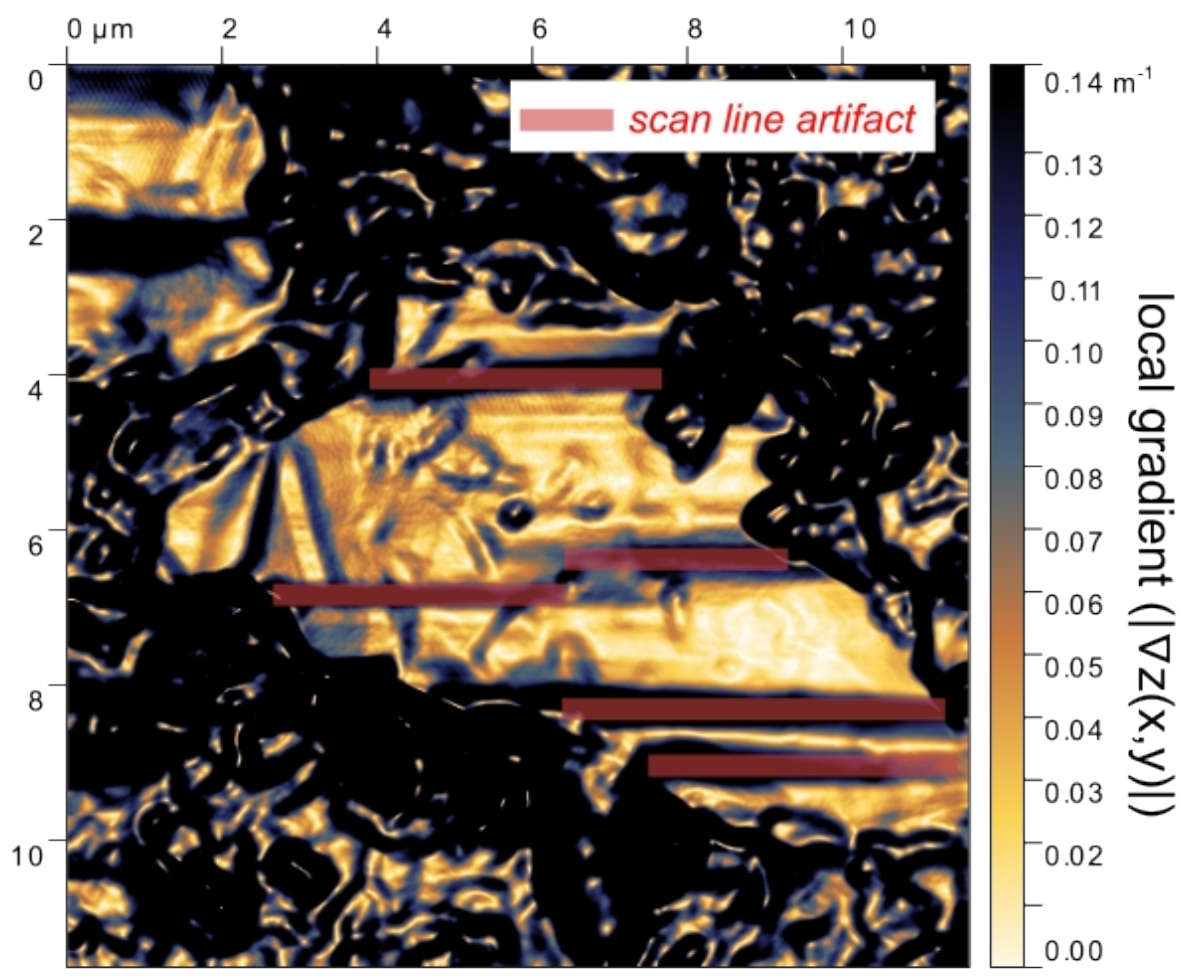

Figure S2 | Local gradient norm from AFM map. The local gradient, defined as the norm of the 2D gradient vector at each point is calculated. To account for surface roughness, a running window mean of 3 x 3 pixels (68 x 68 nm) is applied. Sharp gradients along exactly horizontal lines arise from scan line artifacts, and have been marked with a red square.

R1.5: *The authors briefly state that "…our model is an idealized representation of disorder, such as stacking faults and turbostratic disorder in graphite. In reality, as lithium diffuses into graphite, a layer opening barrier, grain boundary diffusion barrier, and shear stresses from AA to AB layer stacking will add to the complexity of disorder". Considering the graphite lattice expansion upon Li+ intercalation, the likelihood of instant changes in local ion transport pathways and electronic percolation networks between "grains" is not negligible. This effect will also be enhanced at higher temperatures, as ion diffusion in solids is exponentially dependent on temperature. In fact, the incomprehensible time-lag dependent intra-particle correlations shown in Figure 5 could be consistent with such a mechanism based on mechanical stress (re)distribution.*

We acknowledge that the idealised view of uncorrelated disorder used in the model is a simplification. As the reviewer mentions, mechanical stress and strain related to gallery filling is not represented in our modelling. We have modified the *main text* highlighting this aspect as follows:

We note that our model uses an idealised representation of quenched, uncorrelated disorder as a *proxy for static disorder (stacking faults, turbostratic disorder*[37] *and elastic deformation*[44]*).* In addition, the *Li itself can cause lattice deformations during intercalation with lattice expansion and stress distribution,* shear stresses associated with the change from AA to AB layer stacking[9], and layer opening, which will add to the complexity.

While we recognise that a more realistic structural simulations would be beneficial, such a study is challenging since these graphite samples include several different kinds of disordered features that span a range of length scales, and whose impact on the Li dynamics is hard to quantify. Hence our focus here is on a simple model that is sufficient to capture the qualitative behaviour. We discuss this further in *Supplementary Section 2.4* which can be found below in response to R1.10.

R1.6: *The authors do not elaborate or speculate on how this new mechanism of intercalation in graphite is relevant to the performance of these types of electrodes in a more practical setting. This could motivate more interest in the mechanism and its investigation into other materials.*

We thank the reviewers for helping raise relevance of our manuscript. We believe that there is fundamental significance in the discovery of new intercalation dynamics. In particular, we describe a completely new model to explain the early stages of graphite lithiation as a disorder driven non-equilibrium phase transition, where layers fill essentially independently with dynamics controlled by disorder which is clearly distinguished from the dense stages where strong coupling between the layers occurs and classic biphasic nucleation and growth of the new phase is seen. This model also resolves discrepancies and fundamental contradictions in the literature where, for example, the phase transitions between the different dilute phases are observed as apparent solid solutions.

It is well known in the field of non-equilibrium phase transition that hysteresis can arise purely from disorder(Vives & Planes, 1994a, p. 199). Hysteresis in the context of batteries has been understood in terms of nucleation overpotentials and path hysteresis in systems such as conversion reactions(Van Der Ven et al., 2022). However, there has been less discussion of the role of disorder and metastable pathway(Mercer et al., 2021) in intercalation materials. This is of significant importance to charge-discharge hysteresis in batteries and will be useful in understanding the phenomenon of zero-current hysteresis(Dreyer et al., 2010; Mercer et al., 2021), which has been largely understood as a multi-particle phenomena.

Furthermore, the dilute stages in graphite play a significant role in fast charging, where it is responsible for a higher proportion of total capacity at higher C rates (Heß & Novák, 2013). Finally, it will be interesting to explore whether this mechanism occurs in other layered materials that exhibit higher order staging in their intercalation compounds. For example, MX_2 phases such as Li_xNbSe_2 (D. C. Dahn & Haering, 1982) and Li_xTiS_2(J. R. Dahn & Haering, 1981; Van der Ven et al., 2008). Higher order staging is prevalent for larger ionic radii, hence the relevance may extend beyond Li-ion batteries. For example, graphite potassium intercalation compound is known to have staging up to stage 4 (Nixon & Parry, 1968; Onuma et al., 2021).

To highlight some of these significances, we have added the following to the *main text:*

Main text:

This led to the dilute stages being poorly characterised in comparison to the dense stages, despite their important role in fast charging[22]

Conclusion:

Such jumps are a generic feature of non-equilibrium phase transformations in systems with disorder30,31, and have profound implications for understanding the disorder induced zero-current hysteresis48,49 in batteries.

R1.7: *Figure 1 plots c d and e have the x axis as time in (min). This could be converted into the expected composition of LixC based on the constant current and mass of the electrode. A compositional dependence in the graphite would provide a better connection to what composition these avalanches are occurring, rather than the arbitrary time of the electrochemical experiment.*

This is a good suggestion, we have included a second x axis indicating the expected % LixC6 composition in the <u>*main text Figure 1*</u>:

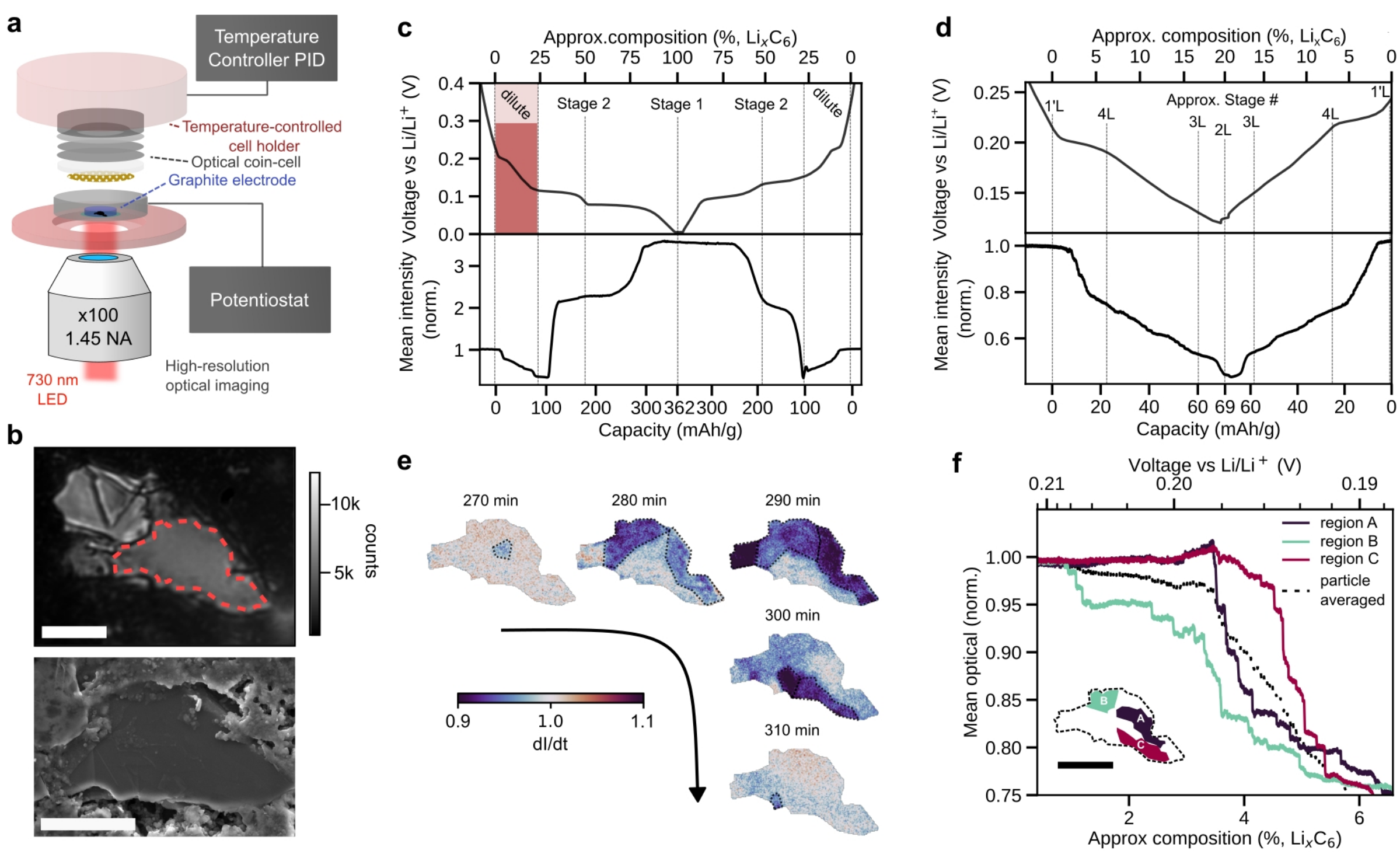

R1.8: *The experiments are conducted at C/20. Is the avalanche behavior rate dependent?*

This is an interesting question, and discussion of rate dependence has been added to the <u>*Supplementary Section 1.2.2*</u> (please see response to <u>*R4.1*</u>*)*. In short, we now have further support showing that, while intermittent step-like intensity curves can be observed at C/100 to 1C rates, but at higher rates (C/2, 1C) the avalanches tend to overlap and blur together, whereas they remain

clearly distinguishable at C/20 and C/100, which supports the expected behaviour (White & Dahmen, 2003).

R1.9: *There is no need to specify the unnormalized current (7 uA). Saying C/20 is sufficient or at least the mA/g or mA/cm2 to be more specific.*

This is a good suggestion and we have removed the discussion of current in the main manuscript.

R1.10: *In summary, this reviewer fully recognizes the novelty and value of the experimental results presented in this study. However, the proposed data interpretation and conclusions appear to be insufficiently documented and/or confronted with alternative mechanisms to ensure that the proposed methodology and discussion are sound and acceptable.*

We thank the reviewer for the interest and recognition of the novelty in the experimental results. We believe that the general concept of avalanches in disordered systems covers a broad category of experimental systems which vary widely in their structural origin of disorder. We have also better justified the model with further comparisons to the experiment. These discussions may be found in Supplementary Sections 1.2.1-1.2.3 and 2.3 (these can be found in responses to R3.5, R4.1, R4.2 and R4.6.3).

Finally, we have summarised some alternative mechanisms, model limitations and outlook on a future study in *Supplementary Section 2.4:*

2.4. Model limitations and alternative mechanisms

We have shown the results of RFIM as a minimal model that reproduces (1) staging and (2) avalanches under the simplest conditions. To do so, it incorporates uncorrelated disorder (random field) and coupling constants required to form staging compounds (Schon et al., 1988) in a discrete lattice. It is meant as a useful qualitative model reproducing many emergent experimental features and linking them to a well known theoretical framework, but not as a quantitative model. We apply periodic boundary conditions and assume that our experimental results are representative of bulk behaviour. We also assume that the dominant dynamics is that of quasi-static evolution via metastable minima in the lithium configurational energy landscape under slow driving. Thereby, we neglect diffusion and surface reaction by assuming that, on the simulated time scales, the reaction overpotential is small at slow C-rates (Supplementary Section 1.4) and that the solid state ionic diffusion is sufficiently fast ($x^2/2D$ ~ 0.1 s (Persson et al., 2010)).

This motivates future work on a more realistic, quantitative model with continuum, phase field formulations, similar to those previously applied to battery material systems (Chandesris et al., 2019; Fraggedakis et al., 2020; Smith et al., 2017) incorporating diffusion and surface reaction boundary conditions. Quenched disorder may also be realised as a random variation in coupling constants rather than site occupation energy, much like the discrete random bond Ising model(Vives & Planes, 1994a). In fact, although the disorder is spatially uncorrelated in the current model, the particle disorder has been partially experimentally characterised as arising from elastic deformation and kinks (see Section 1.1). Such correlated disorder can explain the emergence of persistent cluster regions, as seen in the experiment, and the spatio-temporal correlation between them, which our model is unable to reproduce. In order to reproduce intra-particle correlations discussed in the main text, it may be necessary to investigate coupled systems or to incorporate spatially correlated disorder to subdivide the simulation lattice into smaller regions.

We stress that (1) avalanches are not necessarily uniquely associated with specific regions of the graphite particle, their size and location are random and broadly-distributed (Supplementary Section 1.2.1), and (2) disordered features which can contribute to break-up of correlation and formation of metastable states span a wide range of length scales, whose impact on Li dynamics is difficult to quantify. Furthermore, lattice disorder may arise from the lithium insertion process itself, such as layer opening, shear stresses due to changes in layer stacking, and lattice expansion and stress redistribution. Indeed, a self-generated disorder with cycling has been previously reported in Martensitic transformations (F. J. Pérez-Reche et al., 2009; F.-J. Pérez-Reche et al., 2007) which is another prototypical system demonstrating avalanches and return point memory(Amengual et al., n.d.; Sethna et al., 1993, 2001). Together, these factors highlight both the importance and the challenges of developing more quantitative models incorporating realistic disorder.

<u>Reviewer #3:</u>

For all the technical mastery and inventiveness demonstrated in the manuscript, I believe it requires another round of careful review.

R3.1: *My main concern is that no avalanche behaviors have been demonstrated, contrary to the claims made in the title, abstract, and elsewhere in the manuscript. I appreciate the effort to introduce more physics and rigor into the field; however, when aiming for rigor, the work is held to a higher standard, and I do not believe the manuscript meets it.*

> We appreciate the referee's positive assessment of the results and new ideas that we present. In response to their criticism that avalanches have not been demonstrated, we have made significant improvements to the manuscript, including additional results, and further discussion of the relevant context, which is that of collective dynamics in slowly-driven disordered systems, and their modelling by random-field Ising models. All changes and additional data are highlighted in blue, some of which can be found in responses to other reviewers to prevent duplication.

R3.2: *The manuscript uses inappropriate terminology and analogies and does not convincingly support its claims. Not every abrupt transition should be labeled as an avalanche, and what is observed does not resemble avalanche behavior to me. The authors themselves provide damning evidence: the observed temperature dependencies are inconsistent with avalanching, and there are no signatures of amplification, which are present in related systems. It appears the authors may have been carried away by their analogies.*

> We strongly disagree with this comment. To address it, we have added substantial further justifications for our claims and analogies in the Supplementary Information and in this reply. We also updated the main text to avoid potential misunderstandings of our claims, including those mentioned by the referee. Finally, in the main text, we have removed the term "avalanche" until the terminology is defined later. We reply to their specific points in detail below.

R3.3: *I suggest the manuscript remove these analogies and focus on what is actually observed: stepping and interdomain correlations. The Glauber-like Ising model introduced does not prove or disprove any particular point. It seems more like a qualitative exercise, yielding the behaviors it was designed to produce. While these behaviors may be new to battery scientists and chemists, they are not novel to physicists or materials scientists.*

> We agree that our work on the RFIM uses ideas that are already established in physics and materials science. A key aspect of our manuscript is that we exploit this existing knowledge to understand the behaviour of Li filling of graphite. Our observation that a simple RFIM model can capture the observed stepping behaviour within the framework of graphite staging is new, because these steps have not been observed before. It provides a theoretical explanation for the hysteresis inherent in the jumps, and their broad distribution of sizes.

R3.4: *The nature of graphite lithiation is inherently complex, involving a succession of quasi–solid-solution and first-order transitions with phase coexistence. The manuscript's claim in the Introduction that the structural and dynamic aspects of this process remain poorly understood is, in fact, an understatement. As Timofeev-Resovsky once said, we have "progressed from false knowledge to true misunderstanding."*

Grey and co-authors aim to demonstrate rapid, "avalanche-like" phase transitions during the initial stages of Li intercalation into graphite, which are traditionally viewed as disordered solid solutions. However, I am not convinced that "avalanche" is the appropriate term. These are relatively fast transitions, observed with a modest 500 ms time resolution. In physics, the term "avalanche" typically implies a snowballing effect with amplification, such as in avalanche photodiodes (impact ionization). I see no evidence for such amplification in the data. In other contexts — avalanche upconversion in optics, Townsend discharges, superconducting flux avalanches — avalanches always involve multiplication or amplification of particles, excitations, or correlations.

The manuscript invokes Barkhausen noise, which is sometimes called a magnetic avalanche. This phenomenon is indeed a collective cascade of domain-wall motion: unpinning events release stress, destabilizing neighboring regions, producing a snowballing effect. Signatures of such amplification include crackling noise, with power-law distributions in amplitude, duration, and power spectrum. Similar principles apply to ferroelectric avalanches, historically called polarization Barkhausen noise.

> We thank the reviewer for appreciating the complexity of graphite lithiation. The picture that we find is very similar to that described by the referee: Li atoms flow into the graphite via collective cascades, which appear as steps/jumps in the filling. Such events are conventionally modelled by phase-field or Cahn-Hilliard type models, in which a single phase front moves smoothly across the entire system, as the material fills. In such models, the interparticle attraction within the layer (Schon et al., 1988) means that existing intercalated lithium will make adjacent sites more likely to be occupied, resulting in a positive feedback and a filling cascade under the right conditions. Our focus in this work is the role of disorder, which disrupts the (collective) phase front motion, leading to a sequence of finite jumps. Each jump is highly collective and involves the motion of many atoms, but it does not cover the whole system.
>
> Such behaviour, where a first-order phase transition is disrupted by disorder, can be captured by random-field Ising models (RFIMs), in which context the jumps are called avalanches. They can be defined as collective bursts in metastable driven systems, triggered by infinitesimal changes in external driving force which would be the magnetic field for Barkhausen noise, and the applied voltage in this present context(Baró et al., 2016; Sethna et al., 2001). Other disordered systems known to display such avalanches include helium condensation in aerogels (Aubry et al., 2014; Detcheverry et al., 2005) and Martensitic transformations (Planes & Vives, 2017; Vives & Planes, 1994a).

R3.5: *The authors have not demonstrated such characteristics typical of critical phenomena involving amplification. Without these demonstrations, they should not use terminology reserved for phenomena with proven amplification mechanisms. The observed phase transitions may or may not be avalanche-like, but this still requires rigorous proof.*

> One of the characteristic features of avalanches in the RFIM is a broad distribution of event sizes. In the athermal case, one finds a critical point where this distribution is a power law. For finite temperatures and generic values of the parameters (away from the critical point), the power law is cut off but a broad distribution of avalanches is retained (Sethna et al., 1993, p. 93; Vives & Planes, 1994a) , see also (Chern, 2017; Perković et al., 1995; Vives & Planes, 1994b; White & Dahmen, 2003; Yao & Jack, 2023).

To answer the referee, we have gathered further experimental evidence that supports this picture. To summarise:

(1) The size distribution of step-sizes is roughly power law distributed (see below).

(2) The spatial distribution of each step is complex – this is a subtle point and elaborated in the Supplementary (see below).

(3) The locality of each "region" is strongly correlated with its measured disorder (*Supplementary Section 1.1*, found in response to *R1.4)*.

(4) The temporal distribution of steps occurring in each region is strongly related to its disorder (*modified main manuscript,* round in response to *R4.6.3)*.

(5) The size distribution of steps occurring is each region is strongly related to its disorder (new *Extended Data Figure 3*, found in response to *R4.6.3)*.

(6) The size distribution of steps in different graphite samples are strongly related to its level of disorder (*Supplementary Section 1.2.3,* in response to *R4.2*).

(7) The driving rate dependence is highly consistent with previous reports of avalanching systems (*Supplementary Section 1.2.2,* response to *R4.1*).

(8) The intermittent steps retain similarities over different cycles, rather than occurring completely randomly *(main text Extended Data Figure 4).*

To further clarify what we mean by "avalanches", we have modified the main text as follows. In addition, points (1) and (2) has been addressed in a new *Supplementary Section 1.2.1* below.

Main:

... Empowered by this technique, we show that (de)lithiation of the dilute stages in graphite proceeds via a series of intermittent, step events broadly distributed in size. ... The events resemble crackling noise or *avalanches* observed during phase transitions in materials possessing static (quenched) disorder[27–30]...

The step-like intensity features of the individual regions revealed through optical imaging in the dilute stages are highly reminiscent of *crackling noise* or "avalanches" in disordered systems ... In these systems the avalanche dynamics are controlled by disorder and driving rate[36], and our C-rate dependence (Supplementary Section 1.2.2) and disorder dependence (Supplementary Section 1.2.3) is in agreement with the trends expected.

Supplementary 1.2.1: Signatures of avalanches

When a system with collective interactions, quenched disorder and close-to-athermal dynamics is driven slowly, it produces crackling noise or avalanches - intermittent bursts of activity due to jumps between locally stable configurations in a rough energy landscape (Baró et al., 2016; Sethna et al., 2001). When the system disorder is tuned (or self-organised) to near criticality, there will be a scale-invariant power law in avalanche size and length distribution (Perković et al., 1995). Beyond crackling noise, these system display hysteresis arising from disorder(Vives & Planes, 1994),

memory between cycles(Sethna et al., 1993) , and a complex spatial distribution of avalanches (Kuntz et al., 1999; Walsh et al., 1998). It is important to note that the intermittent steps still appear and are referred to as avalanches when the system is not perfectly athermal(Baró et al., 2016; Yao & Jack, 2023), not driven quasistatically (White & Dahmen, 2003), and with disorder not equal to critical disorder(Perković et al., 1995).

The transition during graphite intercalation in the dilute stages shares the following features with these systems. The step sizes are broadly distributed, following a rough power law (Fig. S3a). The step-size distributions are tuned to the intra-particle disorder (Section 1.1 and Extended Data Fig. 3) and sample disorder (Section 1.2.3). The intermittent steps are dependent on the driving rate (Section 1.2.2), and the individual steps do not occur randomly over different cycles, retaining similarities in their temporal distribution (Extended Data Fig. 4).

Finally, we stress that "region" labelling of the particle does not imply that the steps occur exclusively in the region. Shown in Fig. S4, we show the spatial extent of avalanches detected specifically in "region B". The spatial extent vary broadly in spatial structure, from one small cluster within the region to the entire particle, much like the avalanche clusters which appear in RFIM near criticality. This implies that, although the collective behaviour of lithium intercalation is strongly affected by the existing spatial structure of disorder (such as shown in Supplementary Section 1.1), the steps cannot be solely attributed to kink boundaries and steps occurring exclusively within one region confined by the kink boundaries. Indeed, avalanche-like steps have been observed without the appearance of persistent spatial regions, where each avalanche cluster was more randomly distributed spatially. For example in smaller particles (Supplementary Video S6) or on a relatively kink-free exfoliated graphite flake (such as that shown in Supplementary Fig. S7a).

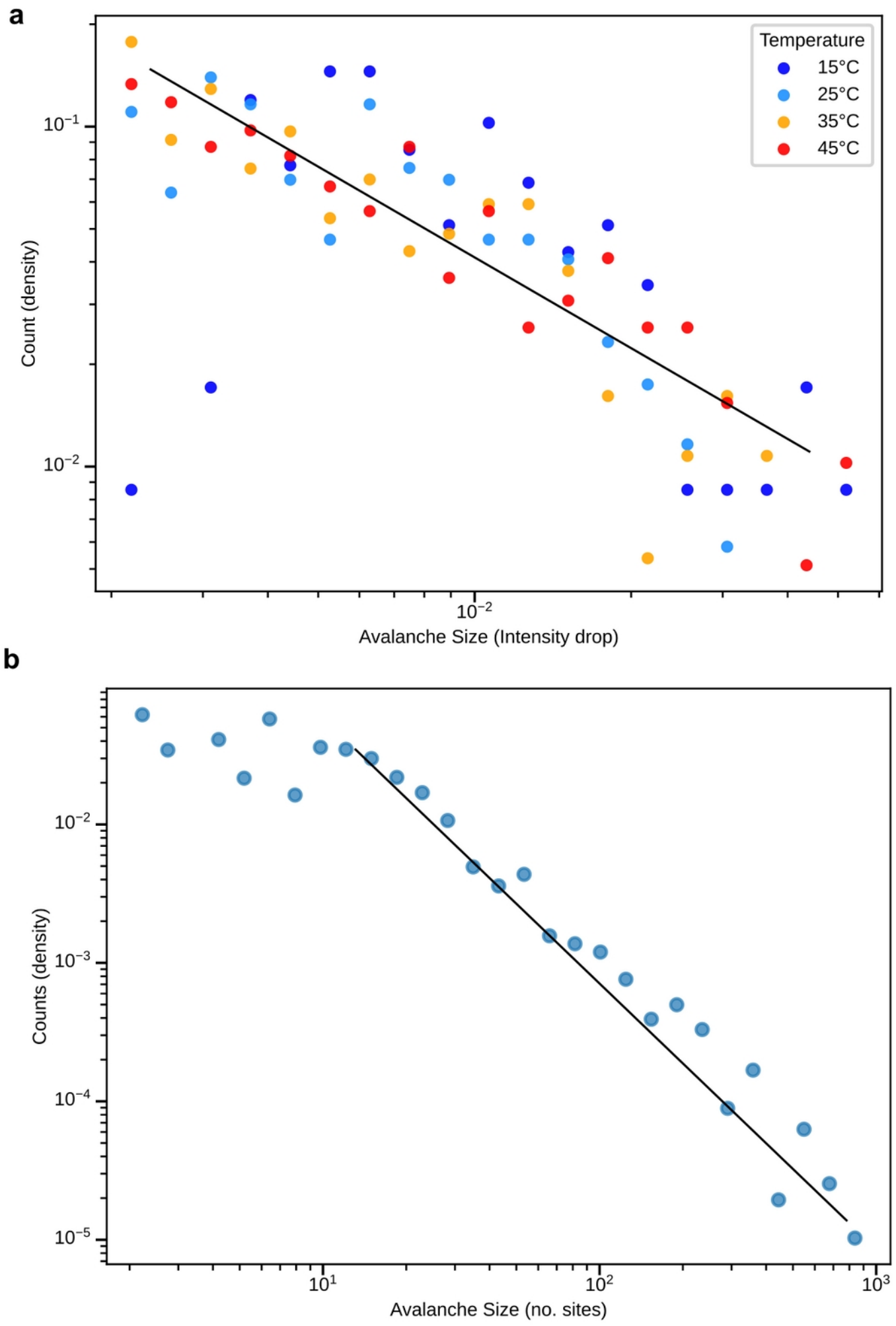

Figure S3 | Avalanche size distribution curves. A log-log histogram of avalanche size distribution is made for the experiment (**a**) and simulation (**b**). The experimental avalanche size is defined as the drop in intensity, with the intensity normalised to start of lithiation. The simulation avalanche size is defined as number of sites filled. Statistics are collected from region A, B and C during the 1'L - 4L transition during de/lithiation in cycle 1 and 2. The model statistics are collected during the 1'L - 4L transition from a single run in the 3D model (lattice size 128 x 128 x 64, beta = 10, h = 1, steps = 500,000), but similar power law statistics arise from the 2D model ($\tilde{h} \sim 0.4$) as well.

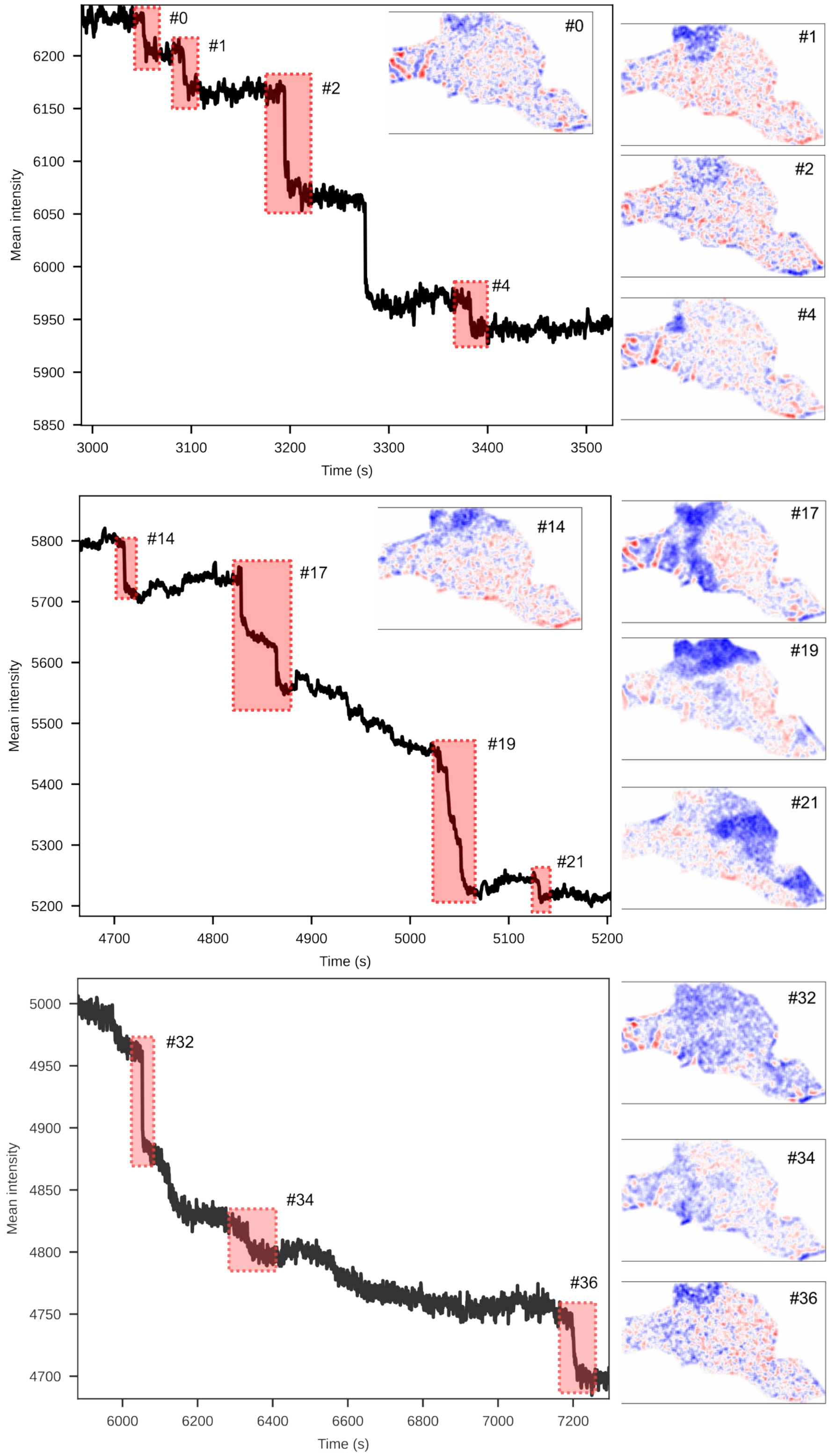

Figure S4 | Spatial distribution of each avalanche event. Each detected avalanche event is logged in the start and end time, and a differential image of the particle between these time points is constructed. A few of these differential images (**right**) is shown alongside the intensity trace (**left**), with the location of the avalanche events highlighted in the red boxes and labelled with numbers. The intensity trace and avalanche events are from Region B (from main text Fig. 1f) 15°C, cycle 1.

R3.6: *In particular, the random-field Ising model presented does not justify the use of avalanche terminology. It is not a proper dynamic model; it is a Glauber-like model for thermally activated metastable transitions. It imposes periodic boundary conditions, ignores Li diffusion, and lacks domain walls and defects. Its main purpose appears to be incorporating interlayer interactions. The statistics observed in the model (Figure S9) bear no resemblance to the experimental avalanche systems. By design, this type of model produces stepping once thermal excitation exceeds local barriers — which is intrinsic to the model and requires no computation. In this sense, the model is not instructive.*

This paragraph raises several points that we address in turn.

First, we insist that there is nothing "improper" about the RFIM as a dynamical model. It is consistent with the underlying physical principles which are (i) attractive intra-gallery interactions (ii) repulsive interactions between galleries (iii) disorder in the crystal structure (iv) an increasing potential that drives Li into the graphite (v) thermal activation over energy barriers.

It is true that some aspects of the experiment have been simplified in the model, in order to isolate the key principles at work. Periodic boundary conditions are used so that the model can mimic bulk behaviour even for moderate system sizes, this is standard in theoretical work. We ignore Li diffusion because this is fast, compared with the relevant time scales of filling. (The time scale for diffusion is of order $x^2/2D$ ~ 0.1 s (Persson et al., 2010) compared to ~10 hours per cycle). The very appearance of steps is indicative of this, where each step can last between ~ 1s to a few 10s, in accordance with diffusion, but at the time scale of one cycle (~40000 s), these are sharp steps appearing intermittently. RFIM without diffusion has been used to study the helium condensation in aerogels(Aubry et al., 2014; Detcheverry et al., 2005), where time scale of one cycle is also many hours.

The most severe assumption of the model is that the complicated structural disorder of the sample is replaced by a simple Gaussian random field, independent on each site. This is the conventional approach for the RFIM, it can be justified on grounds of universality, that the large-scale collective behaviour is independent of microscopic details of the disorder (Sethna et al., 2001) . In any case, an accurate atomistic model of the disorder in the graphite is beyond the scope of this work so some simplified model is needed. Its justification is that we find (a posteriori) that the resulting simple model does indeed capture the main features of the experiment.

We believe that the results from this simple model will motivate future studies with more quantitative models, which incorporate correlated disorder and diffusion. We have added a *Supplementary section 2.4*, where the model limitations are discussed (please refer to response to *R1.10*).

Regarding the ability of the model to reproduce experimental avalanches, we do not agree that our results "bear no resemblance" to the experimental measurement, we refer (for example) to *Extended Data Figures 3 and 4*, as well as the detailed comparisons in *Supplementary Section 2.3* and the *modified main manuscript* (both can be found in response to *R4.6.3*).We believe that the present model is the simplest possible demonstration of avalanches in a lattice with coupling resulting in staging compounds (Schon et al., 1988). Yet, in addition to avalanches, the model also reproduce many other qualitative features observed during lithiation of graphite. It is reproduces the sharp 1L-4L transition and continuous 4L-3L transition seen in the cell voltage-capacity plot and in the single

particle mean intensity plot. Past X-ray/Neutron diffraction studies have ascribed an apparently continuous (solid solution like) transition between Stage 4L to Stage 3L during which the (00n) peak position shifts continuously(J. R. Dahn, 1991, p. 19; Didier et al., 2020). The model behaviour reproduces this, where a mixture of intergallery distances evolves via intermittent filling of clusters (in the xy plane) via a non-equilibrium pathway, from being Stage 4 like filling (~0.25) to Stage 3 like filling (~0.33). Such a transition will produce a continuous peak shift in the lattice Fourier transform which the 3D RFIM showcases more clearly.

R3.7: *It is especially concerning that, as the authors themselves note on p. 12, the observed temperature behavior is inconsistent with expectations from Glauber-type models. Explaining this discrepancy as a deficiency in the detection algorithm is not sufficient. A model alone cannot establish similarity to avalanches known in other systems involving defect pinning at domain walls; it remains purely speculative.*

We agree with the reviewer, and in response to this comment – and those of other referees – we have refined our discussion of this temperature dependence. This is provided in detailed in response to R4.5, where we detail the *removal of old Fig. 4 and discussion in the main text*, and a more nuanced discussion of temperature trends in Supplementary Section 2.3 (found in response to R4.6.3).

In short, the old simulation results were over a much wider temperature range than that of the experiment. We repeated the model over a smaller range and found that the shape of the distribution does not change, but the thermally induced back hopping ("reverse") events occur much more frequently – this is consistent with the experimental results. Note that we study the model at an intermediate regime, where thermally activated jumps play a role, but overall the model remains under athermal, non-equilibrium dynamics at the driving rates studied. This has been described in previous studies, being particularly relevant in Martensitic transformations which display avalanches with thermal cycling(Baró et al., 2016; Vives & Planes, 1994b).

Regarding the referee's claim that a model alone cannot establish similarities to avalanches in (for example) magnetic systems with Barkhausen noise: we repeat that this is not our claim. We argue that the RFIM captures the main aspects of the experiment because such features – including avalanches – are generic in systems with (i) proximity to first-order phase transitions (ii) disorder (iii) slow driving via an external field(Baró et al., 2016; Sethna et al., 2001). We hope that our extensive revisions and discussions are sufficient to convince the referee on this point.

R3.8: *A more compelling part of the manuscript is the spatio-temporal correlation between domains, analyzed using a graph representation of domain boundaries. The data convincingly show correlations between stepping in adjacent domains. However, these correlations do not prove an avalanche mechanism or validate the model. They suggest varying degrees of interdomain connectivity without clarifying its origin. This is disappointing, as extensive effort in establishing correlations yields limited insight.*

We are glad that the referee shares our interest in the spatio-temporal correlation. It is true that the current model is unable to reproduce the intraparticle inter-region correlation. We hope to pursue a more extensive study in the future on this (please refer to R4.7). On the other hand, we now have a better understanding on many other aspects of correlation.

For one, we have pursued further ex-situ characterisation of disorder on the graphite particle, and have a better understanding on the origin of the clustered regions (main text Fig. 5a), and on the spatio-temporal correlation between them (main text Fig. 5b). We discuss this in Supplementary

Section 1.1 (refer to response to *R1.4*). The elastic disorder due to kinks and bends have been characterised and are in excellent agreement with appearance of regions and their connectivity.

Reviewer #4 (Remarks to the Author):

The work by Han et al. studies the emergent dynamics of ion insertion and extraction in graphite particles, one of the most relevant anode materials used in Li-ion batteries. The authors use operando optical scattering microscopy coupled with classical electrochemical measurements to unravel the behavior of graphite under ion (de)intercalation for filling fractions up to 50%. Their objective is to reveal the emergent phase transitions rooted in the ion--ion interactions that coexist within and between neighboring layers of graphite in the dilute regime, a region that remains poorly understood even today. Their optical signal measurements reveal the existence of an 'avalanche'-like behavior in the dilute regime (0.25 to ~0.10 V vs Li/Li^{+}) when graphite is cycled under a C/20 rate and different temperatures (15 oC, 25 oC, 35 oC, and 45 oC). They attribute this behavior to the inherent quenched disorder present within the graphite structure. To test this hypothesis, they performed numerical Monte Carlo calculations based on a model Hamiltonian that describes nearest and next-nearest neighbor interactions between spins (occupied and unoccupied sites), which demonstrated very similar phenomenology to what the experiments showed. Lastly, the authors close their article with a correlation map across the single graphite particle they studied, demonstrating how specific regions undergo correlated ion insertion and extraction.

Overall, this work is interesting as it introduces a perspective grounded in fundamental physical chemistry and physics to one of the most classical materials used in Li-ion batteries. It also advances the understanding of graphite as an intercalation material in a regime that is rarely explored with such characterization methods, largely due to the inherent difficulty of performing in-operando measurements. I personally enjoyed the ideas behind this work, though one could argue that the observations and phenomenology presented here are limited to a single material. In addition to these comments, I have more specific questions and suggestions for the authors regarding their work. These are:

***R4.1:** I find it necessary to pursue experiments at both much lower and higher C-rates, ideally below the diffusion-limited current in these graphite systems. This would clarify how equilibrium and non-equilibrium factors influence the observed behavior and whether non-equilibrium forcing smooths out the discrete jumps seen in the optical measurements.*

We agree with the reviewer and now have further results from repeating the experiment at 2C, 1C, C/2, C/20 and C/100. The intermittent, step-like trend is seen at all rates, including at faster C-rates: at slower rates, there is a clear sequence of well separated avalanches where as at higher rates, there is a tendency for avalanches to overlap, leading to blurring and a continuous sequence of peaks in the differential intensity. This is exactly as predicted by previous works on Barkhausen avalanches and related systems. We have included a sentence in the manuscript and added a discussion section to *Supplementary Section 1.2.2:*

Main:

... other graphite samples with different levels of disorder (Supplementary Section 1.2.3), where more disordered graphites such as MCMB1028 displays smaller step sizes and vice versa. There is also a driving rate (C-rate) dependence, with faster driving resulting in overlapping of avalanches which eventually become difficult to distinguish[38,47] (Supplementary Section 1.2.2).

Supplementary 1.2.2: Avalanche dynamics at different C-rates

An electrode with the same composition as studied in the main text of the paper was cycled at different C-rates at 23 °C, following a C/20 formation cycle. The mean intensity from a small single particle, and corresponding differential intensity is shown in Figure S5, for C/100, C/20, C/2 and 1C. The intermittent intensity steps were most clearly observed for slower rates (C/100, C/20), with a clear, well separated sequence of steps of various sizes throughout de/lithiation. This is better illustrated in the lower panel, showing the sequence of sharp pulses in the differential intensity plot. At higher rates, the steps became blurred due to the overlap of avalanches resulting in a longer, continuous sequence. This is clearly seen in Figure S6, where the experiments at C/100 and 1C are compared. The total number of frames per half-cycle is kept similar (~100k frames), such that we are not limited by our temporal resolution, and a similar region (1L-4L transition, between 1.0 to 0.8 mean intensity) is shown. The well separated, sharp steps at C/100 results in correspondingly well separated and large peaks in the differential. At 1C, there is a tendency for a longer, continuous-like drop in intensity. This is consistent with findings from other avalanching systems, where at higher driving rate, the avalanches merge and combine, resulting in larger and longer pulses (Durin & Zapperi, 2004; White & Dahmen, 2003).

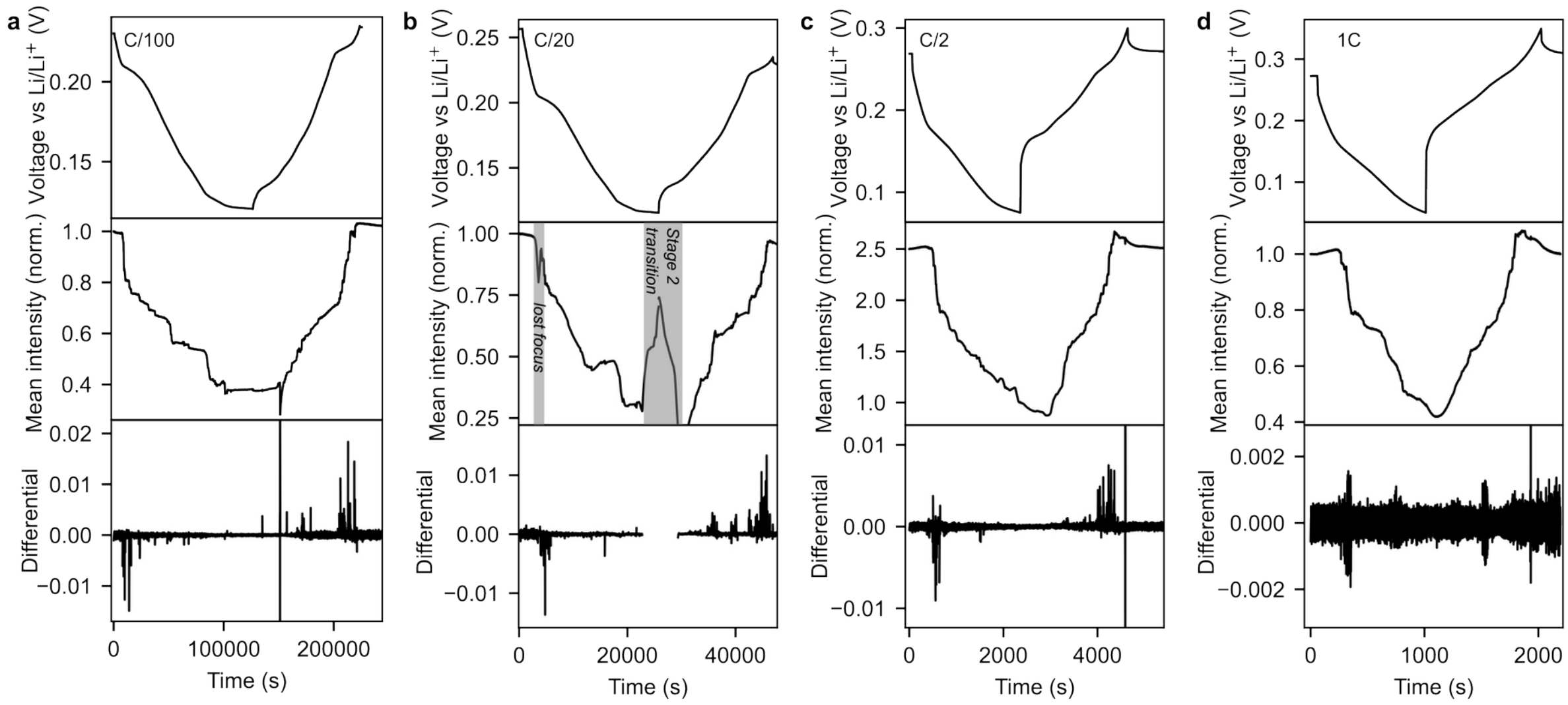

Figure S5 | Effect of C-rate. An optical coin cell, with lithium metal counter and same graphite electrode composition as studied in the main text (KS44:PVDF:super-p = 50:25:25, 80 µm self standing) is cycled with imaging at C/100 with 2 fps acquisition **(a)**, C/20 with 2 fps acquisition **(b)**, C/2 at 20 fps acquisition **(c)** and 1C at 100 fps acquisition **(d)**. The upper panels show the corresponding cell voltage during cycling. The middle panels show the corresponding mean intensity extracted from a small single particle during cycling, normalised to start of lithiation. The bottom panel show the raw mean intensity differential signal during cycling, calculated after applying a median filter (kernel = 15) to the intensity data.

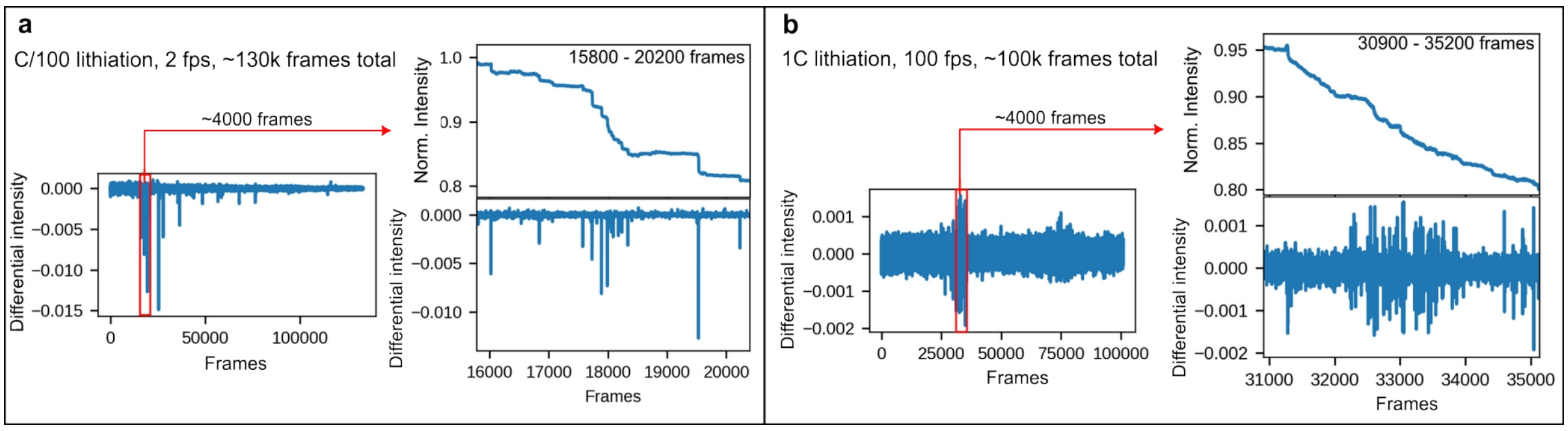

Figure S6 | Detailed comparison between C/100 and 1C. The single particle intensity data is compared between C/100 **(a)** and 1C **(b)** rates. The image is acquired much faster for 1C rate, and the total number of frames per half-cycle is kept approximately the same for both rates (~100k frames). A close up of ~4000 frames are selected during the 1L-4L transition, and the raw normalised intensity and the differential intensity are shown.

R4.2: *The authors appear to have focused their analysis and conclusions on measurements from a single particle. I assume that these observations were reproduced in multiple samples, and it would therefore be beneficial to demonstrate consistency across several particles. While I understand this may require significant effort, such validation would strengthen the claim that the observed behavior is intrinsic to graphite and not specific to one experimental sample.*

We thank the reviewer for raising this concern. We have run several different graphite samples, including disordered, rounded graphite in electrode (MCMB1028), flake-type battery graphites in electrode (Timcal KS44, Hitachi MAGE3), and highly crystalline samples exfoliated on glass (Kish graphite). Avalanches can be observed in all samples, however they are most clearly seen in more crystalline, flake graphites (KS44, MAGE3, and Kish graphite). For more disordered, polycrystalline graphites with nano-sized grains (MCMB1028), we see smaller avalanches, with more continuous like behaviour as it becomes difficult to resolve individual avalanches clearly. We stress that this is consistent with the theory which predicts smaller and smaller avalanches with increasing disorder, and consistent with resolution of our optical set up (~200 nm) and the resultant coarse graining. We have added this to the *Supplementary Section 1.2.3*.

Supplementary 1.2.3: Disorder and sample dependence

To understand the effect of graphite-type on the step-like intercalation behaviour, we have repeated the experiment with different graphite samples. We compare MCMB1028 graphite, Hitachi MAGE3 commercial battery graphite and exfoliated Kish graphite, in order from most to least disordered. Both MCMB1028 and MAGE3 were characterised in electrode, whereas the Kish graphite was first exfoliated on glass, with a (KS44 flake) graphite electrode (33:33:33 = KS44 graphite : porous carbon : PVDF) cast on top in order to observe a constant-current behaviour with a consistent rate. All samples were run at C/20, 23 °C after a formation cycle (C/20) in an optical coin cell (Figure S7). Typical SEM images from corresponding samples can be found in Figure S8.

All samples showed some intermittent, step-like behaviour, which is particularly clear in the differential intensity plots (Fig. S7d-f lower panels), however, the steps were much smaller and harder to resolve in the disordered, MCMB1028 sample (see zoomed inset, Fig. S7f middle panel), resulting in the smallest peaks in the differential plots. In the least disordered Kish graphite, much more clearly separated, sharp steps are seen, resulting in larger peaks in the differential. This is

highly consistent with theoretical avalanche dynamics (Perković et al., 1999; Sethna et al., 1993), where disorder beyond the critical disorder results in larger avalanches being cut-off, and more smaller avalanches overall.

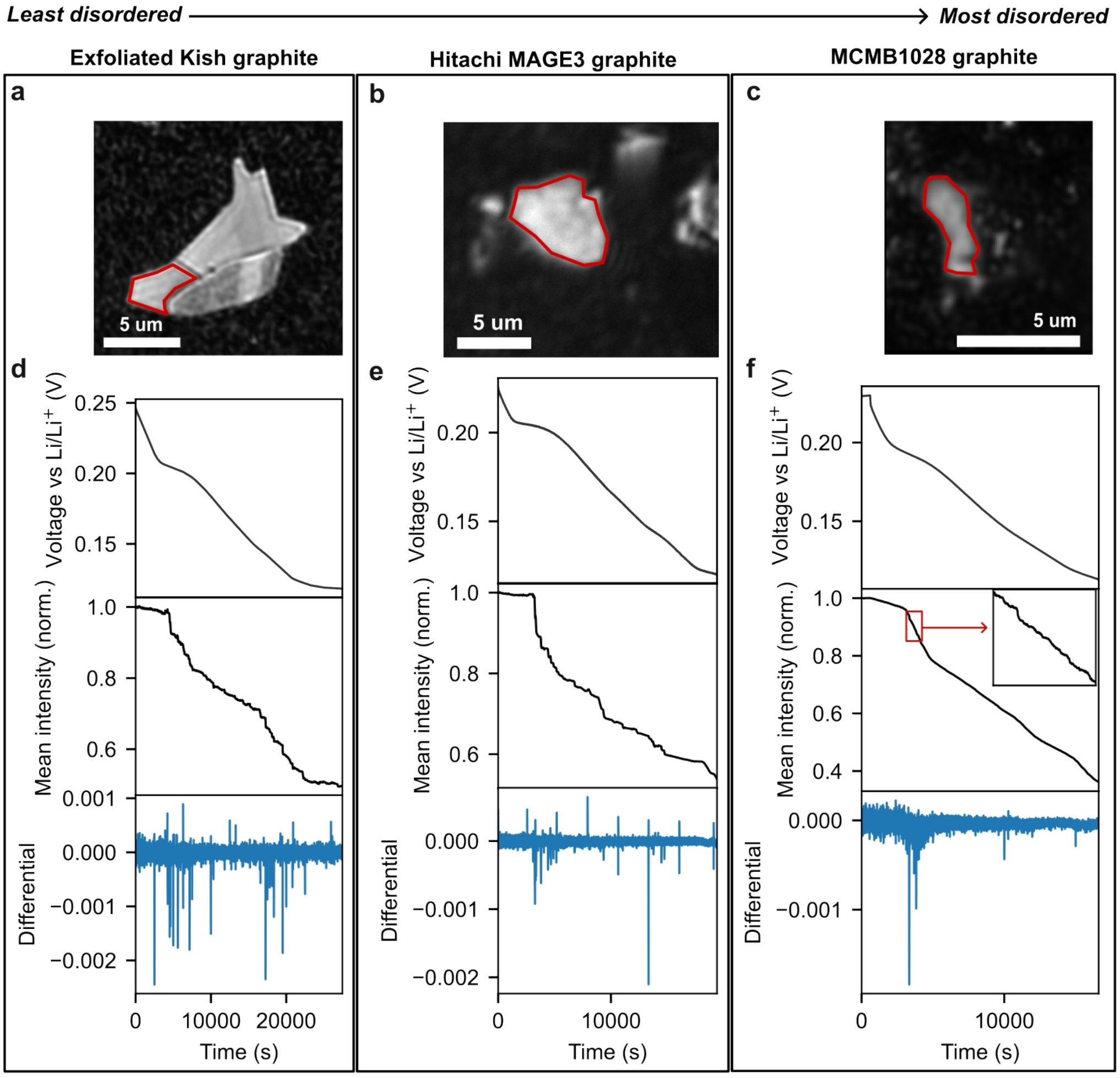

Figure S7 | Step intensity behaviour in other graphite types. a, Optical image of single particle graphite embedded in electrode (Hitachi MAGE3, LP57/FC2 electrolyte using 40:40:20 = AM:porous carbon:PVDF electrode and lithium metal counter electrode). Region indicated by red line was used for mean intensity calculations. **b,** Optical image of single particle graphite embedded in electrode (MCMB1028, LP57/FC2 electrolyte using 40:40:20 = AM:porous carbon:PVDF electrode and lithium metal counter electrode). **c,** Optical image of Kish graphite exfoliated onto glass, electrically connected with a porous electrode slurry (33:33:33 = KS44 graphite:porous carbon:PVDF). The bulk region of the exfoliated particle (indicated in red) was used for the mean intensity calculations. **d,e,f,** Cell voltage vs Lithium metal counter electrode (top panel), mean intensity (middle panel), and differential intensity (bottom panel) plotted against time. Mean intensity has been normalised to the start of cycle. Differential intensity is calculated after applying a median filter (kernel = 15) to the mean intensity. Red square ((f), middle panel) illustrates the location of the zoomed inset, which highlights the step-like features in the MCMB1028 graphite intensity plot.

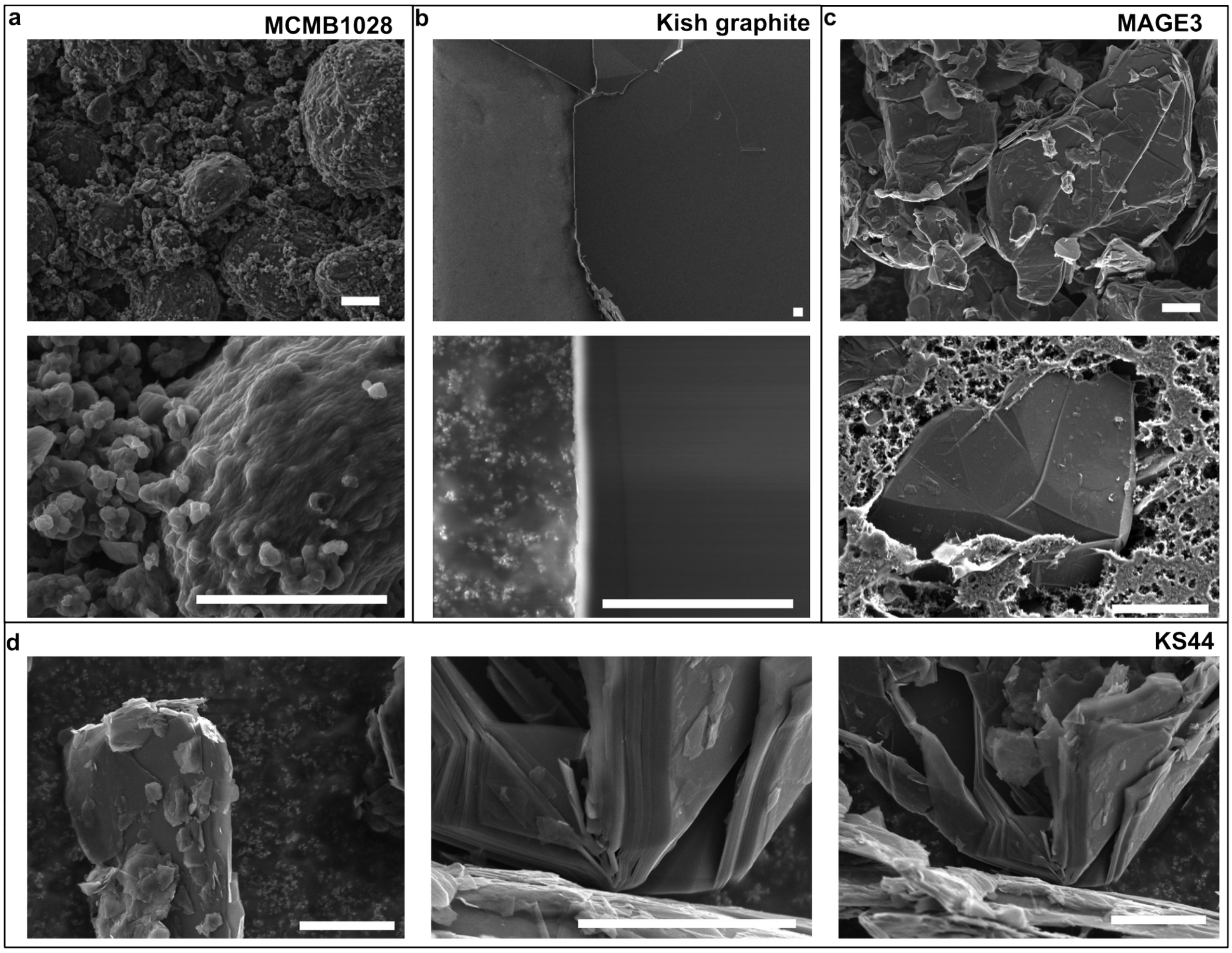

Figure S8 | SEM images of different graphite samples, highlighting kinks and bends. SEM images showcasing typical sample morphology of MCMB1028 **(a)**, Kish graphite **(b)**, Hitachi MAGE3 **(c)** and Timcal Timrex KS44 **(d)**. All images measured on the original powder samples, except for (**c**) lower panel, which shows a typical graphite flake embedded in an electrode. All scale bars, 2 um.

R4.3: *In the experiments, the filling fractions start and end at the same point, indicating that the endpoint for lithiation matches the starting point for delithiation. However, in the simulations, this is not the case. Why? I can imagine this might arise if each simulation begins from a newly sampled initial configuration. If so, the simulations do not fully mirror the experimental protocol, which may explain the observed hysteresis.*

This is a valid concern and a good observation, the relevant figure is Fig. S19 in the new Supplementary. To explain, we have attached a figure below for the reviewer's reference. We do not reset the initial configuration on the reverse run because, as the reviewer mentions, this will have a significant effect on the outcome of the hysteresis curve. Notably, the simulation becomes more asymmetrical and the hysteresis decreases when the reverse initial configuration is reset to fully filled. However, the reason why the starting point and end point do not match is because we slightly overfill during the simulation, beyond x = 0.5, and only plot the relevant potentials.

In order account for this, we have compared the original simulation to one that is stopped earlier, at a lower potential, where we only have the configurations of interest (compare black vs red curve in (c)). It show similar behaviour, noting that some differences are expected between runs. However, to

further account for this, the new results of the 3D RFIM model (see *Supplementary 2.2* in response to *R4.6.1* below) were simulated this way, without overfilling, such that the start and end points, when plotted, match.

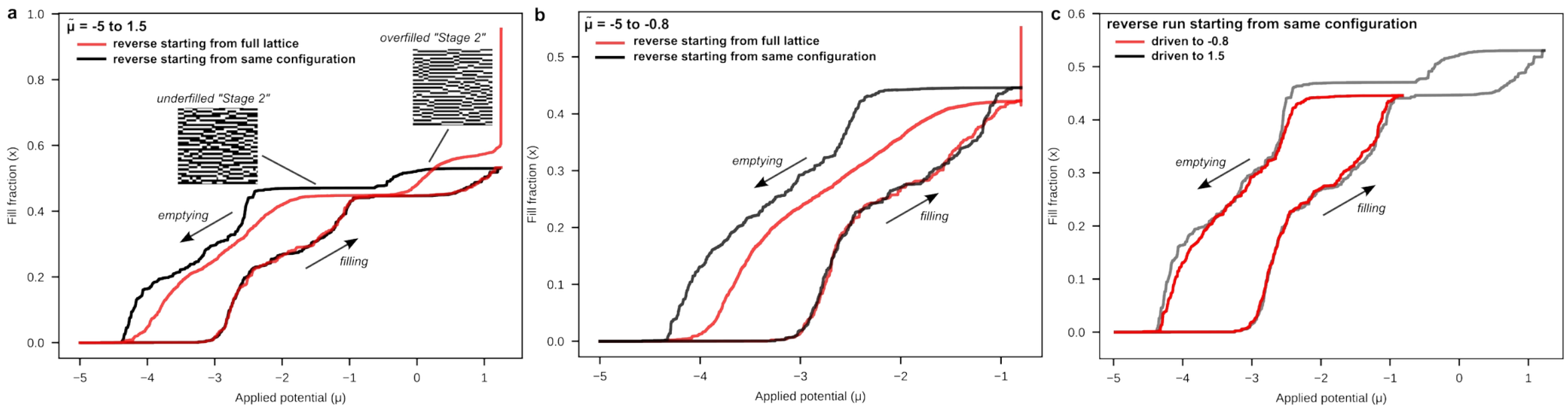

R4.4: *On a related note, there is a clear voltage jump between the end of lithiation and the start of delithiation, indicating a reaction overpotential, especially at lower temperatures. It would therefore be valuable to show that at lower applied C-rates, this overpotential decreases and the material approaches quasi-static lithiation and delithiation. This would effectively decouple thermodynamic from kinetic effects, which I believe aligns with the authors's broader message.*

We acknowledge that overpotential is an important and complex discussion point. As the reviewer mentions, the hysteresis at the particle level can be understood as a combination of kinetic factors, including the reaction overpotential and mass transport, as well as a hysteresis arising due to the particle following an inherently metastable, non-equilibrium phase transition pathway. The latter is an inherent hysteresis arising due to metastable and non-equilibrium nature of the disordered phase transition.

The effect of mass-transport related overpotential is likely trivial in our experiments at 25°C and above, and this is demonstrated below in the mean intensity vs capacity plot. The mean intensity from the particle is plotted against the cell capacity, showing minimal lag between de/lithiation curves at 25°C and above, which indicates the particle is not lagging behind the rest of the electrode. However at 15°C there is a lag in the mean intensity, likely due to mass transport limitations.

However, we cannot eliminate the role of reaction overpotential from the observed intensity plots. We have taken further results at slower and faster rates, as the reviewer suggested, to show that we can further reduce the overpotential. Nonetheless, we appear to have been quite close to the quasi-static limit at C/20 rate, with the 1L-4L hysteresis reducing from ~16 mV (C/20, 45°C from main text experiment), to ~10 mV (C/100, 45°C). A zero-current hysteresis of ~10 mV has been reported previously, in which it was also attributed to metastability of de/lithiation pathways (Mercer et al., 2021).

We have added a new *Supplementary Section 1.4* highlighting the role of hysteresis. Furthermore, we have restructured the discussion and Fig. 4 in the manuscript, removing some of the discussion on temperature trends. We have elaborated this further in the next response (*R4.5).*

Supplementary 1.4: Additional Electrochemistry

To better understand the electrochemistry under very slow C-rates, a normal coin cell with a graphite electrode of the same composition as that studied in the main text was cycled at C/100, C/20 and C/2 in a potential range approximately corresponding to the dilute stages. A dQ/dV plot calculated from this is shown in Fig. S10, where the C/100 rate shows a reduced 1L-4L hysteresis compared to C/20, which further reduces with increasing temperature. The hysteresis values for the 1L-4L calculated from the cell voltage and single particle measurements (*) can be found in Table S2. The dQ/dV at C/20 and C/100 shows very small peaks (denoted 2a and 2b in Fig. 2 in the main text), which is highlighted in Figure S11. An interpretation of this is discussed in Supplementary Section 2.3.

Overall, the voltage hysteresis observed in the cell and in the single particle intensity can be divided into (1) mass transport and particle-electrode connectivity limitations, (2) reaction overpotential and (3) an intrinsic zero-current hysteresis arising due to non-equilibrium, athermal phase transition. In Fig. S12, the single particle intensity (averaged over entire particle), is plotted against cell capacity. At 25°C and higher, it shows very good overlap during lithiation and delithiation, demonstrating that the particle does not lag behind the electrode and little mass transport overpotential exists, except at 15°C.

The reaction overpotential and intrinsic zero-current hysteresis cannot be separated, however, we have tabulated the approximate 1L-4L voltage hysteresis from the cell potential at different rates, and show that at C/100 at 45°C there exists ~9.8 mV of voltage hysteresis. This is the same as the zero-current voltage hysteresis reported previously(Mercer et al., 2021), and from this it is reasonable to assume that the voltage hysteresis in single particle intensity measurements presented in the main text is mostly composed of a combination of reaction overpotential and intrinsic hysteresis.

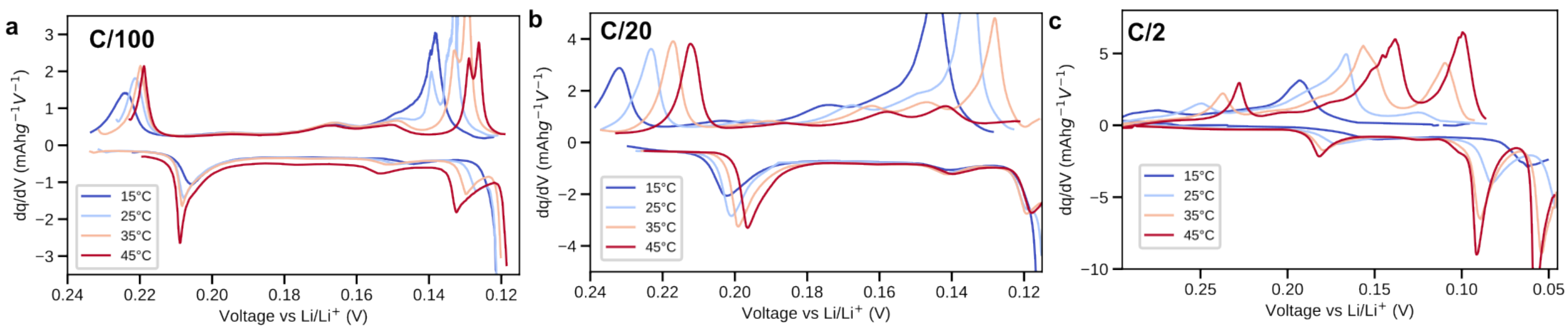

Figure S10 | C-rate and Temperature dependent dQ/dV. The differential capacity (dQ/dV) plots are calculated from a constant current charging at **a,** C/100, **b,** C/20, **c,** C/2. The voltage range is limited between ~250 mV and ~115 mV to only cycle in the dilute stages for C/100 and C/20. Note that in C/2 we lithiate beyond the dilute stages at 25°C and higher, where the two dQ/dV peaks at the lowest voltage corresponds to the two dense stage transitions. A normal coin cell with lithium metal counter and a graphite electrode of the same composition as that studied in the main text (KS44:PVDF:super-p = 50:25:25, 80 µm self standing) is used, with a custom built heated cell holder.

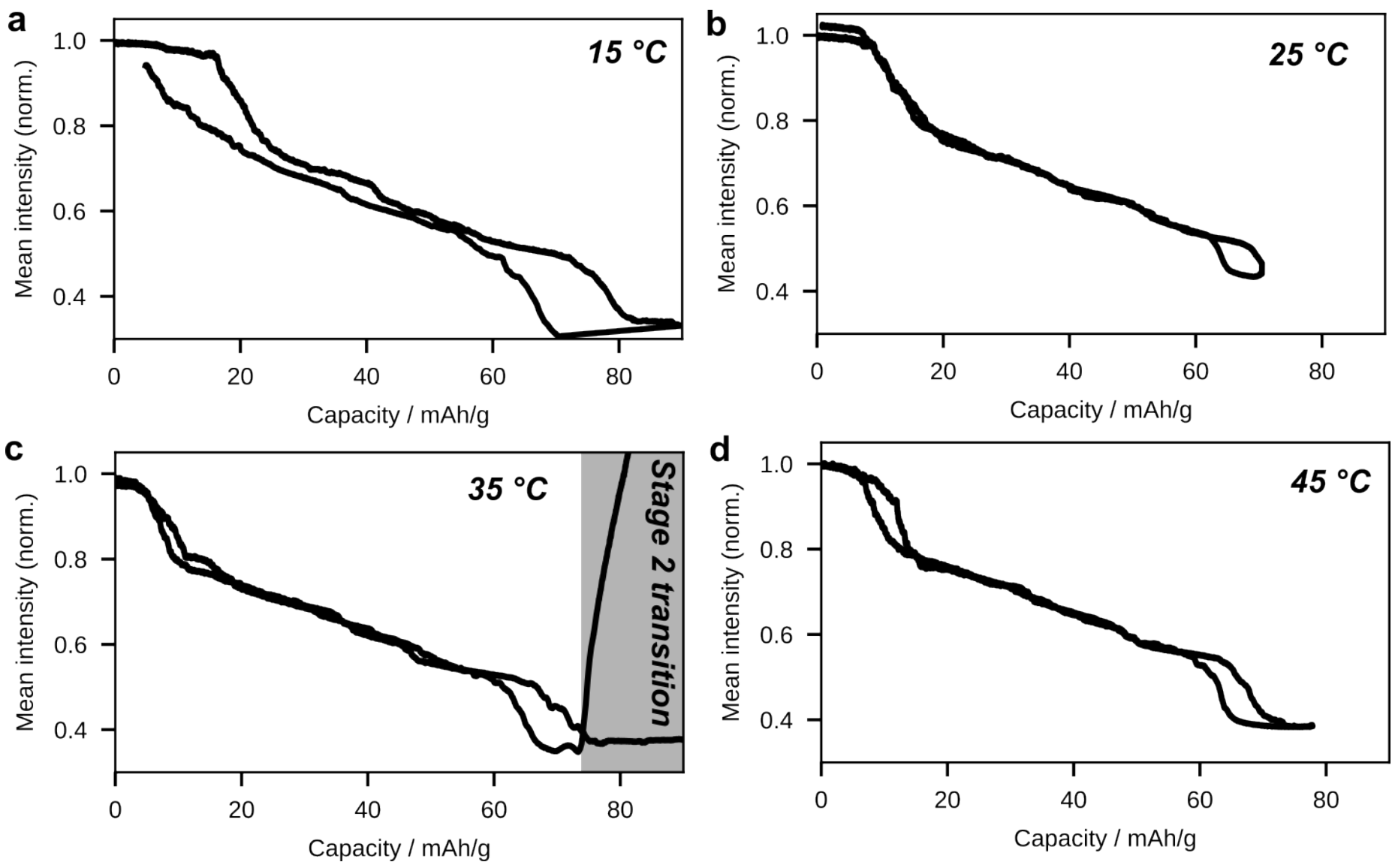

Figure S12 | Particle mean intensity vs capacity. Average particle intensity is plotted against cell capacity, from same dataset shown in Figure 2a of the main text. Data from cycle 1, at 15 °C, 25 °C, 35 °C, 45 °C, in (**a**) – (**d**) respectively.

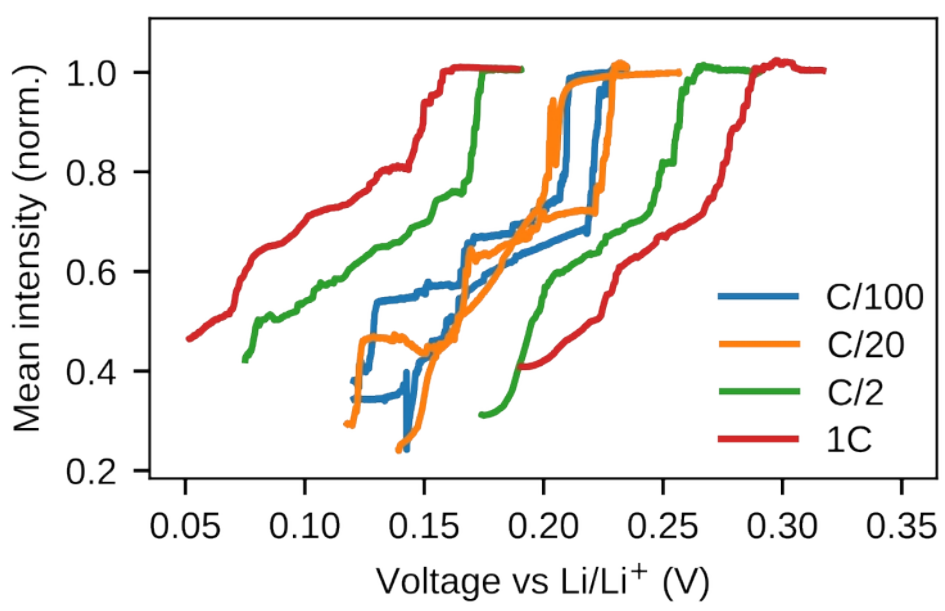

Figure S13 | Mean intensity vs Cell voltage at different rates. The mean intensity from a small single particle is shown against interpolated cell voltage. Same dataset as shown in Figure S5-6.

	**C/2**	**C/20**	**C/100**
**15°C**	120 mV	30 mV (*41 mV**)	18 mV
**25°C**	79 mV (*84 mV**)	23 mV (*25 mV**)	13.2 mV (*14 mV**)
**35°C**	58 mV	18 mV (*20 mV**)	11 mV
**45°C**	46 mV	16 mV (*18 mV**)	9.8 mV

Table S2 | 1L-4L voltage hysteresis measured from cell potential during constant current de/lithiation at various C-rates (Fig. S10). ***single particle 1L-4L voltage hysteresis, measured from mean intensity over particle plotted vs cell voltage.***

R4.5: *The argument: 'However note that the step-detection algorithm is prone to detecting multiple step events as a single large event as the time separation between steps decreases. Therefore, at higher temperature as the events bunch more in time (see Fig. 2b), clusters of smaller events may frequently be detected as a single large avalanche, resulting in deviation from the trend,' appears rather weak in explaining the disconnect between the phenomenological simulation model and the experimental results. I would suggest a more comprehensive investigation into why the model fails to capture the experimental trends.*

We agree with the reviewer, and have reconsidered our analysis. In the experiment, our temperature range (T = 288 K to 318 K, approx. 10% increase) is far smaller than that presented in the model (scaled $\tilde{T}$ = 0.042 to 0.125, approx 300% increase). We are effectively operating very close to the upper limit of the model temperature range (close to $\tilde{T}$ = 0.1), close to the critical region where the behaviour begins to be thermal fluctuation dominated. Correspondingly, the previous model experiment comparison was not valid.

We have simulated a smaller region (~30% increase around $\tilde{T}$ = 0.1) in the model, and both the hysteresis and distribution of avalanches sizes display negligible change, with its shape prone to outliers, which is much like the experiment. However, like the experiment, there is a notable increase in the number of thermally-induced jumps in the opposite direction of driving (termed "reverse avalanches" in the paper). We believe that this is of notable significance.

We have restructured the discussion on temperature dependence in the manuscript, by removing *Fig. 4* and significantly shortening the discussion on temperature trend of hysteresis and avalanche size distributions. Instead, we have changed the discussion to a more qualitative comparison to the model, with a focus on disorder and driving rate, and moved the discussion of temperature to the *Supplementary Section 2.3* (please refer to *R4.6.3*).

R4.6.0: *The current modelling approach appears very simple. While I appreciate the value of simplicity in a complex system such as ion intercalation materials, the present model is quite far from representing the structure of graphite.*

We thank the reviewer for appreciating the value of our model and for the helpful suggestions. We provide an extensive response below. To summarise, we have explored a 3D RFIM and made major revisions to the way we discuss and compare the model to the experiment. We believe that the current RFIM approach holds a lot of promise and have made an effort to highlight the complex emergent features of the current model, which match the experiment very well. These have been highlighted in the modified main text, and in Supplementary Section 2.3.

R4.6.1: *Specifically, the model is purely two-dimensional, with quenched disorder only in the yz-directions, completely neglecting the emergence of nucleated phases in the xy-plane, where surface tension effects are expected to play an important role.*

We agree that it will be valuable to extend the model to 3D to observe the effect of nucleated phases in the xy-plane. We have explored the 3D model and note that the model qualitatively retains the same features. We have added a sentence in the *main* manuscript to highlight this, and further elaborate the results in *Supplementary section 2.2*. We have also added *Supplementary Videos S5, 6 and 9,* to further highlight the similarity to the experiment:

Main:

The equivalent 3D model behaves similarly qualitatively (see Supplementary Section 2.2) and we show the 2D model here for simplicity.

2.2 3D RFIM:

In order to demonstrate that we retain the same qualitative behaviour in the 3D model, we have simulated a 3D lattice with the attractive intralayer coupling extended into the y-direction. Typical parameters used can be found in Table S4. Exploration of temperature and disorder parameter space revealed that the transition is qualitatively similar, as shown in Fig. S16. The transition is sharp with a large hysteresis at low temperature and disorder, and becomes highly rounded, with hysteresis disappearing at high temperature and disorder. In between the two regimes, at intermediate temperatures ($\tilde{T} < 0.2$) and disorder ($h \sim 1$), we observe a sharp-gradual-sharp trend during filling and emptying. Due to the larger size of the lattice, more simulation steps were necessary to achieve close to quasi-static driving rates. The effect of model driving rate is shown in Figure S17.

An example is shown in Fig. S18a, where the lattice filling dynamic is shown during a filling simulation. We take slices from the lattice at 3 time points (marked i – iii) and show an XY slice (Fig. S18b) and an XZ slice (Fig. S18c). The XZ slice is qualitatively similar to the 2D model, where due to disorder and frustrated interactions, there is a mixture of distances between filled galleries during the simulation and at no point does any pure staging periodicity dominate. A Fourier transform of the lattice, computed by taking the 3D Fourier transform and averaging kx and ky directions, is shown in Figure S18a lower panel. A broad peak, equivalent to the (001) peak in diffraction, appears at approximately $k = 0.25$, and continuously shifts to $k \sim 0.33$ in the continuous, sloping region between the Stage 4M and 3M as marked in the upper panel.

In the XY slice (Fig. S18b), we see an emergence of a cluster with a complex spatial distribution. Although in the example shown, there is a single connected cluster, in other layers and simulations, there may be multiple disconnected clusters, which eventually become connected via a series of avalanches. The evolution in a single XY slice, resembles a 2D standard RFIM model, where filling progresses via intermittent clusters due to disorder and athermal dynamics, with the added complication of repulsive interlayer interactions. This can be seen in the filling curve of individual XY layers, shown in Fig. S18d. Interestingly – although there is no dynamic “layer shuffling” or thermal rearrangements due to quenched disorder and athermal dynamics – still some layers may empty while others fill due to the repulsive interlayer interactions. A combined animation of the simulation can be found in Supplementary Video S9.

J	*K1*	*K2*	*K3*	μ range	*Total mcs steps*	*Lattice size*
1	3	0.75	0.333	-10, -2	100,000	64 (x), 64 (y), 32 (z)

Table S4: Typical 3D Model parameters, unless otherwise stated

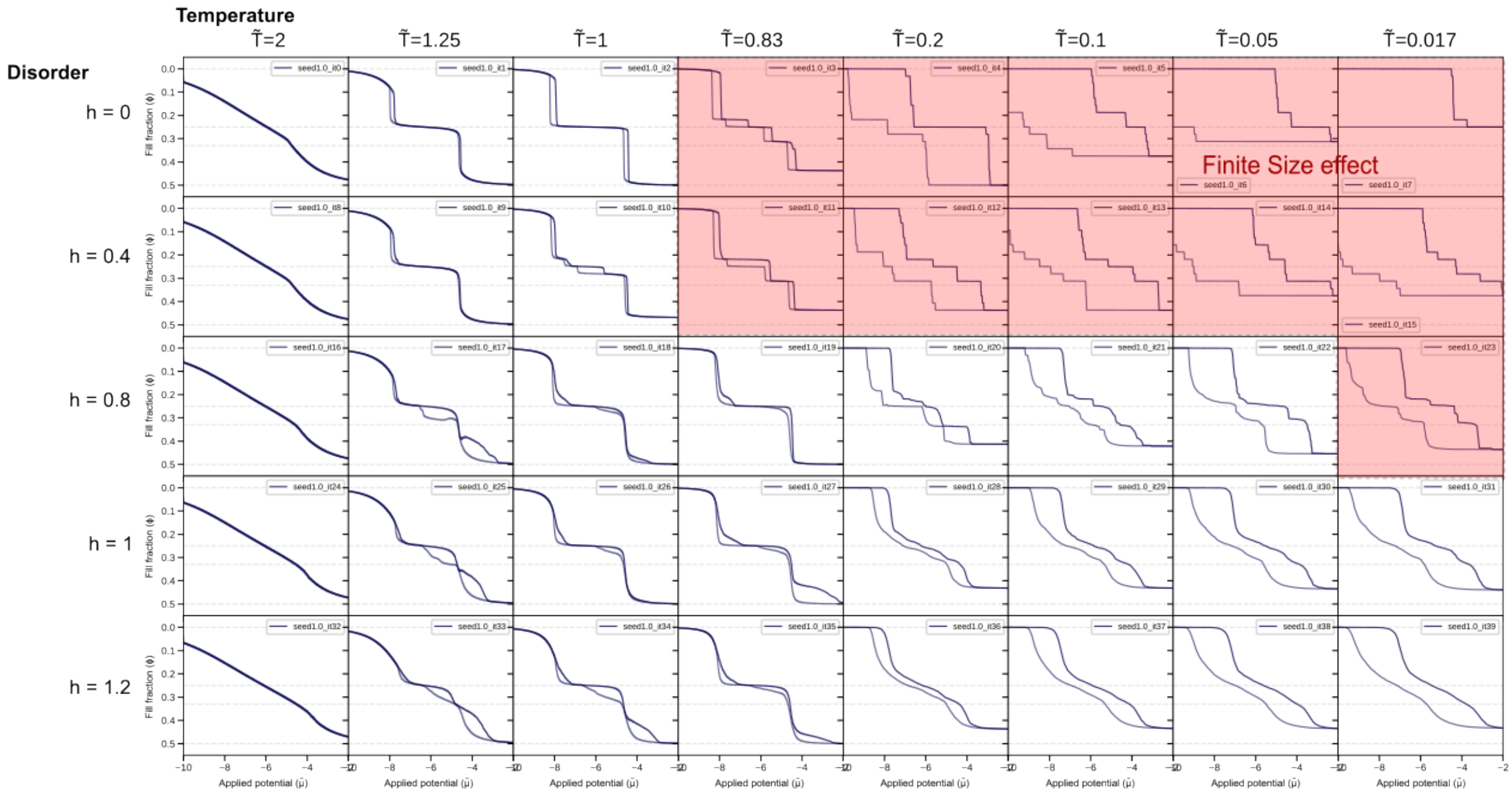

Figure S16 | 3D model behaviour over disorder-temperature space. Parameters can be found in Table S4. Parameters can be found in Table S4. Similar to the 2D model, at low disorder strengths and low temperatures, the correlation length in the xy directions increase, and when this correlation length exceeds the xy lattice size, the entire layer fills at once. This finite size effect is indicated by the red squares.

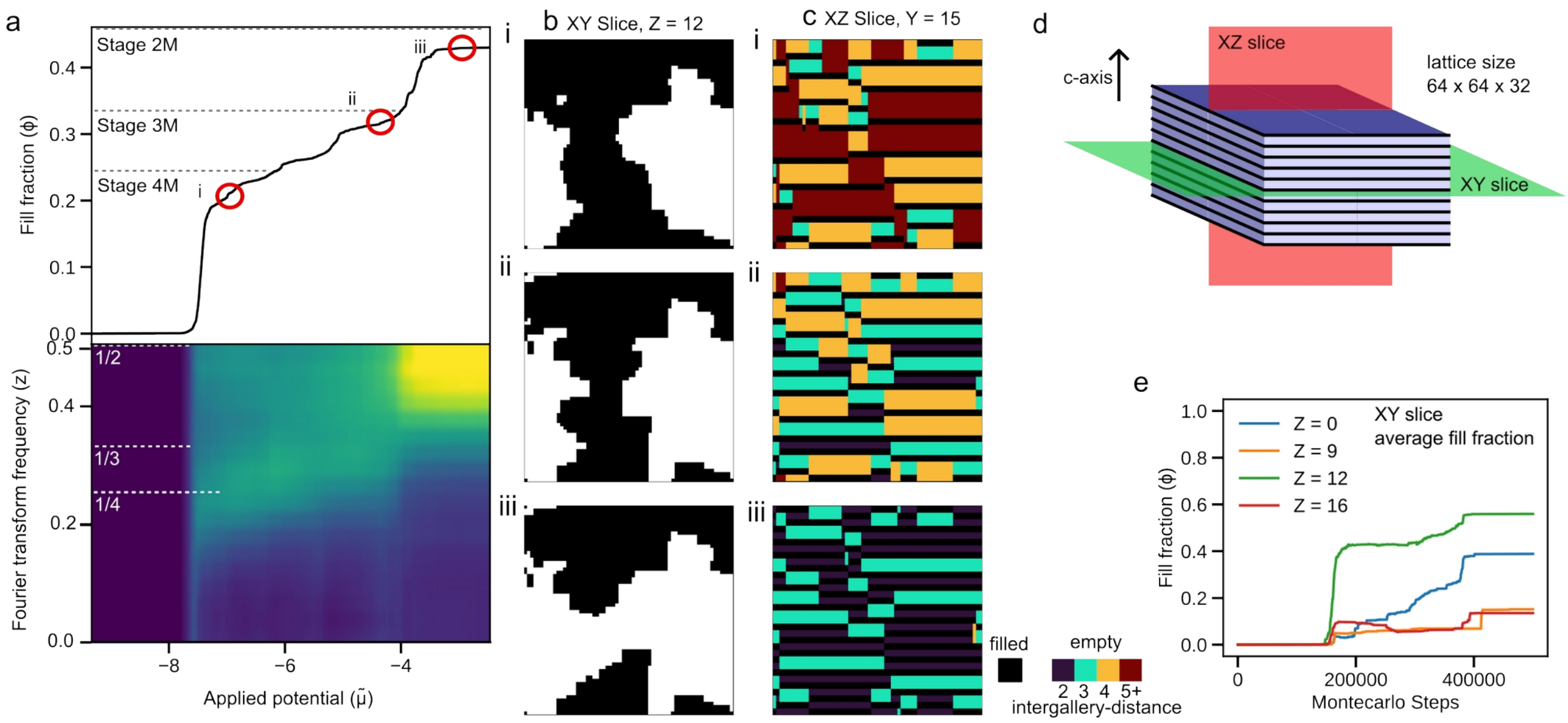

Figure S18 | Example of lattice evolution during 3D simulation. a, The fill fraction during a filling simulation (upper panel) with time points of interest marked with a red circle. As discussed in the main text, Stage 4M, 3M, 2M indicate the disordered mixture of staging distances which, on average, appear to have 1/2, 1/3 and 1/2 filling. A 3D Fourier transform of the lattice is averaged in the kx and ky direction, and its evolution over the simulation is shown as a heat map (lower panel).

b, An XY slice at Z = 12, and **c,** an XZ slice at Y = 15, are shown at various times as marked in (a). **d,** A schematic demonstrating an XY and XZ slice in the lattice. **e,** A selection of XY slices is extracted, and their individual mean fill fraction is plotted vs simulation time. Intermediate temperature ($\tilde{T}$ = 0.1) and disorder ($\tilde{h}$ = 0.9), with 500k total Monte Carlo steps in 64 x 64 x 32 lattice size was used for the simulation.

R4.6.2: *Additionally, the disorder is defined as site-specific rather than expressed through variations in the coupling constants between layers, which could lead to qualitatively similar behaviour.*

The reviewer suggests expressing disorder in terms of a variation in the coupling constant. This is a good suggestion and has been well studied in the past in the form of the Random Bond Ising model (RBIM). However, as mentioned by the reviewer, it retains many of the same qualitative features as the Random Field Ising model (RFIM) (Vives & Planes, 1994) and furthermore, as discussed in response R1.4, the origin of disorder in graphite may be a widespread elastic deformation resulting in a site variation in lithium occupation energy, meaning that the disorder model (Gaussian random field) used here may be appropriate. Nonetheless, this is an interesting suggestion, and we have added this to the discussion in the Supplementary Section 2.4. (please refer to R1.10)

R4.6.3: *It would strengthen the work if the authors perform a more comprehensive analysis regarding system size effects, as the current model is too small to draw conclusions that can meaningfully compare to experiments. Given the standards of a Nature publication, I would expect a much more extensive computational study that goes beyond a simple 2D Ising-like model to include 3D systems and, ideally, extensions toward phase-field-type models.*

The reviewer also notes system size effects and the use of a more complex model. As for the system size effect, a single lattice site in the model does not represent a single lithium ion in our model, but a coarse-grained region in the xy direction. To clarify, we have edited the Supplementary modelling section:

The dynamics involves individual spins changing their state, which corresponds to Li ions filling or emptying a region within a single gallery in the system.

In addition, we have explored lattice sizes of up to (x,y)=(500,80) in 2D, and (x,y,z)=(128,128,64) in 3D, which is comparable in the z-direction to our experimental penetration depth of ~100 layers. The simulation results with larger lattice sizes were similar to the simulations presented in *Supplementary Section 2.1 and 2.2*.

The reviewer also mentions the use of a more complex, continuum model. We would like to highlight that a key message in our paper is that complex and unconventional dynamics that we observe in graphite is actually well understood in the field of disorder-driven non-equilibrium first order transitions, in which RFIM is prototypical. (Also refer to *R3.5* for more discussion)

In fact, while most used for avalanches in magnetic systems (Barkhausen noise)(Durin & Zapperi, 2004; Kuntz et al., 1999; Sethna et al., 1993, 2001), RFIM has found widespread use in the field of disordered phase transitions, and has been applied to understand avalanches dynamics in Martensitic transitions(Kustov et al., 2023), condensation in disordered media(Aubry et al., 2014; Detcheverry et

al., 2005) and ferroelectrics(Casals et al., 2021; Nattermann, 1990), as well as for understanding disordered phase transitions such as Jahn-Teller transitions(Graham et al., 1987) and metal-hydrogen systems(Fenzl & Peisl, 1985) .

For example, Martensitic transformations are a complex crystallographic transformation with avalanches arising due to dynamically formed dislocations, defects and domain boundaries. Yet RFIM, with its discrete spin-flips and quenched field disorder, has been a dominant model system for studying Martensitic transformations. This is because, despite its simplicity, RFIM captures many complex emergent phenomena arising in disordered non-equilibrium phase transitions, such as intermittent avalanches, its scale-free distribution close to criticality, morphology of avalanche clusters(Ji & Robbins, 1991; Kuntz et al., 1999; Walsh et al., 1998) and return point memory (Sethna et al., 1993).

Our current model is able to capture many qualitative features of the experiment, and we believe it lays groundwork for a continuum, phase field extension in the future. We have discussed the model limitations previous (please refer to *R1.10*). Instead, we have better highlighted other qualitative features that is captured in the model, adding a paragraph in the main text, with further discussions added to *Supplementary Section 2.3.*

Main:

Importantly, our model can explain many qualitative features of the experimental data. For example, in Extended Data Figure 3a, the model demonstrates the disorder rounding the transition causing a broader temporal spread of avalanches, similar to the broader spread of step events occurring in more disordered region D (Fig. 2c). Additionally, in Extended Data Figure 3b,c, we show that in these regions, smaller avalanches become more common, in agreement with the model and the expected disorder trend from previous work[32,45] . In fact, this generalises to other graphite samples with different levels of disorder (Supplementary Section 1.2.3), where more disordered graphites such as MCMB1028 displays smaller step sizes and vice versa. There is also a driving rate (C-rate) dependence, with faster driving resulting in overlapping of avalanches which eventually become difficult to distinguish[38,46] (Supplementary Section 1.2.2).

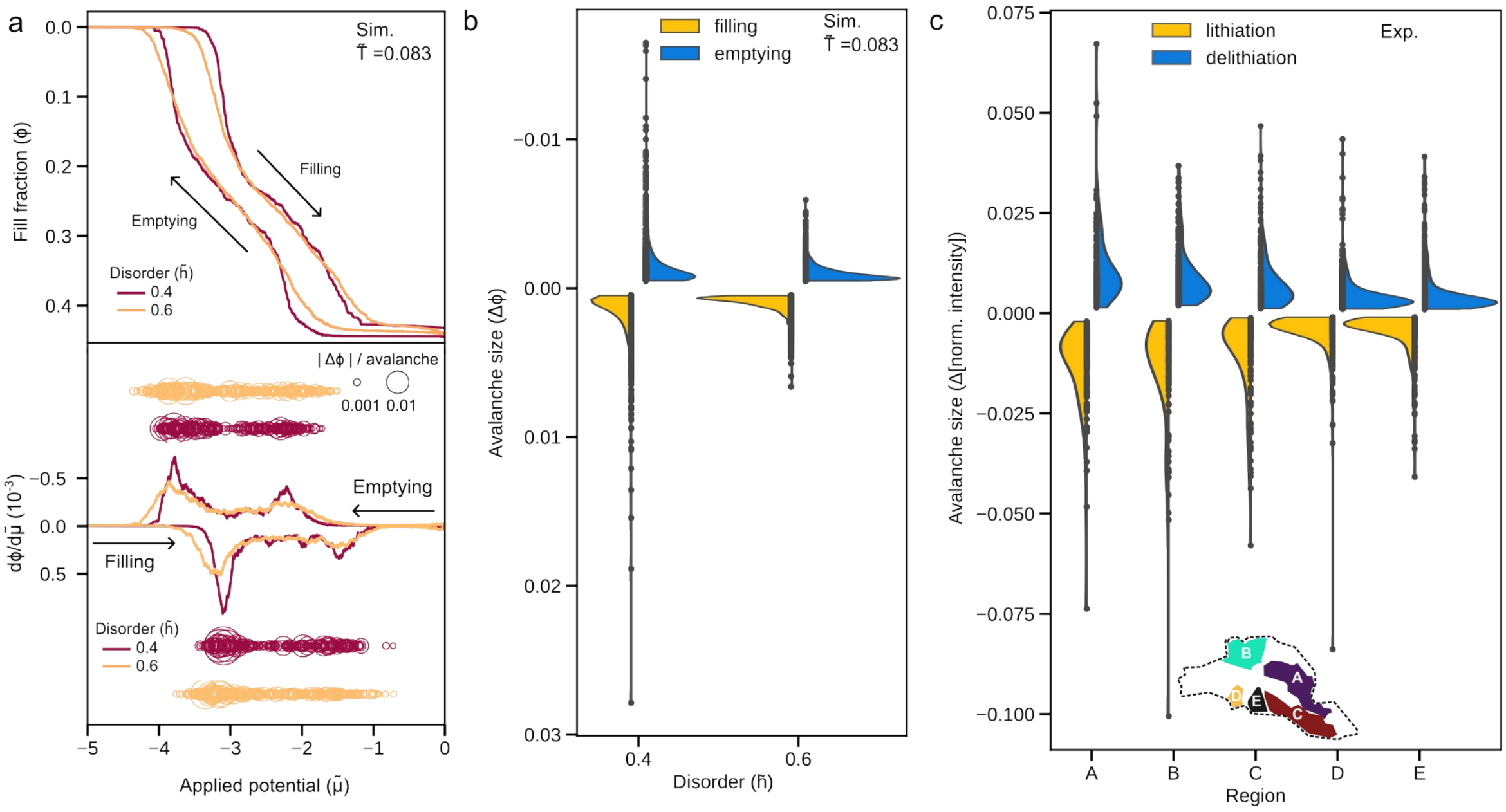

Extended Data Figure 3 | Effect of disorder on model trends, compared to avalanches distributions from different regions. a, Mean lattice fill fraction (ϕ) vs applied potential ($\tilde{\mu}$) during filling and emptying simulations for two different extents of disorder for the 2D model ($\tilde{T} = 0.083$, mcs = 80k) for a single run (upper panel). The differential fill fraction per potential ($d\phi/d\tilde{\mu}$) during the filling/emptying simulations for corresponding filling/emptying simulations with different disorder (lower panel). Above and below the differential plots are the corresponding detected avalanche events, plotted as a circle with its x position denoting the potential at which it occurred. **b,c,** Violin plots showing the detected avalanche size distribution with different disorder strengths and regions. Statistics from model (**b**), gathered from a single filling/emptying run with varying disorder strengths from 2D model ($\tilde{T} = 0.083$, mcs = 80k), where size is defined as the change in mean fill fraction. Statistics from experiment (**c**) from different regions during de/lithiation cycles at all temperatures, where size is defined as change in the mean optical intensity. A map of the particle regions is located at the bottom for reference. The Y-axes has been reversed in order to aid comparison of simulation to experiment

Supplementary 2.3: Comparison to Experimental features

For the 2D model, the filling/emptying trend and avalanche size distribution is plotted as a function of temperature in Fig. S19a,b, and demonstrates consistency with previous studies in standard RFIM(Yao & Jack, 2023) where at higher temperatures, (1) large (high $\Delta\phi$) avalanches occur less frequently and (2) smaller avalanches occur more frequently, due to thermal fluctuations exciting

smaller avalanches and consequently preventing the build-up required for large avalanches to occur. However, note the experimental trend (Fig. S20) does not change substantially, which is due to the experimental temperature range (T = 288 K to 318 K, approx. 10% increase) being far smaller than that presented in the model ($\tilde{T}$ = 0.042 to 0.125, approx.. 300% increase in Fig. S19b). Over a similarly narrow temperature range (Fig. S19c), a trend in the avalanche size distribution is too small to be visible in the simulation statistics. Similarly, there is only small changes in the hysteresis over this narrow range, which indicates that the temperature trend in the experimental voltage hysteresis is related to reaction overpotential and mass transport, with a relatively constant zero-current hysteresis.

Interestingly, higher temperatures lead to thermal-fluctuation induced back-hopping, leading to step changes in the opposite direction of the driving force. These reverse hops are evident even within a narrower temperature window, and shown in Fig. S21a, where the total number of avalanches increases with temperature along with an increase in the number of reverse hops. We also experimentally detect these reverse hops, where a local region seemingly delithiates during a lithiation cycle and vice versa as demonstrated in Fig. S22. These reverse hops become more frequent at higher temperatures (Fig. S21b), supporting the idea that the avalanches are thermally activated jumps between shallow metastable states. Critically, it implies that we are in a regime where thermal fluctuation, the disorder strength and long-range coupling energies are all relevant.

The non-equilibrium transition pathway demonstrated by the simulations can give rise to asymmetry in the filling vs emptying behaviour. This is seen in Fig. S20a, lower panel, where the differential fill fraction (equivalent to the experimental dQ/dV curve) is calculated over 140 simulation runs, and shows 4 peaks during filling, and 3 during the emptying simulations. This originates from the fact that we have a mixture of many intergallery distances which become un/stable at certain potential ranges, but due to disorder, this potential range is broad and overlaps significantly unless slowly ramped. This is illustrated in Figure S23. The experiment dQ/dV asymmetry is reverse, with 3 peaks during lithiation and 4 during delithiation. Nonetheless, the results may differ with a slower simulation in a 3D lattice, and we speculate that similar effect may cause asymmetry in the experiment.

Finally, as discussed in the main text, each avalanche in the model is usually a result of a small domain in a single layer filling. This is demonstrated in the 3D model case, in Figure S24, where a few selected avalanche events are shown as a lattice differential in a 3D and 2D map. Each step corresponds to a connected cluster in a single layer filling up, where the cluster size and morphology varies widely.

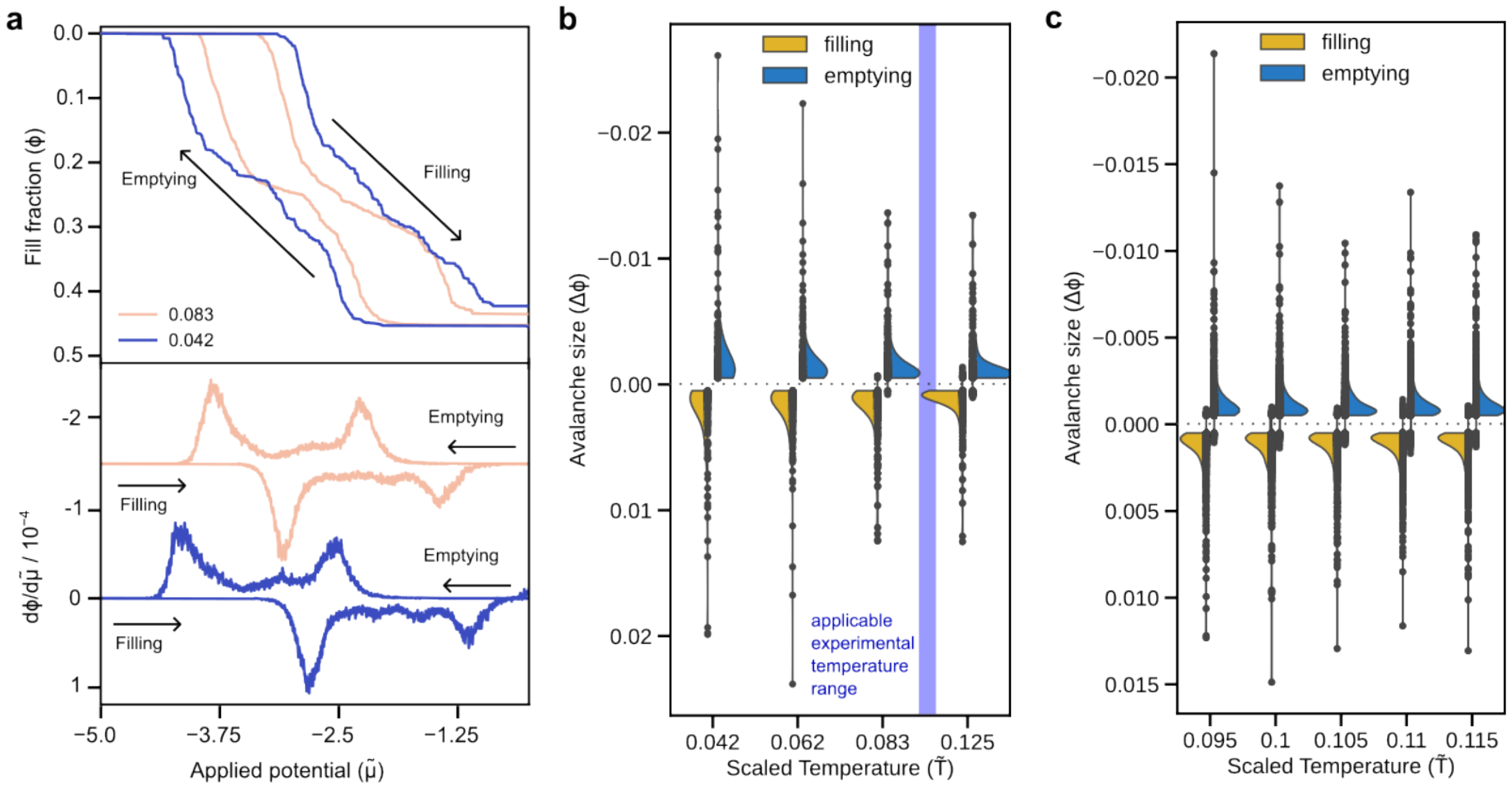

Figure S19 | Detailed hysteresis curve analysis and avalanche statistics from 2D model. a, Mean lattice fill fraction (ϕ) vs applied potential ($\tilde{\mu}$) during filling and emptying simulations at different temperatures for the disordered model ($\tilde{h}$ = 0.4, mcs = 80k) for a single run (upper panel). The differential fill fraction per potential ($d\phi/d\tilde{\mu}$) during the filling/emptying simulations for the corresponding temperatures (lower panel). The differential signal was calculated from an average of 140 runs using a low pass filter (Butterworth, 1/50). **b,c,** Violin plots showing the detected avalanche size distribution at different temperatures with dotted line drawn at zero intensity. Over a wide (~300% increase) temperature range (a), and a narrower (~20% increase) temperature range, corresponding better to the experimental temperature range. Statistics from a single filling/emptying run per temperature in the disordered model ($\tilde{h}$ = 0.4, mcs = 80k), where size is defined as the magnitude of the change in average fill fraction. Y-axes has been reversed in order to aid comparison of simulation to experiment.

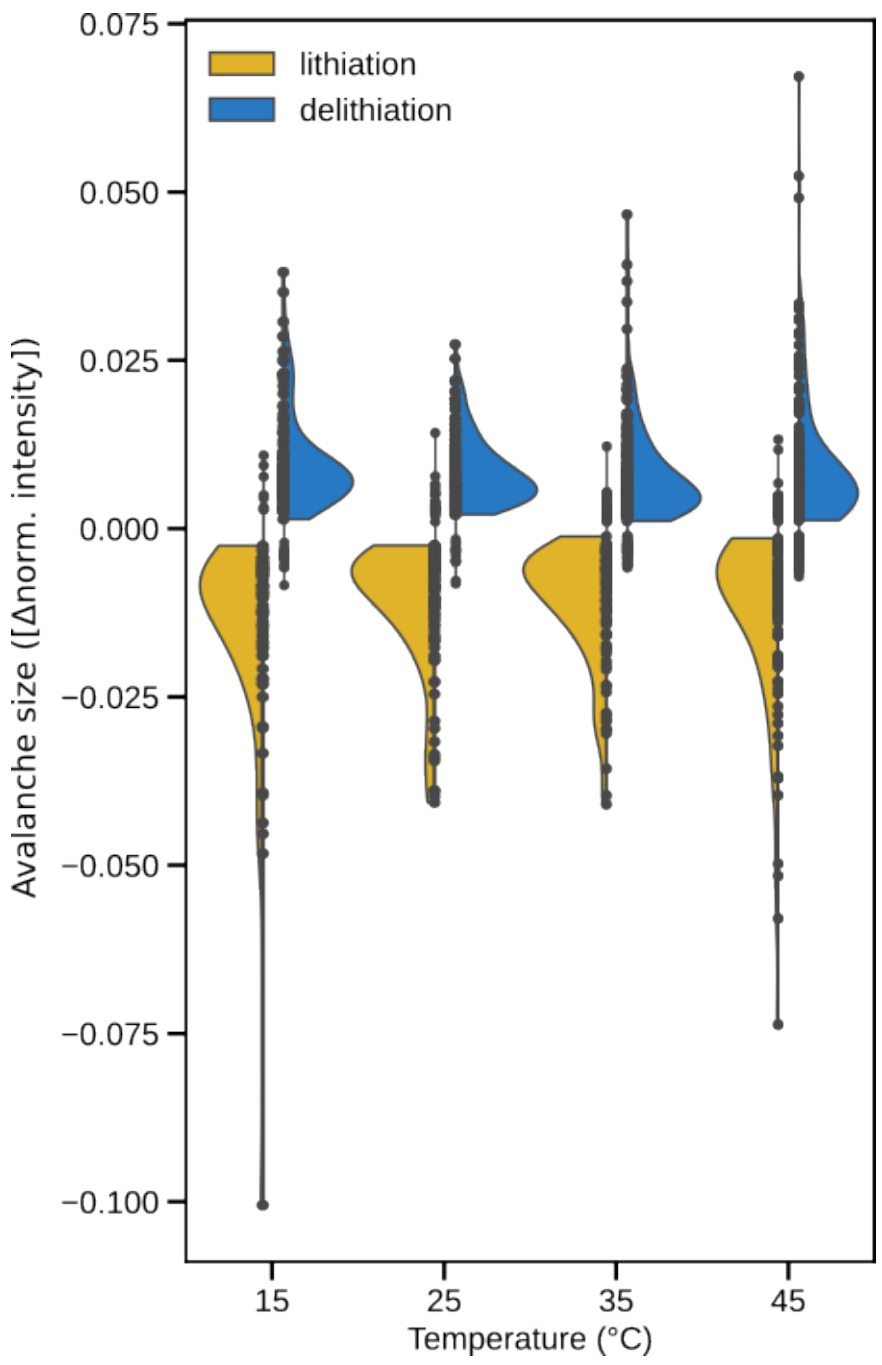

Figure S20 | Avalanche statistics from experiment. Violin plots showing the detected avalanche size distribution at different temperatures. Statistics from experiment during cycles 1 and 2, with de/lithiation at all temperatures, where size is defined as change in the mean optical intensity. The experiment statistics were collected only from the 1L-4L window, from regions A, B and C which have similar levels of disorder.

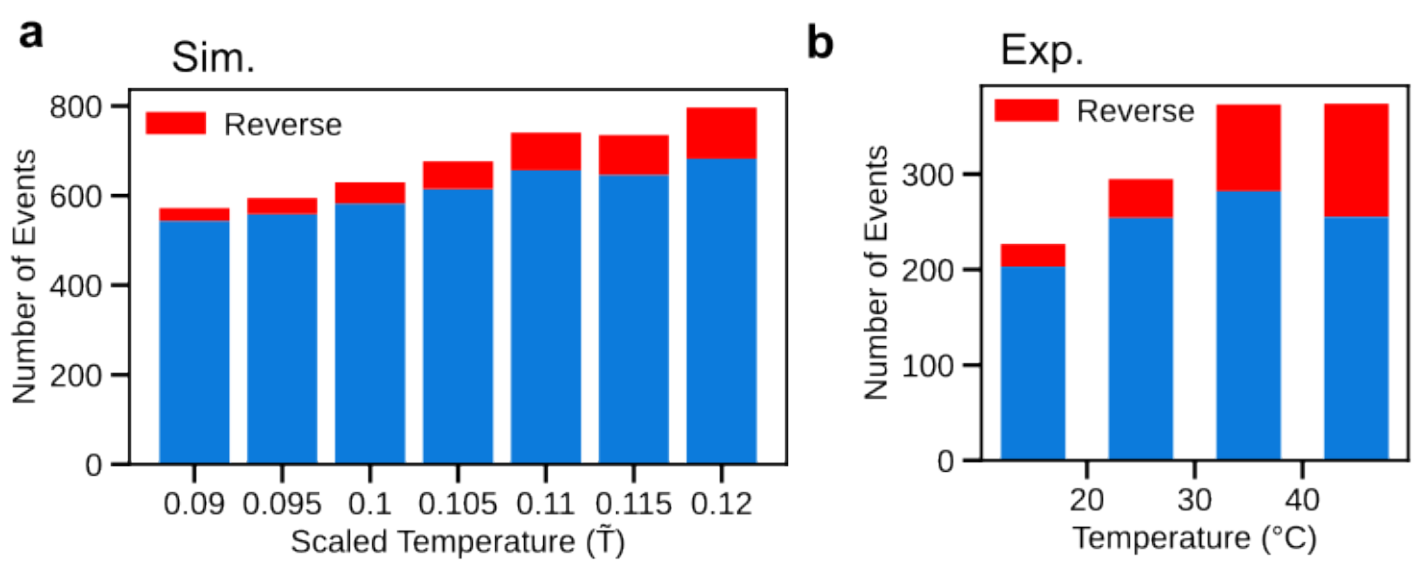

Figure S21 | The number of detected avalanches. The number of detected avalanches as a function of temperature, with the fraction corresponding to reverse events (thermal-fluctuation back-hopping) marked in red. From the 2D model (**a**) and the experiment (**b**). Total number per cycle, per run. The model corresponds to the same dataset shown in Fig. S19. The experiment statistics were collected only from the 1L-4L window, from regions A, B and C which have similar levels of disorder.

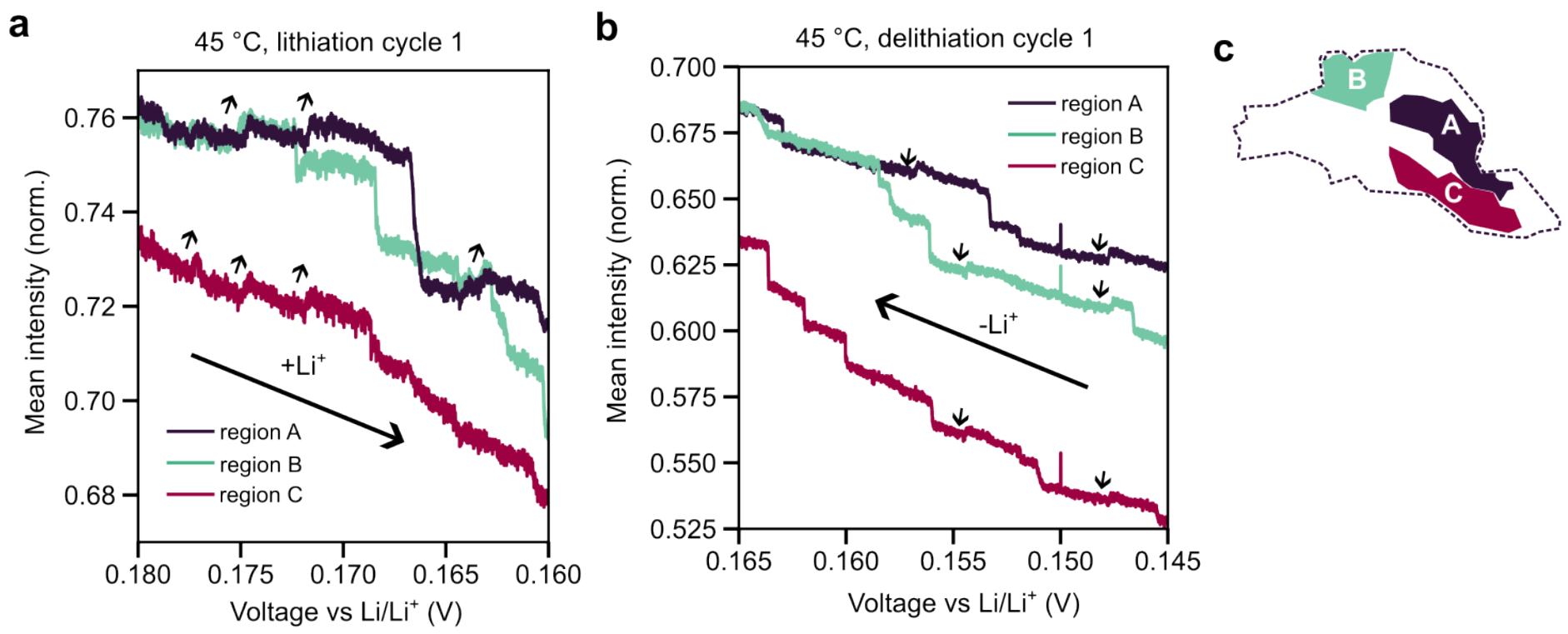

Figure S22 | Illustration of experimental reverse avalanches. Mean intensity (normalised) vs Voltage shown for three selected regions during 45 °C (a) lithiation and (b) delithiation. Both de/lithiation cycles and all regions show "reverse avalanche" behaviour, where there is a step up (during lithiation) or step down (during delithiation), going against the overall trend. These small steps are marked with an arrow.

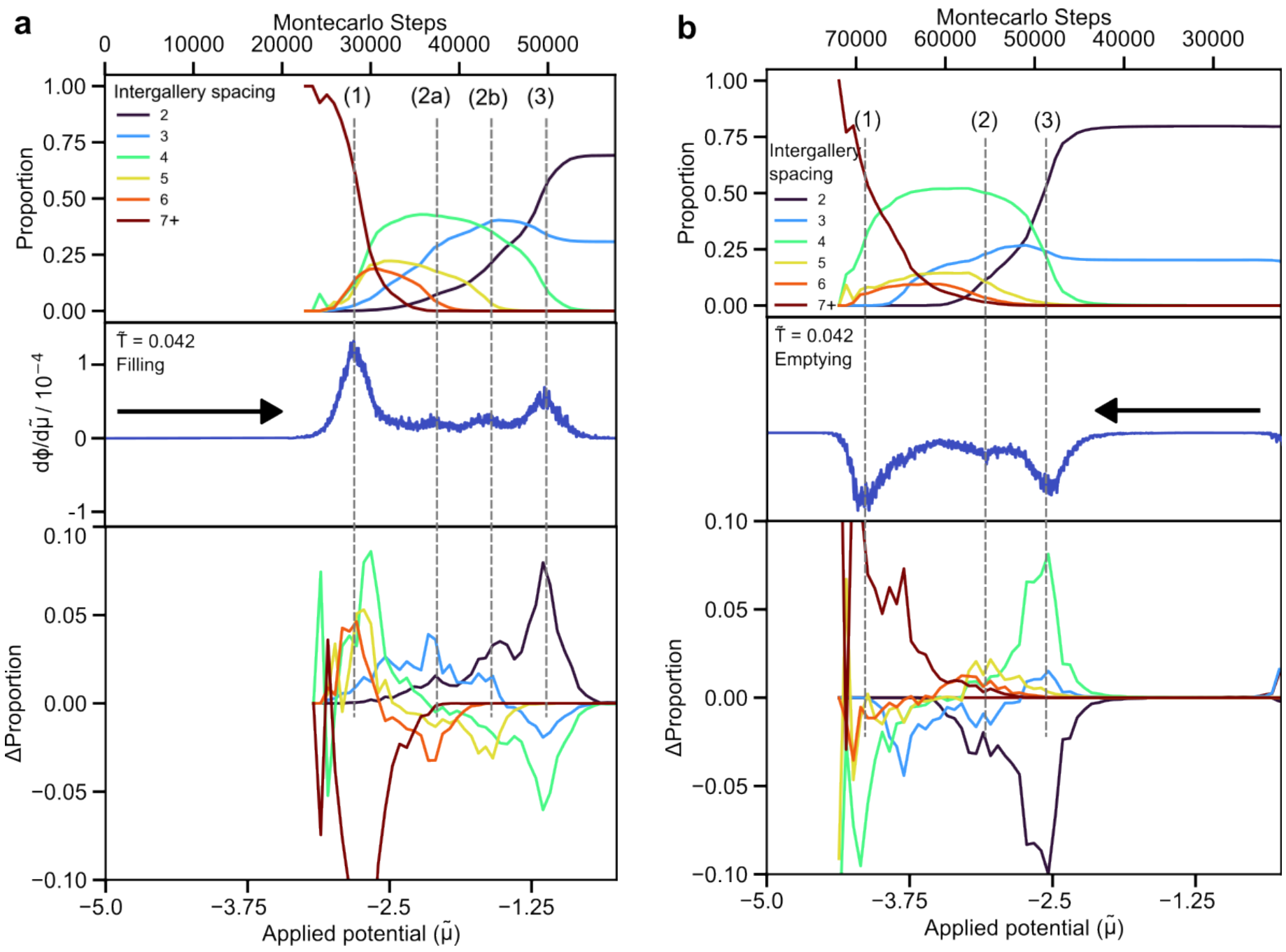

Figure S23 | Population dynamics of intergallery spacing in the model. a,b, Staging population dynamics during filling (**a**) or emptying (**b**) at $\tilde{T} = 0.043$, $\tilde{h} = 0.4$. Statistics gathered over 140 runs. The top panel shows the population of intergallery spacing vs time. The intergallery spacing is defined as the vertical distance between filled lattice sites in the model. Every empty lattice blocks is classified as either 2, 3, 4, 5, 6, or 7+ according to the distance between the respective filled sites. The middle panel shows the overall differential filling curve, with dotted vertical line drawn at peak positions. The bottom panel shows the time differential of different spacing populations. Small peaks in the differential filling curve can be interpreted as a result of population dynamics. For example, peak 3 in (**b**) could be interpreted as the conversion from Stage 3 and 4, to Stage 2 spacing.

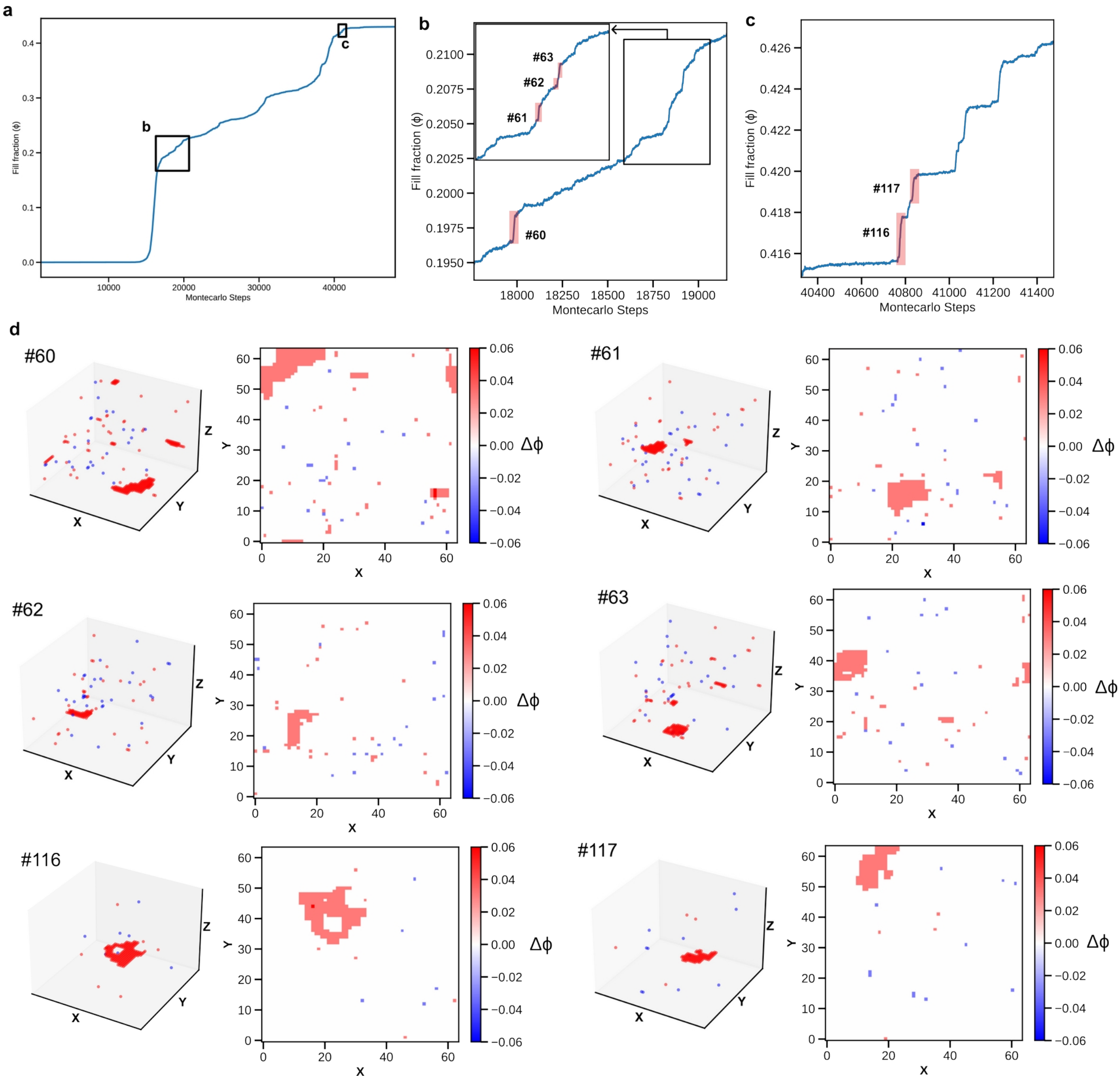

Figure S24 | Origin of avalanches in 3D model. a, Mean fill fraction curve during filling simulation from 3D model same as shown in S18. Small squares indicate areas selected for the zoom-ins. **b,c,** Zoomed-in area showing clear avalanche steps. Red squares highlight single avalanches, which are numbered. **d,** Lattice picture of highlighted and numbered avalanches from (b) and (c). The change in lattice occupation during a single detected avalanche is calculated and shown in a 3D schematic (left) or a 2D XY averaged map (right). Red indicates filling and blue indicates unfilling.

R4.6.4: *Such an effort would connect the present work to two decades of literature on graphite modelling and provide a more complete physical picture.*

The reviewer also highlights the lack of links to previous literature of graphite intercalation with the current model. In response, we would like to highlight work by Schon and Dresselhaus (Schon et al., 1988) which established the static phase diagram of staging, starting from an Ising model analogue with the same attractive intra and repulsive interlayer coupling terms. We adapted the screened

interlayer repulsion term from recent dynamic simulation with multilayer phase field models(Chandesris et al., 2019; Smith et al., 2017),. Finally, disordered and mixed higher order staging in graphite has been shown previously by Kirczenow through Monte Carlo simulations(Kirczenow, 1985, 1988).

To further highlight these links in the main text, we have added references to previous work in the main text:

Main

... with attractive first-neighbour intralayer coupling, up to third-nearest neighbour repulsive interlayer coupling with screening[39–41]

... similar discrete modelling has shown graphite to possess disordered, heterogeneous phases with complex and metastable transition dynamics[24,42].

R4.6.5: *Lastly, it was never mentioned how the authors chose the rate at which they varied the baseline chemical potential in their model.*

Finally, the chemical potential ramp rate was chosen to be slow enough to be close to the quasi-static limit without being excessive. The avalanches appearing in the simulations are clearly separated and follows the expected trend in its filling and hysteresis curve. We have added a Supplementary figure which demonstrates effect of driving rate in the model, *Figure S17 in Supplementary Section 2.2.* It is interesting to note that the experiment is not at the quasi-static limit either, but close to it (as discussed above in R4.1).

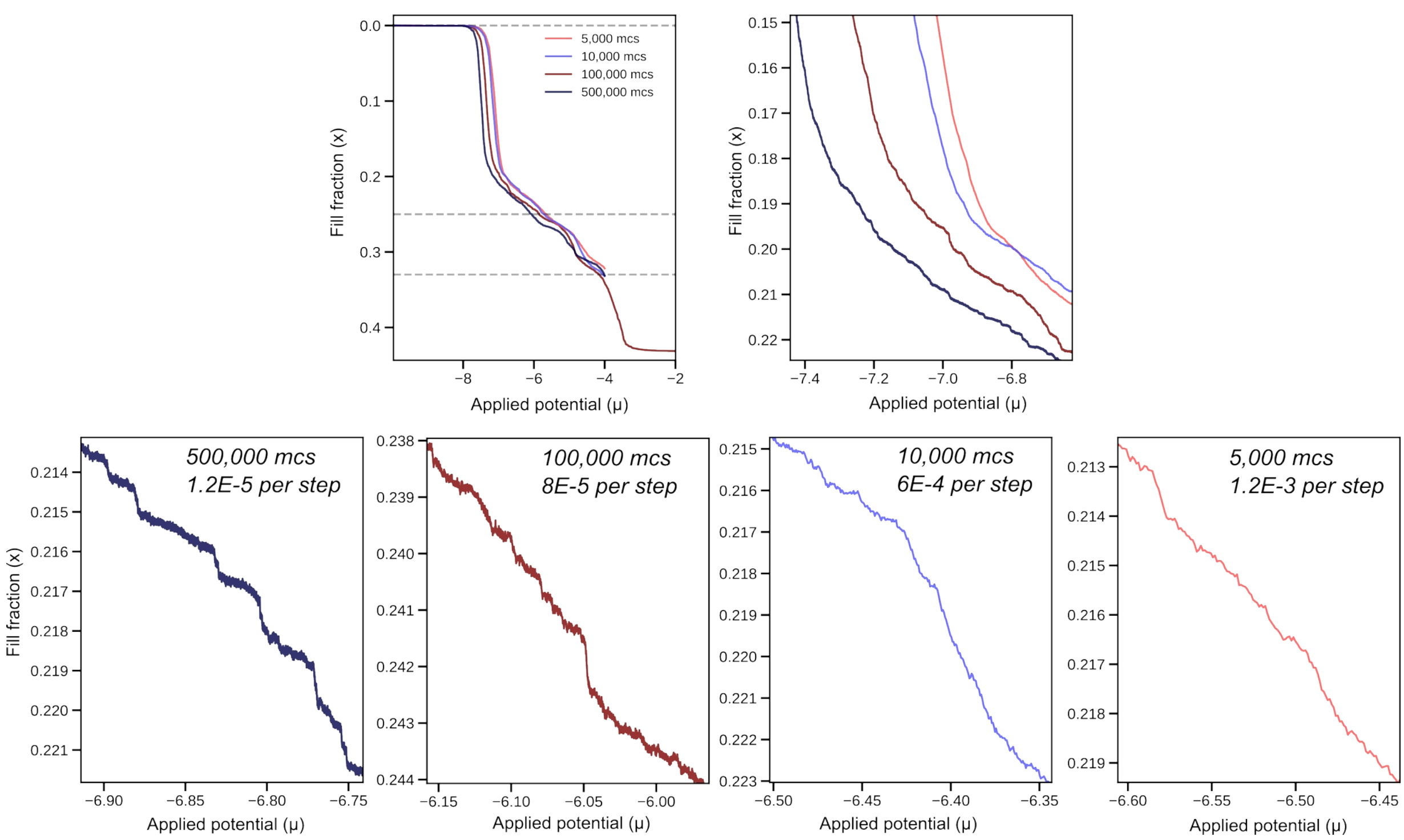

Figure S17 | Driving rate dependence of model. Temperature ($\tilde{T}$) = 0.1, disorder ($\tilde{h}$) = 1. Other parameters as shown in Table S4.

R4.7: *In connection to comment (5) and the last paragraph of the introduction:*
'Finally, using the cross-correlation function as an effective way of analysing the stochastic filling events, we demonstrate that the seemingly random sequence of avalanches revealed in our optical experiments possess definite spatio-temporal correlations patterns, related to the regions and disorder identified in the electron backscatter diffraction (EBSD) map.'

This finding provides a strong motivation for pursuing a more comprehensive computational analysis to understand how lithiation and delithiation progress in quenched disordered systems, rather than limiting the discussion to the correlation level of experimental observations.

We are glad to hear that the reviewer share our excitement for the spatio-temporal correlation analysis. We agree that a computational study on spatio-temporal correlation would be highly interesting, and a natural follow up. We stress that we now have a good experimental understanding of the nature of disorder on the particle, which relates directly to the inter-region connectivity calculated from the correlation analysis (Please refer to *R1.4*). In addition, we have described how these correlations might be studied computationally in *Supplementary Section 2.4* (please refer to *R1.10*). Our future work will include a more extensive use of spatio-temporal correlation analyses based on a larger dataset, which we hope will give a better foundation for launching a computational study.

R4.8: *There are several typos in the SI of the paper. I would suggest a more careful proofreading of the submitted material. It would also be very helpful to include a complete reference list in the SI, as several papers are not properly cited and I had to infer which works the authors refer to.*

We thank the reviewer for the catching these errors. The Supplementary Information has been rewritten, with all references included to the best of our knowledge.

R4.9: *It would be great to quantify the ion fractions corresponding to the dilute regime, both in the introductory paragraph and throughout the text. This would help readers directly relate the observed phenomena to the corresponding stored energy.*

This is a great suggestion. We have changed *main text Fig. 1* to include ion fractions (please refer to *R1.7*) and have referred to it in the main text.

R4.10: *In the sentence 'Phase transitions in battery materials are often categorized as a solid-solution 12,13 or phase separating 14--17, where lithiation in graphite has been reported to have both first-order biphasic transitions and 'pseudo' solid-solutions 9,18,19,' I have two comments:*
i) I do not believe this phrasing is precise. How can a phase transition itself be categorized as a solid solution?

ii) I do not think the cited works explicitly establish the terminology of 'pseudo solid-solution,' so it is a bit difficult to interpret in the broader context of phase transitions.

(i) The reviewer is correct here, describing a phase transition as a solid-solution is not strictly correct. We have changed the phrasing to:

De/lithiation processes in battery materials are often categorized as continuous[12–15] or phase separating[16–19],

...we observed first-order biphasic behaviour[24] or solid-solution diffusion[25,26] within cathode particles during intercalation...

(ii) The reviewer is also correct, the Stage 4L-3L transition has been described as resembling a solid-solution(Mercer et al., 2021) or as an "apparently" continuous transition(J. R. Dahn, 1991; Didier et al., 2020). This language was previously used because of the inherent contradiction between the observation of distinct phases by bulk techniques such as diffraction, and smooth electrochemistry profile and diffraction peak shifting. Given that the prior work did not use the phrase "pseudo solid solution" our revised text now states:

...as well as reproducing the apparent continuous behaviour of the 4L-3L transition...

Subsequently, we observed a seemingly continuous drop in optical intensity during the transition 4L→3L...

R4.11: *In the sentence 'This is fundamentally different from our previous optical microscopy studies where we observed first-order biphasic behaviour 24 or solid-solution diffusion 25,26 within cathode materials,' it would be helpful to explicitly mention the specific chemistries studied previously.*

This is a good suggestion, we have changed the manuscript as follows:

...where we observed first-order biphasic behaviour in Li_xCoO_2[26] and solid-solution diffusion in $LiNi_xMn_yCo_zO_2$[27,28] single particles during intercalation.

R4.12: *In Fig. 3b, it would be useful to include an explicit schematic illustrating the definition of the inter-gallery distance. It took me some time to distinguish the white lines and understand the coloring. Moving the schematic from Extended Data Fig. 2a into Fig. 3b would greatly improve clarity for readers.*

We apologies for the confusing colour scheme. As per suggestion, we have moved the schematic from the extended figure to *Figure 3* as follows.

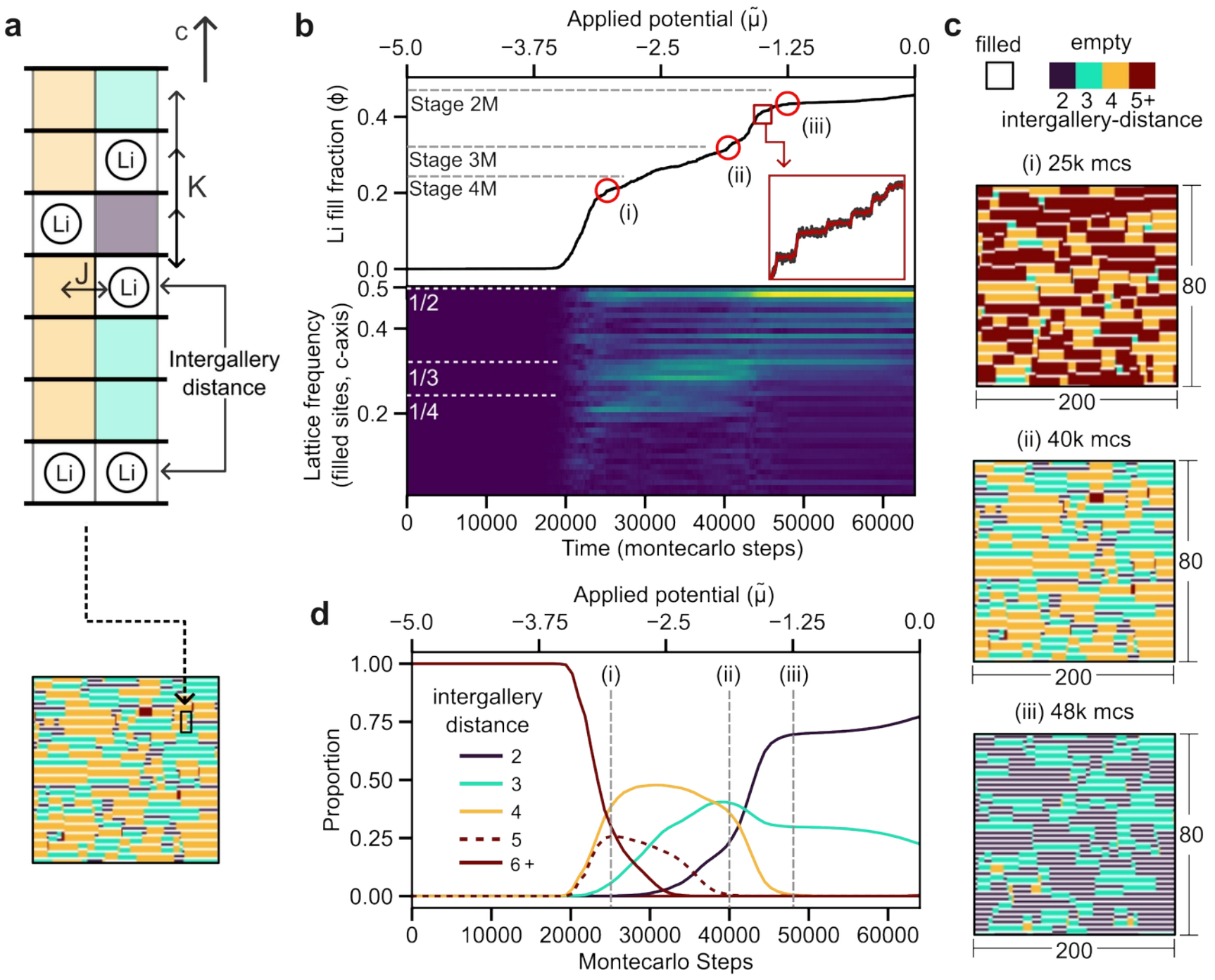

Figure 3 | Disordered lattice model (random field Ising-like) filling behaviour. a, Schematic of 2D model coupling and terminologies. b, Mean lattice fill fraction (ϕ) vs Monte Carlo steps (mcs) during a single ramped potential simulation (upper panel), with disorder ($\tilde{h}$ = 0.4), and temperature ($\tilde{T}$ = 0.125), with total mcs = 80k. Red circles denote time points (i)-(iii) at which the lattice pictures in (c) are taken from. Red square inset highlights a portion of the filling curve which demonstrates stepped (avalanche) behaviour (mcs = 45.7k – 47.2k). The respective Fourier transform of the lattice in the c-direction vs Monte Carlo steps (lower panel). c, The lattice structure shown at different time points (i)-(iii). Occupied sites are drawn in white, and unoccupied sites are coloured according to the respective c-axis separation between occupied sites. c, Every c-direction gap between occupied sites are classified according to the distance, and the statistic is plotted as a population proportion vs time. 80 slices are taken from 0 to 80k mcs. The time points corresponding to (b) are marked with a dotted line (i),(ii),(iii). Results only shown up to 64k mcs ($\tilde{\mu}$ = 0) for clarity.

References:

Amengual, A., Picornell, C., Segui, C., & Torra, V. (n.d.). *SYSTEMATIC STUDY OF THE MARTENSITIC TRANSFORMATION IN A Cu-Zn-AI ALLOY. REVERSIBILITY VERSUS IRREVERSIBILITY VIA ACOUSTIC EMISSION*.

Aubry, G. J., Bonnet, F., Melich, M., Guyon, L., Spathis, P., Despetis, F., & Wolf, P.-E. (2014). Condensation of Helium in Aerogel and Athermal Dynamics of the Random-Field Ising Model. *Physical Review Letters*, *113*(8), 085301. https://doi.org/10.1103/PhysRevLett.113.085301

Baró, J., Planes, A., & Vives, E. (2016). Avalanche dynamics in driven materials. *CONTRIBUTIONS to SCIENCE*, *11*, 153–162. https://doi.org/10.2436/20.7010.01.226

Casals, B., Nataf, G. F., & Salje, E. K. H. (2021). Avalanche criticality during ferroelectric/ferroelastic switching. *Nature Communications*, *12*(1), 345. https://doi.org/10.1038/s41467-020-20477-6

Chandesris, M., Caliste, D., Jamet, D., & Pochet, P. (2019). Thermodynamics and Related Kinetics of Staging in Intercalation Compounds. *The Journal of Physical Chemistry C*, *123*(38), 23711–23720. https://doi.org/10.1021/acs.jpcc.9b05298

Chern, G.-W. (2017). Magnetic avalanches in granular ferromagnets: Thermal activated collective behavior. *Journal of Physics: Condensed Matter*, *29*(4), 044004. https://doi.org/10.1088/1361-648X/29/4/044004

Dahn, D. C., & Haering, R. R. (1982). Phase mixtures and staging in intercalated LixNbSe2. *Solid State Communications*, *44*(1), 29–32. https://doi.org/10.1016/0038-1098(82)90706-2

Dahn, J. R. (1991). Phase diagram of Li x C 6. *Physical Review B*, *44*(17), 9170–9177. https://doi.org/10.1103/PhysRevB.44.9170

Dahn, J. R., & Haering, R. R. (1981). Anomalous bragg peak widths in LixTiS2. *Solid State Communications*, *40*(3), 245–248. https://doi.org/10.1016/0038-1098(81)90750-X

Detcheverry, F., Kierlik, E., Rosinberg, M. L., & Tarjus, G. (2005). Helium condensation in aerogel: Avalanches and disorder-induced phase transition. *Physical Review E*, *72*(5), 051506. https://doi.org/10.1103/PhysRevE.72.051506

Didier, C., Pang, W. K., Guo, Z., Schmid, S., & Peterson, V. K. (2020). Phase Evolution and Intermittent Disorder in Electrochemically Lithiated Graphite Determined Using in Operando Neutron Diffraction. *Chemistry of Materials*, *32*(6), 2518–2531. https://doi.org/10.1021/acs.chemmater.9b05145

Dreyer, W., Jamnik, J., Guhlke, C., Huth, R., Moškon, J., & Gaberšček, M. (2010). The thermodynamic origin of hysteresis in insertion batteries. *Nature Materials*, *9*(5), 448–453. https://doi.org/10.1038/nmat2730

Durin, G., & Zapperi, S. (2004). *The Barkhausen effect* (No. arXiv:cond-mat/0404512). arXiv. http://arxiv.org/abs/cond-mat/0404512

Fenzl, W., & Peisl, J. (1985). Hydrogen in Metallic Alloys as an Example of a Lattice Gas with Random Field and Random Bonds. *Physical Review Letters*, *54*(19), 2064–2067. https://doi.org/10.1103/PhysRevLett.54.2064

Fraggedakis, D., Nadkarni, N., Gao, T., Zhou, T., Zhang, Y., Han, Y., Stephens, R. M., Shao-Horn, Y., & Bazant, M. Z. (2020). A scaling law to determine phase morphologies during ion intercalation. *Energy & Environmental Science*, *13*(7), 2142–2152. https://doi.org/10.1039/D0EE00653J

Graham, J. T., Maliepaard, M., Page, J. H., Smith, S. R. P., & Taylor, D. R. (1987). Random-field effects on Ising Jahn-Teller phase transitions. *Physical Review B*, *35*(4), 2098–2101. https://doi.org/10.1103/PhysRevB.35.2098

Heß, M., & Novák, P. (2013). Shrinking annuli mechanism and stage-dependent rate capability of thin-layer graphite electrodes for lithium-ion batteries. *Electrochimica Acta*, *106*, 149–158. https://doi.org/10.1016/j.electacta.2013.05.056

Ji, H., & Robbins, M. O. (1991). Transition from compact to self-similar growth in disordered systems: Fluid invasion and magnetic-domain growth. *Physical Review A*, *44*(4), 2538–2542. https://doi.org/10.1103/PhysRevA.44.2538

Kirczenow, G. (1985). Kinetics of Stage Ordering and Stage Transitions. *Physical Review Letters*, *55*(25), 2810–2813. https://doi.org/10.1103/PhysRevLett.55.2810

Kirczenow, G. (1988). The kinetics of staging and stage disorder. *Synthetic Metals*, *23*(1–4), 1–6. https://doi.org/10.1016/0379-6779(88)90453-5

Kortan, A. R., Erbil, A., Birgeneau, R. J., & Dresselhaus, M. S. (1982). Commensurate-Incommensurate Transition in Bromine-Intercalated Graphite: A Model Stripe-Domain System. *Physical Review Letters*, *49*(19), 1427–1430. https://doi.org/10.1103/PhysRevLett.49.1427

Kuntz, M. C., Perkovic, O., Dahmen, K. A., Roberts, B. W., & Sethna, J. P. (1999). Hysteresis, Avalanches, and Noise: Numerical Methods. *Computing in Science & Engineering*, *1*(4), 73–81. https://doi.org/10.1109/5992.774844

Kustov, S., Nikolaev, V. I., Timashov, R. B., Kustov, B., Sapozhnikov, K., Pulnev, S. A., Yakushev, P. N., Masdeu, F., Wang, X., & Van Humbeeck, J. (2023). Crossover from mechanical stabilization of martensite to spanning avalanche-type reverse transformation of deformed Cu-Al-Ni shape memory crystals. *Journal of Alloys and Compounds*, *948*, 169751. https://doi.org/10.1016/j.jallcom.2023.169751

Mercer, M. P., Peng, C., Soares, C., Hoster, H. E., & Kramer, D. (2021). Voltage hysteresis during lithiation/delithiation of graphite associated with meta-stable carbon stackings. *Journal of Materials Chemistry A*, *9*(1), 492–504. https://doi.org/10.1039/D0TA10403E

Nattermann, Thomas. (1990). Random field systems. *Ferroelectrics*, *104*(1), 171–181. https://doi.org/10.1080/00150199008223821

Nixon, D. E., & Parry, G. S. (1968). Formation and structure of the potassium graphites. *Journal of Physics D: Applied Physics*, *1*(3), 303. https://doi.org/10.1088/0022-3727/1/3/303

Onuma, H., Kubota, K., Muratsubaki, S., Ota, W., Shishkin, M., Sato, H., Yamashita, K., Yasuno, S., & Komaba, S. (2021). Phase evolution of electrochemically potassium intercalated graphite. *Journal of Materials Chemistry A*, *9*(18), 11187–11200. https://doi.org/10.1039/D0TA12607A

Pérez-Reche, F. J., Truskinovsky, L., & Zanzotto, G. (2009). Martensitic transformations: From continuum mechanics to spin models and automata. *Continuum Mechanics and Thermodynamics*, *21*(1), 17–26. https://doi.org/10.1007/s00161-009-0096-2

Pérez-Reche, F.-J., Truskinovsky, L., & Zanzotto, G. (2007). Training-Induced Criticality in Martensites. *Physical Review Letters*, *99*(7), 075501. https://doi.org/10.1103/PhysRevLett.99.075501

Perković, O., Dahmen, K. A., & Sethna, J. P. (1999). Disorder-induced critical phenomena in hysteresis: Numerical scaling in three and higher dimensions. *Physical Review B*, *59*(9), 6106–6119. https://doi.org/10.1103/PhysRevB.59.6106

Perković, O., Dahmen, K., & Sethna, J. P. (1995). Avalanches, Barkhausen Noise, and Plain Old Criticality. *Physical Review Letters*, *75*(24), 4528–4531. https://doi.org/10.1103/PhysRevLett.75.4528

Persson, K., Sethuraman, V. A., Hardwick, L. J., Hinuma, Y., Meng, Y. S., Van Der Ven, A., Srinivasan, V., Kostecki, R., & Ceder, G. (2010). Lithium Diffusion in Graphitic Carbon. *The Journal of Physical Chemistry Letters*, *1*(8), 1176–1180. https://doi.org/10.1021/jz100188d

Planes, A., & Vives, E. (2017). Avalanche criticality in thermal-driven martensitic transitions: The asymmetry of the forward and reverse transitions in shape-memory materials. *Journal of Physics: Condensed Matter*, *29*(33), 334001. https://doi.org/10.1088/1361-648X/aa78d7

Safran, S. A. (1987). Stage Ordering in Intercalation Compounds. In *Solid State Physics* (Vol. 40, pp. 183–246). Elsevier. https://doi.org/10.1016/S0081-1947(08)60692-X

Schon, J. C., Adler, D., & Dresselhaus, G. (1988). Theory of staging in intercalation compounds. *Journal of Physics C: Solid State Physics*, *21*(33), 5595–5614. https://doi.org/10.1088/0022-3719/21/33/005

Sethna, J. P., Dahmen, K. A., & Myers, C. R. (2001). Crackling noise. *Nature*, *410*(6825), 242–250. https://doi.org/10.1038/35065675

Sethna, J. P., Dahmen, K., Kartha, S., Krumhansl, J. A., Roberts, B. W., & Shore, J. D. (1993). Hysteresis and hierarchies: Dynamics of disorder-driven first-order phase transformations. *Physical Review Letters*, *70*(21), 3347–3350. https://doi.org/10.1103/PhysRevLett.70.3347

Smith, R. B., Khoo, E., & Bazant, M. Z. (2017). Intercalation Kinetics in Multiphase-Layered Materials. *The Journal of Physical Chemistry C*, *121*(23), 12505–12523. https://doi.org/10.1021/acs.jpcc.7b00185

Thomas, J. M., Millward, G. R., Schlögl, R. F., & Boehm, H. P. (1980). Direct imaging of a graphite intercalate: Evidence of interpenetration of ‘stages’ in graphite: Ferric chloride. *Materials Research Bulletin*, *15*(5), 671–676. https://doi.org/10.1016/0025-5408(80)90149-X

Van Der Ven, A., See, K. A., & Pilon, L. (2022). Hysteresis in electrochemical systems. *Battery Energy*, *1*(2), 20210017. https://doi.org/10.1002/bte2.20210017

Van der Ven, A., Thomas, J. C., Xu, Q., Swoboda, B., & Morgan, D. (2008). Nondilute diffusion from first principles: Li diffusion in ${\text{Li}}_{x}{\text{TiS}}_{2}$. *Physical Review B*, *78*(10), 104306. https://doi.org/10.1103/PhysRevB.78.104306

Vives, E., & Planes, A. (1994a). Avalanches in a fluctuationless first-order phase transition in a random-bond Ising model. *Physical Review B*, *50*(6), 3839–3848. https://doi.org/10.1103/PhysRevB.50.3839

Vives, E., & Planes, A. (1994b). Avalanches in a fluctuationless first-order phase transition in a random-bond Ising model. *Physical Review B*, *50*(6), 3839–3848. https://doi.org/10.1103/PhysRevB.50.3839

Walsh, B., Austvold, S., & Proksch, R. (1998). Magnetic force microscopy of avalanche dynamics in magnetic media. *Journal of Applied Physics*, *84*(10), 5709–5714. https://doi.org/10.1063/1.368835

White, R. A., & Dahmen, K. A. (2003). Driving Rate Effects on Crackling Noise. *Physical Review Letters*, *91*(8), 085702. https://doi.org/10.1103/PhysRevLett.91.085702

Yao, L., & Jack, R. L. (2023). Thermal vestiges of avalanches in the driven random field Ising model. *Journal of Statistical Mechanics: Theory and Experiment*, *2023*(2), 023303. https://doi.org/10.1088/1742-5468/acb7ee
